# A generic assay for whole-genome amplification and deep sequencing of enterovirus A71

**DOI:** 10.1016/j.jviromet.2015.02.011

**Published:** 2015-04

**Authors:** Le Van Tan, Nguyen Thi Kim Tuyen, Tran Tan Thanh, Tran Thuy Ngan, Hoang Minh Tu Van, Saraswathy Sabanathan, Tran Thi My Van, Le Thi My Thanh, Lam Anh Nguyet, Jemma L. Geoghegan, Kien Chai Ong, David Perera, Vu Thi Ty Hang, Nguyen Thi Han Ny, Nguyen To Anh, Do Quang Ha, Phan Tu Qui, Do Chau Viet, Ha Manh Tuan, Kum Thong Wong, Edward C. Holmes, Nguyen Van Vinh Chau, Guy Thwaites, H. Rogier van Doorn

**Affiliations:** a Oxford University Clinical Research Unit, Ho Chi Minh City, Viet Nam; b Children's Hospital 2, Ho Chi Minh City, Viet Nam; c Centre for Tropical Medicine, Nuffield Department of Medicine, University of Oxford, Oxford, UK; d Hospital for Tropical Diseases, Ho Chi Minh City, Viet Nam; e Mahir Bashir Institute for Infectious Diseases & Biosecurity, Charles Perkins Centre, School of Biological Science and Sydney Medical School, The University of Sydney, Sydney, Australia; f University of Malaya, Kuala Lumpur, Malaysia; g Institute of Health and Community Medicine, Universiti Malaysia Sarawak, Sarawak, Malaysia

**Keywords:** Enterovirus A71, Picornavirus, Hand foot and mouth disease, Deep sequencing, Phylogeny

## Abstract

Enterovirus A71 (EV-A71) has emerged as the most important cause of large outbreaks of severe and sometimes fatal hand, foot and mouth disease (HFMD) across the Asia-Pacific region. EV-A71 outbreaks have been associated with (sub)genogroup switches, sometimes accompanied by recombination events. Understanding EV-A71 population dynamics is therefore essential for understanding this emerging infection, and may provide pivotal information for vaccine development. Despite the public health burden of EV-A71, relatively few EV-A71 complete-genome sequences are available for analysis and from limited geographical localities. The availability of an efficient procedure for whole-genome sequencing would stimulate effort to generate more viral sequence data. Herein, we report for the first time the development of a next-generation sequencing based protocol for whole-genome sequencing of EV-A71 directly from clinical specimens. We were able to sequence viruses of subgenogroup C4 and B5, while RNA from culture materials of diverse EV-A71 subgenogroups belonging to both genogroup B and C was successfully amplified. The nature of intra-host genetic diversity was explored in 22 clinical samples, revealing 107 positions carrying minor variants (ranging from 0 to 15 variants per sample). Our analysis of EV-A71 strains sampled in 2013 showed that they all belonged to subgenogroup B5, representing the first report of this subgenogroup in Vietnam. In conclusion, we have successfully developed a high-throughput next-generation sequencing-based assay for whole-genome sequencing of EV-A71 from clinical samples.

## 1 Introduction

Enterovirus A71 (EV-A71) belongs to the Enterovirus A species of the family *Picornaviridae*, and is genetically divided into three genogroups (A, B, and C). The latter two are further divided into subgenogroups, denoted B0 – 5 and C1 – 5, respectively. Since 1997, EV-A71 has emerged as the most important cause of large outbreaks of severe and sometimes fatal hand, foot and mouth disease (HFMD) across the Asia-Pacific region (Solomon et al., 2010; Xing et al., 2014). In Vietnam, EV-A71-related HFMD was first described in 2003, and became a notifiable illness in 2008. Between 2011 and 2012, more than 200,000 hospitalized cases due to HFMD were reported in Vietnam, of which 207 died as a consequence of clinical complications (including cardio-pulmonary compromise with pulmonary edema or hemorrhage) (Khanh et al., 2012; Nguyen et al., 2014). There was a strong association between the detection of EV-A71 and severity and death in this outbreak (Khanh et al., 2012; Nguyen et al., 2014). Although phase III trials of three different inactivated EV-A71 vaccines from China were recently completed with 95% protection rates against EV-A71 associated HFMD (Zhu et al., 2014; Zhu et al., 2013; Li et al., 2014), there are currently no vaccines or specific antiviral drugs available for EV-A71.

Large EV-A71 outbreaks have been associated with (sub)genogroup switches, sometimes accompanied by recombination events (Solomon et al., 2010; McWilliam Leitch et al., 2012; Tee et al., 2010). Similarly, sequence data from Vietnam obtained between 2005 and 2011 show a subgenogroup switch from C5 to C4 in 2011 (Khanh et al., 2012; Tu et al., 2007). Of note, this C4 subgenogroup was also associated with the epidemic in China in 2008 and with a recent outbreak in Cambodia (Xing et al., 2014; Tan et al., 2011; Seiff, 2012). Interestingly, phylogenetic analyses and molecular clock dating suggest that strain replacement commonly occurs in EV-A71 (Tan et al., 2011), and that emerging subgenogroups may circulate cryptically in the community at low prevalence for years and cause mild infection prior to outbreak emergence (Tee et al., 2010)*.* Critically, it is unclear whether viral evolution is the driver of emergence and large outbreaks of HFMD in Southeast Asia, or a consequence of the greater number of infected hosts. The former would have profound implications for vaccine development, necessitating active surveillance and periodic vaccine updates. Taken together the available data highlight the importance of understanding EV-A71 evolution and population dynamics within and between endemic countries, which may be essential for understanding and controlling this emerging infection, including vaccine development.

Despite the public health burden of EV-A71, relatively few (∼500) EV-A71 complete genome sequences are available for analysis and from limited geographical localities, compared to ∼4500 sequences of A/H3N2 human influenza virus alone (Viboud et al., 2013). Hence, the availability of an efficient procedure for whole-genome sequencing would be an important step toward stimulating scientific effort to generate more viral sequence data. Herein, we report the development of a complete genome sequencing protocol for EV-A71 directly from clinical specimens using RT-PCR amplification of three overlapping amplicons and next-generation sequencing technology.

## 2 Materials and methods

### 2.1 Patients and Clinical specimens

Samples used in this study were derived from patients enrolled in an ongoing three-year prospective observational study of HFMD patients of all severities (including out- and in patients) admitted to the outpatient clinics, infectious diseases wards, and the paediatric intensive care units in three major referral hospitals in Ho Chi Minh City, Vietnam. This study utilized 65/82 consecutive EV-A71 RT-PCR positive throat swabs collected in viral transport medium from patients enrolled between July 2013 and December 2013 at the Hospital for Tropical Diseases (HTD) and Children's Hospital 2. For the purpose of assay evaluation, the swabs with a wide range of viral loads were selected based on real time RT-PCR crossing point (Cp) values of between 24 and 36 (i.e. from high to low viral load).

### 2.2 Virus isolates

An EV-A71 subgenogroup C4 isolate from a child with HFMD admitted to HTD during the 2011 outbreak, and eight different isolates each belonging to a different subgenogroup (Table 2) obtained from the Department of Biomedical Science, University of Malaya, Malaysia were used for the initial assessment of assay performance.

### 2.3 Primer design

Overlapping primer pairs were designed for the amplification of three amplicons spanning the entire EV-A71 genome. The primers were derived either from previous publications (Khanh et al., 2012) or newly designed based on the alignment of 279 complete genome sequences of all EV-A71 subgenogroups available in GenBank (Genogroup A, *n* = 1; B0, 2; B1, 14; B2, 4; B3, 8; B4, 11; B5, 18; C1, 17; C2, 33; C3, 3; C4, 165; C5, 2, and undefined, 1; accessed in April 2013). Sequence alignment was performed using MUSCLE available within the Genieous 7.1.3 software package (http://www.geneious.com). The resulting alignment was used to identify conserved regions for selection of PCR primers. The location of primers was chosen to generate PCR fragments with an overlap of 400 nucleotides (nt) or more, allowing for proper sequence assembly. To avoid compromise of PCR sensitivity, a maximum of two degenerate nucleotides were introduced per primer. As a consequence, in some occasions, two or more primers targeting one genomic region were selected for amplification of diverse EV-A71 subgenogroups. Primer sequences, genomic locations, amplified fragment lengths, and alignment profiles of primer binding sites are shown in Table 1 and Supplementary Appendix Fig. 1.

### 2.4 Nucleic acid isolation

Viral RNA was extracted from 140 μl of culture supernatant or clinical material using the QIAamp viral RNA kit (QIAgen GmbH, Hilden, Germany), recovered in 50 μl of elution buffer (provided with the kit) and was either immediately subjected to RT-PCR amplification or stored at −80 °C for subsequent use.

### 2.5 RT-PCR

RT-PCR was performed using SuperScript III One-Step RT-PCR system with Platinum *Taq* DNA High Fidelity (Invitrogen, Carlsbad, CA, USA) in a 25 μl reaction consisting of 800 nM of each primer (Table 1), 12.5 μl of 2× reaction buffer (provided with the kit), 0.5 μl of SuperScriptIII RT/Platinum *Taq* High Fidelity mix and 4 μl of template RNA. RT-PCR reaction was performed in a Mastercycler (Eppendorf, Hamburg, Germany). Cycling conditions are specified in Table 1.

### 2.6 Genome sequencing, sequence assembly and minor variation detection

For each sample, PCR products were quantified by a fluorescence-based dsDNA quantification method using the Quant-iT dsDNA Assay Kit in a Qubit fluorometer (Invitrogen) and then pooled with an equal quantity of each individual PCR amplicon. One nanogram of pooled DNA from individual samples was then subjected to library preparation using the Nextera XT DNA sample preparation kit (Illumina, San Diego, CA, USA), in which each sample was assigned to a unique barcode sequence using the Nextera XT Index Kit (Illumina). Sequencing of the prepared library was carried out using the Miseq reagent kit v2 (300 cycles, Illumina) in an Illumina Miseq platform. A total of 24 samples were sequenced in a single run. The reads obtained were processed to remove PCR primers using CLC Genomics Workbench (QIAgen).

Sequence assembly was performed using the Genieous 7.1.3 software package utilizing a reference-based mapping tool (i.e. the consensus sequence was obtained by mapping individual reads of each sample to a reference sequence). Finally, screening of minor (sub-consensus) variants was performed using the SNP detection tool available in Geneious. A minimum variant frequency of 5% and 500-fold coverage were chosen as cut-off values.

### 2.7 Sequence analysis of the obtained consensuses

Sequence alignment of the consensus genomes obtained in this study was performed using MUSCLE (Edgar, 2004), and the phylogenetic relationships among these data and 25 representative sequences taken from GenBank were estimated using the maximum likelihood (ML) PhyML method available within the Geneious package. The ML phylogenetic analysis utilized the general time reversible (GTR) nucleotide substitution model (Guindon and Gascuel, 2003), and support for individual nodes was assessed using a bootstrap procedure (1000 replicates).

### 2.8 Sequence accession numbers

The sequences of EV-A71 obtained in this study were submitted to NCBI (GenBank) and assigned accession numbers KP691643–KP691666.

### 2.9 Ethical statement

The studies from which clinical specimens were selected for use in this study were reviewed and approved by the Institutional Review Boards of the sites of enrolment in Ho Chi Minh City, Vietnam and the Oxford Tropical Research Ethics Committee (OxTREC), University of Oxford, Oxford, United Kingdom.

## 3 Results

### 3.1 Whole-genome RT-PCRs

After examining the complete genome sequence alignment of EV-A71, primer pairs for three overlapping RT-PCRs spanning the whole genome were designed. To assess their performance, we first tested the assays on RNA derived from culture supernatants of different EV-A71 subgenogroups. Notably, all three RT-PCRs were able to amplify viral RNA from each sample (Table 2).

**Fig. 1:**
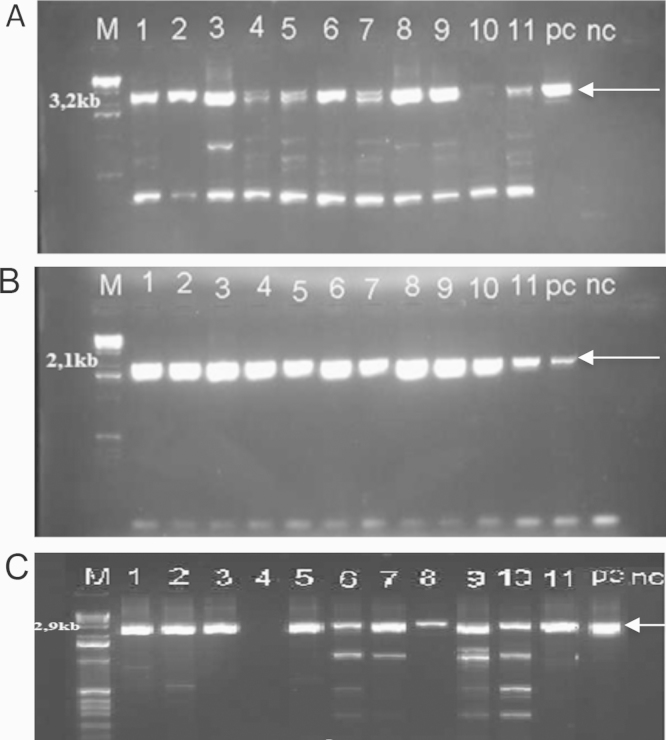
Agarose gel electrophoresis analysis of amplified products from 11 throat swabs from HFMD patients; (A) PCR 1; (B) PCR 2; (C) PCR 3; lanes 1-11: clinical samples, PC: positive control; NC: negative control; amplified products of expected sizes are indicated by arrows.

**Fig. 2:**
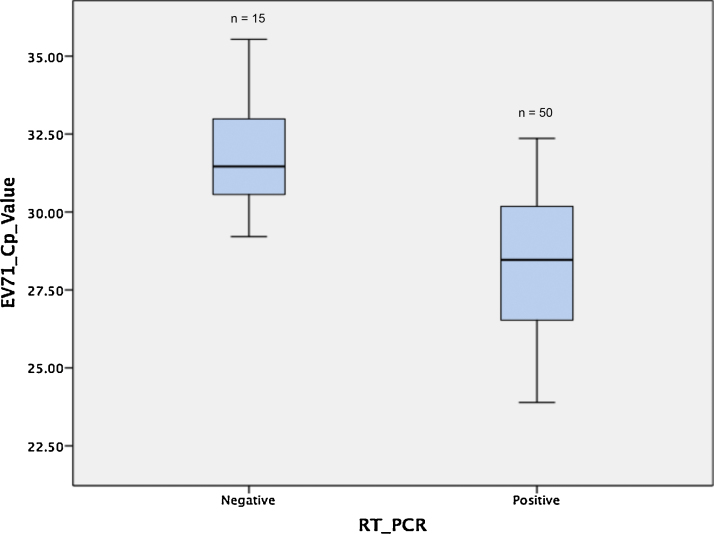
Boxplots showing Cp values generated by an EV-A71 real time RT-PCR of RT-PCR positive and negative groups, Cp value median; range: 28.2; 23.9–32.4 versus 31.2; 29.2–35.5; *P* < 0.001.

The overlapping PCRs were further tested on 65 EV-A71 RT-PCR-positive throat swab samples collected from children with HFMD. All three fragments were successfully amplified in 50/65 (77%) samples (Fig. 1). Eleven out of 15 samples that were not amplified by the three overlapping PCRs were due to failure of the 3.2Kb PCRs (details in Supplementary Appendix Table 1). A significantly higher viral load (as suggested by Cp values generated by an EV-A71 real-time RT-PCR (Khanh et al., 2012)) was observed among samples that were amplified by all three RT-PCRs compared to samples that were not (Fig. 2). Accordingly, clinical samples with a Cp value of 30 or less allowed the successful amplification of the three genomic fragments.

### 3.2 Genome sequencing using the Illumina platform

Amplified products from 23 clinical samples and an EV-A71 isolate were sequenced in one batch. The run resulted in an output of 4.9 Gb, of which 60% could be successfully assembled. Over 92% of the assembled bases had Phred quality scores of ≥30 (i.e. a probability of ≥99.9% that the single bases were correctly sequenced). The remaining bases had Phred quality scores of between >10 and 29 (i.e. >90% of accuracy). Twenty-three complete genome sequences (including 22 from clinical samples and one from virus culture material) and one near-complete genome sequence of EV-A71 were successfully assembled. A coverage of ≥500-fold was achieved in 22 samples (including 21 clinical samples and the virus isolate) and >80-fold in one other. Details of sequencing output per sample are presented in Supplementary Appendix Table 2.

**Fig. 3:**
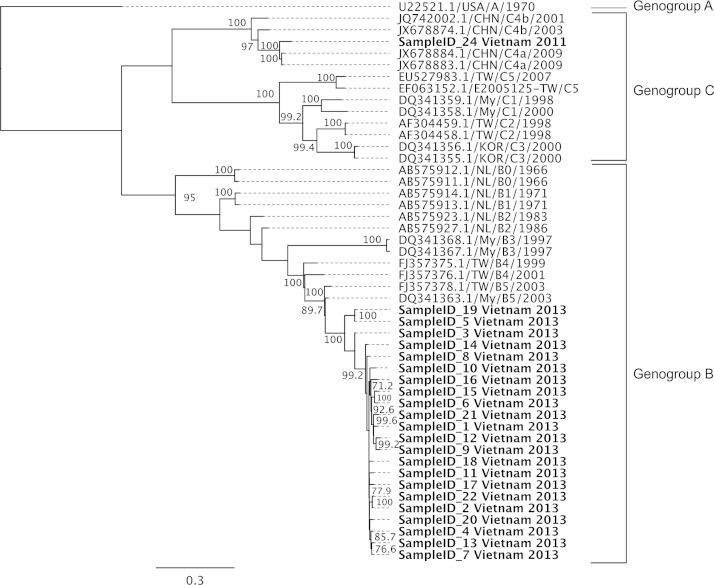
ML phylogeny based on complete genomes (in bold) obtained in this study and those of representatives of EV-A71 downloaded from the GenBank. A similar result was obtained when VP1 sequences were analyzed separately (data not shown). The tree is rooted on a single genogroup A sequence (USA/A/1970) and all horizontal branch lengths are drawn to a scale of nucleotide substitutions per site. Bootstraps >70% are also shown. My: Malaysia, TW: Taiwan, NL: the Netherlands, KOR: Korea, CHN: China.

Phylogenetic analysis suggested that all viruses derived from throat swabs collected in 2013 belonged to EV-A71 subgenogroup B5 (Fig. 3), while the virus from culture material belonged to subgenogroup C4. This is in agreement with our initial typing result based on sequencing of VP1 region (data not shown).

### 3.3 Minor variant detection

Using the criteria described in the Methods section, we identified a total of 107 positions in the EV-A71 genome carrying minor variants (ranging from 0 to 15 variants per sample), of which 15 (14%) were nonsynonymous. Interestingly, 73% (11/15) of the nonsynonymous variants were present in the nonstructural protein coding region, which also contained 65% of the total 107 variants observed, with mutations in the 2 C protein being the next most frequent (38/107, 35.5%). Most variants (101) represented minor variants. However six variants were found to be either minor or dominant (i.e. >50%) variants in different samples, suggesting that they may impact viral fitness in some way, and hence merit additional investigation. More details on the minor variants are presented Supplementary Appendix Table 3, including the frequency of the six variants shared between viruses found in the data set of 279 genome sequences used in the present study.

## 4 Discussion

We have successfully developed a high-throughput Illumina Miseq-based generic assay for direct whole-genome sequencing of EV-A71 from clinical samples. Although primers for whole-genome amplification of EV-A71 have been proposed previously (Shih et al., 2000; Cordey et al., 2012; Huang et al., 2009; Yoke-Fun and AbuBakar, 2006; Chang et al., 2012; Zhang et al., 2013), these primer sets were either large (i.e. at least eight combinations were used) and/or not tested on diverse EV-A71 subgenogroups. The procedure described here only requires three overlapping PCRs to amplify the entire virus genome directly from clinical specimens. In addition, the entire procedure from nucleic acid extraction and PCR amplification to obtaining a total of between 24 and 96 virus genomes takes approximately 5 days (Flowchart 1). The total reagent-associated cost is $120 USD per sample if 24 samples are multiplexed in one run (date of cost assessment: July 2014). This can be reduced to $70 USD if 96 samples are combined. In addition, with the great sequencing depth (i.e. a single nucleotide is sequenced at least 500 times), the extent and pattern of intra-host genetic diversity data can be explored in addition to the consensus sequence. It should however be noted that although the current cost per genome is affordable, the total cost per run (∼$3000 USD) together with hardware-associated cost combined with bioinformatics challenges may remain the major obstacles preventing Illumina based whole-genome sequencing assay from being widely used, in particular in developing countries including parts of Southeast Asia where EV-A71 and HFMD are endemic. Alternatively, generating full-length virus sequence can be achieved using Sanger-sequencing-based strategies (e.g. DNA walking of the obtained PCR amplicons described in the present study and/or multiple small overlapping PCRs spanning the whole virus genome). While the associated cost per genome of such strategies might be comparable with that of Illumina Miseq one, generating full-length virus genome using Sanger sequencing technology is a laborious procedure as it not possible to multiplex between 24 and 96 samples per run. Likewise, with Sanger-sequencing data, exploring the pattern of intra-host diversity of the pathogen at a level of frequency of 5% is unachievable.

Using our procedure we were able to sequence viruses of subgenogroup C4 and B5. Likewise, RNA from culture materials of diverse EV-A71 subgenogroups belonging to both genogroup B and C (Table 2) that have been associated with outbreaks of HFMD in the Asia-Pacific region since the 1990s was successfully amplified. Assuming that a Cp value of ∼30 is the threshold of assay limit of detection (Fig. 2), our observational data on Cp values obtained from 824 throat swabs from HFMD patients in Vietnam (data not shown) show that 75% of viruses in RT-PCR positive throat swabs can be sequenced by our assay. Of note, among 65 tested swabs, in the majority of cases the procedure failure was attributed to the RT-PCRs that generated the largest amplicon (RT-PCR 1) and/or targeted at the genetically variable regions of the virus genome (RT-PCR 2) (Supplementary Appendix Table 1), which may suggest that fragment size and/or sequence variations play a part in addition to the viral-load factor.

We conservatively chose cut-off values of 500-fold coverage and a frequency of 5% for detection of minor variants. This is well above the next-generation sequencing error rate of 0.1% (Archer et al., 2012), while a previous study suggested that a coverage of >400-fold is required for reliable detection of minor variants present at 1% in next generation sequencing data (Wang et al., 2007).

Although an association between EV-A71 subgenogroups and disease severity has not been identified, previous studies have shown potential associations between single nucleotide variants and clinical severity (Cordey et al., 2012; Zhang et al., 2014). In addition, a recent study of C4, B1, B2 and B5 sequences found that nonsynonymous and synonymous substitutions occurred more frequently in the nonstructural than the structural protein-coding region, suggesting that the former was a major fitness determinant (Huang et al., 2014). We similarly observed a majority of minor variants (including nonsynonymous ones) in the nonstructural protein-coding region, although the function of these mutations is uncertain. As a consequence, these data further emphasize the need to study viral diversity and evolution at the whole-genome level, rather than in the VP1 protein alone which has been the main focus to date. Likewise, exploring the association between specific viral genotypes/single nucleotide polymorphisms and clinical phenotype is clearly an area of importance, although beyond the scope of the present study.

Our analysis of EV-A71 strains sampled in 2013 showed that they all belonged to subgenogroup B5, representing the first report of this subgenogroup in Vietnam, while subgenogroup C4 caused the large outbreak of HFMD that occurred in Vietnam in 2011–12 (Khanh et al., 2012). Subgenogroup switches commonly occur in other countries of Asia-Pacific region where EV-A71 and HFMD are endemic (Solomon et al., 2010), although the evolutionary and epidemiological processes responsible for these switches are still unclear. Taken together, these data highlight the importance of studying spatial and temporal dynamics of EV-A71 across endemic countries, which may in turn inform the development of intervention strategies, including vaccine development and implementation, and can now be facilitated by the availability of a high throughput/cost-effective whole-genome sequencing procedure.

In conclusion, we have successfully developed a high-throughput next-generation sequencing-based generic assay for direct whole-genome sequencing of EV-A71 from clinical samples that provides important insights into viral diversity and evolution.

## Funding

The research leading to these results has received funding from the Wellcome Trust of Great-Britain (101104/Z/13/Z and 089276/Z/09/Z), and the Li Ka Shing Foundation–University of Oxford Global Health Program strategic award (LG23). ECH is supported by an NHMRC Australia Fellowship. OKC is supported by a High Impact Research Grant (H20001-E00004) from the Ministry of Education, Malaysia Government and University of Malaya Research Grant (RG480/12HTM). The funders had no role in study design, data collection and analysis, decision to publish, or preparation of the manuscript.

## Conflict of interest

No conflict of interest.

## Figures and Tables

**Flowchart 1:**
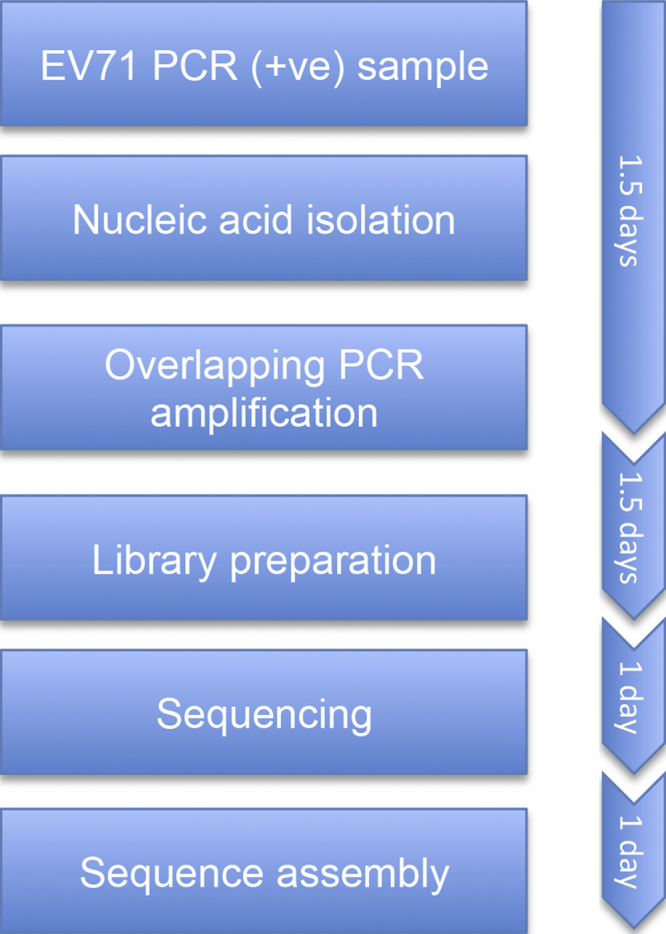
Chart showing analysis procedure for obtaining the complete genome of EV-A71 from clinical samples.

**Table 1:** Primer sequences, location and size of amplified products.

RT-PCR^a^	Primer	Oligo sequence (5′–3′)	Orientation	Start–end^b^	Genomic locations	Product size (Kb)
1	FL5F	TTAAAACAGCCTGTGGGTTGYACC	Forward	1–24	5′UTR	3.2
	FL9R	ACTAAAGGGTACTTGGACTTVGA	Reverse	3180–3158	VP1	

2	FL15F	GGATTRGTWGGGGAGATAGACCT	Forward	2699–2721	VP1	2.1
	FL16F	GGATTRGTWGGAGAGATAGACCT	Forward	2699–2721	VP1	
	FL17F	GGATTRGTWGGGGAGATAGATCT	Forward	2699–2721	VP1	
	FL18F	GGATTRGTWGGAGAGATAGATCT	Forward	2699–2721	VP1	
	FL19R	GTCACTTCAATRTCRCAGTCCAT	Reverse	4827–4805	2C	
	FL20R	GTCACTTCAATRTCRCAATCCAT	Reverse	4827–4805	2C	

3	FL10F	CAAACACCGWATTGAACCTGT	Forward	4423–4443	2C	2.9
	FL3R2	GCTATTCTGGTTATAACAAATTTACC	Reverse	7405–7380	3′UTR	

*Note*:

a Thermal cycling conditions of RT-PCR 1: 1 cycle of 60 **°**C, 3 min, 53 **°**C, 30 min, 94 **°**C, 2 min, 45 cycles of 94 **°**C, 15 s, 53 **°**C, 30 s, 68 **°**C, 4 min and 1 cycle of 68 **°**C, 5 min; and RT-PCR 2 and 3: 1 cycle of 60 **°**C, 3 min, 50 **°**C, 30 min, 94 **°**C, 2 min, 45 cycles of 94 **°**C, 15 s, 50 **°**C, 30 s, 68 **°**C, 3.5 min and 1 cycle of 68 **°**C, 5 min.

b Positions of primers are given based on the genomic sequence of an EV-A71 strain from Vietnam, Vietnam/540 V/VNM/05/C4 (GenBank accession JQ965759.1); Y = C/T, V = A/C/G, R = A/G, W = A/T;

**Table 2:** Virus isolates and results of overlapping RT-PCRs.

EV-A71 strain	Subgeno-group	Accession number	Location	Year of isolation	Overlapping RT-PCRs	Subjected to Miseq sequencing
9522	C1	AY258300	Malaysia	2003	Positive	No
8M/6/99	C2	AY126012	Australia	1999	Positive	No
001-KOR-00	C3	AY125966	Korea	2000	Positive	No
VN152	C4	Not available	Vietnam	2011	Positive	Yes
VN5559	C4	AM490152	Vietnam	2005	Positive	No
VN5784	C5	AM490158	Vietnam	2005	Positive	No
13903	B3	AY207648	Malaysia	1997	Positive	No
A10/4	B4	AF376067	Malaysia	2000	Positive	No
18431	B5	Not available	Malaysia	2006	Positive	No

## References

[bib0110] Archer J., Baillie G., Watson S.J., Kellam P., Rambaut A., Robertson D.L. (2012). Analysis of high-depth sequence data for studying viral diversity: a comparison of next generation sequencing platforms using Segminator II. BMC Bioinformatics.

[bib0100] Chang S.C., Li W.C., Chen G.W., Tsao K.C., Huang C.G., Huang Y.C., Chiu C.H., Kuo C.Y., Tsai K.N., Shih S.R., Lin T.Y. (2012). Genetic characterization of enterovirus 71 isolated from patients with severe disease by comparative analysis of complete genomes. J. Med. Virol..

[bib0085] Cordey S., Petty T.J., Schibler M., Martinez Y., Gerlach D., van Belle S., Turin L., Zdobnov E., Kaiser L., Tapparel C. (2012). Identification of site-specific adaptations conferring increased neural cell tropism during human enterovirus 71 infection. PLoS Pathog..

[bib0070] Edgar R.C. (2004). MUSCLE: multiple sequence alignment with high accuracy and high throughput. Nucleic Acids Res..

[bib0075] Guindon S., Gascuel O. (2003). A simple, fast, and accurate algorithm to estimate large phylogenies by maximum likelihood. Syst. Biol..

[bib0125] Huang S.W., Cheng H.L., Hsieh H.Y., Chang C.L., Tsai H.P., Kuo P.H., Wang S.M., Liu C.C., Su I.J., Wang J.R. (2014). Mutations in the non-structural protein region contribute to intra-genotypic evolution of enterovirus 71. J. Biomed. Sci..

[bib0090] Huang S.W., Hsu Y.W., Smith D.J., Kiang D., Tsai H.P., Lin K.H., Wang S.M., Liu C.C., Su I.J., Wang J.R. (2009). Reemergence of enterovirus 71 in 2008 in taiwan: dynamics of genetic and antigenic evolution from 1998 to 2008. J. Clin. Microbiol..

[bib0015] Khanh T.H., Sabanathan S., Thanh T.T., Thoa le P.K., Thuong T.C., Hang V., Farrar J., Hien T.T., Chau N., van Doorn H.R. (2012). Enterovirus 71-associated hand, foot, and mouth disease, Southern Vietnam, 2011. Emerg. Infect. Dis..

[bib0035] Li R., Liu L., Mo Z., Wang X., Xia J., Liang Z., Zhang Y., Li Y., Mao Q., Wang J., Jiang L., Dong C., Che Y., Huang T., Jiang Z., Xie Z., Wang L., Liao Y., Liang Y., Nong Y., Liu J., Zhao H., Na R., Guo L., Pu J., Yang E., Sun L., Cui P., Shi H., Wang J., Li Q. (2014). An inactivated enterovirus 71 vaccine in healthy children. N. Engl. J. Med..

[bib0040] McWilliam Leitch E.C., Cabrerizo M., Cardosa J., Harvala H., Ivanova O.E., Koike S., Kroes A.C., Lukashev A., Perera D., Roivainen M., Susi P., Trallero G., Evans D.J., Simmonds P. (2012). The association of recombination events in the founding and emergence of subgenogroup evolutionary lineages of human enterovirus 71. J. Virol..

[bib0020] Nguyen N.T., Pham H.V., Hoang C.Q., Nguyen T.M., Nguyen L.T., Phan H.C., Phan L.T., Vu L.N., Tran Minh N.N. (2014). Epidemiological and clinical characteristics of children who died from hand, foot and mouth disease in Vietnam, 2011. BMC Infect. Dis..

[bib0060] Seiff A. (2012). Cambodia unravels cause of mystery illness. Lancet.

[bib0080] Shih S.R., Ho M.S., Lin K.H., Wu S.L., Chen Y.T., Wu C.N., Lin T.Y., Chang L.Y., Tsao K.C., Ning H.C., Chang P.Y., Jung S.M., Hsueh C., Chang K.S. (2000). Genetic analysis of enterovirus 71 isolated from fatal and non-fatal cases of hand, foot and mouth disease during an epidemic in Taiwan, 1998. Virus Res..

[bib0005] Solomon T., Lewthwaite P., Perera D., Cardosa M.J., McMinn P., Ooi M.H., Virology (2010). epidemiology, pathogenesis, and control of enterovirus 71. Lancet Infect. Dis..

[bib0055] Tan X., Huang X., Zhu S., Chen H., Yu Q., Wang H., Huo X., Zhou J., Wu Y., Yan D., Zhang Y., Wang D., Cui A., An H., Xu W. (2011). The persistent circulation of enterovirus 71 in People's Republic of China: causing emerging nationwide epidemics since 2008. PLoS ONE.

[bib0045] Tee K.K., Lam T.T., Chan Y.F., Bible J.M., Kamarulzaman A., Tong C.Y., Takebe Y., Pybus O.G. (2010). Evolutionary genetics of human enterovirus 71: origin, population dynamics, natural selection, and seasonal periodicity of the VP1 gene. J. Virol..

[bib0050] Tu P.V., Thao N.T., Perera D., Huu T.K., Tien N.T., Thuong T.C., How O.M., Cardosa M.J., McMinn P.C. (2007). Epidemiologic and virologic investigation of hand, foot, and mouth disease, southern Vietnam, 2005. Emerg. Infect. Dis..

[bib0065] Viboud C., Nelson M.I., Tan Y., Holmes E.C. (2013). Contrasting the epidemiological and evolutionary dynamics of influenza spatial transmission. Philos. Trans. R. Soc. Lond. B: Biol. Sci..

[bib0115] Wang C., Mitsuya Y., Gharizadeh B., Ronaghi M., Shafer R.W. (2007). Characterization of mutation spectra with ultra-deep pyrosequencing: application to HIV-1 drug resistance. Genome Res..

[bib0010] Xing W., Liao Q., Viboud C., Zhang J., Sun J., Wu J.T., Chang Z., Liu F., Fang V.J., Zheng Y., Cowling B.J., Varma J.K., Farrar J.J., Leung G.M., Yu H. (2014). Hand, foot, and mouth disease in China, 2008–12: an epidemiological study. Lancet Infect. Dis..

[bib0095] Yoke-Fun C., AbuBakar S. (2006). Phylogenetic evidence for inter-typic recombination in the emergence of human enterovirus 71 subgenotypes. BMC Microbiol..

[bib0120] Zhang B., Wu X., Huang K., Li L., Zheng L., Wan C., He M.L., Zhao W. (2014). The variations of VP1 protein might be associated with nervous system symptoms caused by enterovirus 71 infection. BMC Infect. Dis..

[bib0105] Zhang Y., Tan X., Cui A., Mao N., Xu S., Zhu Z., Zhou J., Shi J., Zhao Y., Wang X., Huang X., Zhu S., Zhang Y., Tang W., Ling H., Xu W. (2013). Complete genome analysis of the C4 subgenotype strains of enterovirus 71: predominant recombination C4 viruses persistently circulating in China for 14 years. PLoS ONE.

[bib0030] Zhu F.C., Meng F.Y., Li J.X., Li X.L., Mao Q.Y., Tao H., Zhang Y.T., Yao X., Chu K., Chen Q.H., Hu Y.M., Wu X., Liu P., Zhu L.Y., Gao F., Jin H., Chen Y.J., Dong Y.Y., Liang Y.C., Shi N.M., Ge H.M., Liu L., Chen S.G., Ai X., Zhang Z.Y., Ji Y.G., Luo F.J., Chen X.Q., Zhang Y., Zhu L.W., Liang Z.L., Shen X.L. (2013). Efficacy, safety, and immunology of an inactivated alum-adjuvant enterovirus 71 vaccine in children in China: a multicentre, randomised, double-blind, placebo-controlled, phase 3 trial. Lancet.

[bib0025] Zhu F., Xu W., Xia J., Liang Z., Liu Y., Zhang X., Tan X., Wang L., Mao Q., Wu J., Hu Y., Ji T., Song L., Liang Q., Zhang B., Gao Q., Li J., Wang S., Hu Y., Gu S., Zhang J., Yao G., Gu J., Wang X., Zhou Y., Chen C., Zhang M., Cao M., Wang J., Wang H., Wang N. (2014). Efficacy, safety, and immunogenicity of an enterovirus 71 vaccine in China. N. Engl. J. Med..

