# Supplementary material for: A generic assay for whole-genome amplification and deep sequencing of enterovirus A71
Source: J Virol Methods. 2015 Apr;215-216:30–6. doi: 10.1016/j.jviromet.2015.02.011 (PMC4374682; doi:10.1016/j.jviromet.2015.02.011)
Supplement: Supplementary file 1 [file mmc1.pdf]

	10 · 20
Consensus	TTAAAACAGCCTGTGGGTTGCACC
	FL5F; Primer Bind
1. GU434678.1/China/Hubei/EV71-Hubei-09-China/B5/2009 (B5)	TTAAAACAGCCTGTGGGTTGCACC
2. AB204853.1/BrCr-ts/A (A)	TTAAAACAGCCTGTGGGTTGCACC
3. AB575911.1/Netherlands/10076/B0/1966 (B0)	
4. AB575912.1/Netherlands/10857/B0/1966 (B0)	
5. DQ341354.1/Singapore/3799-SIN-98/B3/1998 (B3)	TTAAAACAGCCTGTGGGTTGCACC
6. DQ341368.1/Malaysia/MY104-9-SAR-97/B3 (B3)	TTAAAACAGCCTGTGGGTTGCACC
7. DQ341367.1/Malaysia/MY821-3-SAR-97/B3/1997 (B3)	TTAAAACAGCCTGTGGGTTGCACC
8. AB550335.1/Malaysia/Labstrain/SK-EV006-LPS1/B3/1997 (B3)	TTAAAACAGCCTGTGGGTTGCACC
9. AB550334.1/Malaysia/SK-EV006-org/B3/1997 (B3)	TTAAAACAGCCTGTGGGTTGCACC
10. AB469182.1/Malaysia/SK-EV006/Malaysia/97 (B3)	TTAAAACAGCCTGTGGGTTGCACC
11. JQ950555.1/Australia26M/AUS/4/99/GuaR1/B3/2010 (B3)	
12. EU364841.1/Australia/26M/AUS/4/99/B3/1999 (B3)	TTAAAACAGCCTGTGGGTTGCACC
13. EF373575.1/Taiwan/E2002042-TW-CDC (B4)	TTAAAACAGCCTGTGGGTTGCACC
14. FJ357377.1/Taiwan/S0296-TW00/B4/2000 (B4)	TTAAAACAGCCTGTGGGTTGCACC
15. FJ357376.1/Taiwan/S0318-TW01/B4/2001 (B4)	TTAAAACAGCCTGTGGGTTGCACC
16. DQ341366.1/Malaysia/SB2864-SAR-00/B4/2000 (B4)	TTAAAACAGCCTGTGGGTTGCACC
17. DQ341365.1/Malaysia/PP37-MAL-01/B4/2001 (B4)	TTAAAACAGCCTGTGGGTTGCACC
18. AF316321.2/Singapore/5865/sin/000009/B4 (B4)	TTAAAACAG-CTGTGGGTTGTACC
19. AF352027.1/Singapore/5666/sin/002209/B4 (B4)	TTAAAACAG-CTGTGGGTTGTACC
20. FJ357375.1/Taiwan/N7008-TW99/B4/1999 (B4)	TTAAAACAGCCTGTGGGTTGCACC
21. AB550337.1/Japan/Labstrain/C7/Osaka-LPS1/B4/1997 (B4)	TTAAAACAGCCTGTGGGTTGCACC
22. AB550336.1/Japan/C7/Osaka-org/B4/1997 (B4)	TTAAAACAGCCTGTGGGTTGCACC
23. FJ461781.1/Singapore/NUH0083/SIN/08/B5/2008 (B5)	
24. JF738001.1/Thailand/THA-EV71-019/B5/2009 (B5)	
25. DQ341364.1/Singapore/5511-SIN-00 (B5)	TTAAAACAGCCTGTGGGTTGTACC
26. JN992282.1/Brunei/BRU/2006/35334 (B5)	TTAAAACAGCCTGTGGGTTGCACC
27. FJ357378.1/Taiwan/N2838-TW03/B5/2003 (B5)	TTAAAACAGCCTGTGGGTTGCACC
28. DQ341363.1/Malaysia/S19841-SAR-03/B5/2003 (B5)	TTAAAACAGCCTGTGGGTTGCACC
29. DQ341362.1/Malaysia/SB12736-SAR-03/B4/2003 (B4)	TTAAAACAGCCTGTGGGTTGCACC
30. JN964686.1/China/Fujian/EV71/Xiamen/2009/B5/2009 (B5)	TTAAAACAGCCTGTGGGTTGCACC
31. GQ231942.1/Taiwan/TW/96016/08/B5 (B5)	TTAAAACAG-CTGTGGGTTGCACC
32. GQ231925.1/Taiwan/TW/1101/08/B5 (B5)	TTAAAACAG-CTGTGGGTTGCACC
33. GQ231934.1/Taiwan/TW/70811/08/B5 (B5)	TTAAAACAG-CTGTGGGTTGCACC
34. GQ231943.1/Taiwan/TW/96022/08/B5 (B5)	TTAAAACAG-CTGTGGGTTGCACC
35. GQ231935.1/Taiwan//TW/70886/08/B5 (B5)	TTAAAACAG-CTGTGGGTTGCACC
36. GQ231941.1/Taiwan/TW/96002/08/B5 (B5)	TTAAAACAG-CTGTGGGTTGCACC
37. EU527985.1/Taiwan/2007-08747/2007/B5/2007 (B5)	
38. FJ357385.1/Taiwan/M0380-TW08/B5/2008 (B5)	TTAAAACAGCCTGTGGGTTGCACC
39. HM622390.1/Taiwan/2009-03531/B5/2009 (B5)	
40. GQ231936.1/Taiwan/TW/70902/08/B5 (B5)	TTAAAACAG-CTGTGGGTTGCACC
41. AB575927.1/Netherlands/11316/B2/1986 (B2)	
42. AB575928.1/Netherlands/11590/B2/1986 (B2)	
43. AB575923.1/Netherlands/20233/B2/1983 (B2)	
44. U22522.1/USA/MS/7423/87/B2/1987 (B2)	TTAAAACAG-CTGTGGGTTGTTCC
45. FJ357384.1/Taiwan/266-TW86/B1/1986 (B1)	TTAAAACAGCCTGTGGGTTGTTCC
46. FJ357383.1/Taiwan/252-TW86/B1/1986 (B1)	TTAAAACAGCCTGTGGGTTGTTCC
47. FJ357380.1/Taiwan/237-TW86/B1/1986 (B1)	TTAAAACAGCCTGTGGGTTGTTCC
48. FJ357379.1/Taiwan/236-TW86/B1/1986 (B1)	TTAAAACAGCCTGTGGGTTGTTCC
49. FJ357382.1/Taiwan/238-TW86/B1/1986 (B1)	TTAAAACAGCCTGTGGGTTGTTCC
50. FJ357381.1/Taiwan/244-TW86/B1/1986 (B1)	TTAAAACAGCCTGTGGGTTGTTCC
51. AB575914.1/Netherlands/17000/B1/1971 (B1)	
52. AB575913.1/Netherlands/11977/B1/1971 (B1)	
53. AB575918.1/Netherlands/20574/B1/1978 (B1)	
54. AB575917.1/Netherlands/10196/B1/1977 (B1)	
55. HQ189392.1/Hungary/HUN/1978/1978 (B1)	TTAAAACAGCCTGTGGGTTGTTCC
56. AB575916.1/Netherlands/16173/B1/1976 (B1)	
57. AB575915.1/Netherlands/9443/B1/1974 (B1)	
58. AB482183.1/Japan/Nagoya/B1/1973 (B1)	TTAAAACAGCCTGTGGGTTGTTCC
59. HQ423143.1/China/Yunnan/KM186/09/2009/C4a/2009 (C4)	TTAAAACAGCCTGTGGGTTGCACC
60. HQ423142.1/China/KMM/09/2009 (C4)	TTAAAACAGCCTGTGGGTTGCACC
61. JQ742001.1/China/Guangxi/AFP2001064/EV71/GX/CHN/2001/C4b/2001 (C4)	TTAAAACAGCCTGTGGGTTGTACC
62. EU131776.1/Taiwan/N3340-TW-02/C4b/2002 (C4)	TTAAAACAGCCTGTGGGTTGCACC
63. JX678885.1/China/Shanghai/SH-17/SH/CHN/2002/C4b/2002 (C4)	TTAAAAC-GCCTGTGGGTTGCACC
64. JQ742002.1/China/AFP2001071/EV71/GX/CHN/2001/C4b/2001 (C4)	TTAAAACAGCCTGTGGGTTGTACC
65. GU350629.1/China/AnHui/Fuyang-0805a (C4)	
66. FJ194964.1/China/Guangdong/EV71/GDFS/3/2008/C4b/2008 (C4)	TTAAAACAGCCTGTGGGTTGCACC
67. GQ994989.1/China/Chongqing/Chongqing1-09-China/2009/C4b/2009 (C4)	TTAAAACAGCCTGTGGGTTGCACC
68. HM807310.1/Taiwan/cmuh-050530-5/2005 (C4)	TTAAAACAG-CTGTGGGTTGTACC
69. AY465356.1/China/Shenzhen/SHZH03/C4b/2003 (C4)	TTAAAACAG-CTGTGGGTTGCACC
70. JX678874.1/China/Chongqing/CQ03-1/CQ/CHN/2003/C4b/2003 (C4)	TTAAAAC-GCCTGTGGGTTGCACC
71. HQ647171.1/Canada/EV141-06/2006/C4a/2006 (C4)	TTAAAACAG-CTGTGGGTTGCACC
72. HQ647180.1/Canada/EV034-06/2006 (C4)	TTAAAACAG-CTGTGGGTTGCACC

	10 20
Consensus	TTAAAACAGCCTGTGGGTTGCACC
	FL5F; Primer Bind
71. HQ647171.1/Canada/EV141-06/2006/C4a/2006 (C4)	[illegible]
72. HQ647180.1/Canada/EV034-06/2006 (C4)	TTAAAACAG-CTGTGGGTTGCACC
73. HQ647179.1/Canada/EV144-06/2006 (C4)	TTAAAACAG-CTGTGGGTTGCACC
74. JX678886.1/China/Shanghai/SH-6/SH/CHN/2002/C4b/2002 (C4)	TTAAAAC-GCCTGTGGGTTGCACC
75. AB550339.1/Japan/Labstrain/75-Yamagata-LPS1/C4/2003 (C4)	TTAAAACAGCCTGTGGGTTGCACC
76. AB550338.1/Japan/75-Yamagata-org/C4/2003 (C4)	TTAAAACAGCCTGTGGGTTGCACC
77. DQ133459.1/Taiwan/1235/C4/2004 (C4)	TTAAAACAG-CTGTGGGTTGCACC
78. DQ133458.1/Taiwan/984/C4/2004 (C4)	TTAAAACAG-CTGTGGGTTGTACC
79. FJ357373.1/Taiwan/S0584-TW04/C4/2004 (C4)	TTAAAACAGCCTGTGGGTTGCACC
80. EF373576.1/Taiwan/E2004104-TW-CDC (C4)	TTAAAACAGCCTGTGGGTTGCACC
81. GQ231938.1/TaiwanTW/71552/05 (C4)	TTAAAACAG-CTGTGGGTTGCACC
82. GQ231926.1/Taiwan/TW/1956/05/C4a/2005 (C4)	TTAAAACAG-CTGTGGGTTGCACC
83. GQ231940.1/Taiwan/TW/72232/04/C4 (C4)	TTAAAACAG-CTGTGGGTTGCACC
84. GQ231937.1/Taiwan/TW/71428/05/C4 (C4)	TTAAAACAG-CTGTGGGTTGCACC
85. GQ231939.1/Taiwan/TW/71595/04/C4 (C4)	TTAAAACAG-CTGTGGGTTGCACC
86. GQ231929.1/Taiwan/TW/2728/04/C4a/2004 (C4)	TTAAAACAG-CTGTGGGTTGCACC
87. FJ357374.1/Taiwan/N2121-TW05/C4/2005 (C4)	TTAAAACAGCCTGTGGGTTGCACC
88. GQ231931.1/Taiwan/TW/2824/04/C4 (C4)	TTAAAACAG-CTGTGGGTTGCACC
89. GQ231928.1/Taiwan/TW/2639/04/C4 (C4)	TTAAAACAG-CTGTGGGTTGCACC
90. GQ231927.1/Taiwan/TW/2429/04/C4 (C4)	TTAAAACAG-CTGTGGGTTGCACC
91. GQ231930.1/Taiwan/TW/2815/04/C4 (C4)	TTAAAACAG-CTGTGGGTTGCACC
92. JQ965759.1/Vietnam/540V/VNM/05/C4/2005 (C4)	TTAAAACAGCCTGTGGGTTGCACC
93. GQ231932.1/Taiwan/TW/2871/04/C4 (C4)	TTAAAACAG-CTGTGGGTTGCACC
94. GQ994992.1/China/Henan/Henan2-09-China/2009/C4a/2009 (C4)	AAGCTGTGGGTTGCACC
95. GQ279369.1/China/SZ/HK08-5/2008 (C4)	TTGAAACAGCCTGTGGGTTGCACC
96. JX244184.1/China/SDLY48/C/2009 (C4)	TTAAAACAGCCTGTGGGTTGCACC
97. FJ606447.1/China/Beijing/BJ08-Z004-3/2008/C4a/2008 (C4)	TTAAAACAGCCTGTGGGTTGCACC
98. JQ681218.1/China/Henan/H8-1/C4a/2008 (C4)	TTAAAACAGCCTGTGGGTTGCACC
99. GU196833.1/China/Henan1-09-China/2009 (C4)	
100. HQ129932.1/China/BJ06-SJS06/06/C4/2006 (C4)	
101. FJ607336.1/China/28/SHENZHEN/08/China/HFMD/2008/2008 (C4)	TTAAAACAG-CTGTGGGTTGCACC
102. FJ828519.1/China/BJ08/C4/2008 (C4)	CC
103. FJ360545.1/China/GZ-08-02/C4/2008 (C4)	TTAAAACAG-CTGTGGGTTGTACC
104. JX244182.1/China/SDLY1/2009 (C4)	TTAAAACAGCCTGTGGGTTGCACC
105. JF820312.1/China/LCH01/C4/2010 (C4)	
106. HM002488.1/China/BJ366/2009 (C4)	TTAAAACAGCCTGTGGGTTGCACC
107. GQ231933.1/Taiwan/TW/70516/08/C4a/2008 (C4)	TTAAAACAG-CTGTGGGTTGCACC
108. KC109780.1/China/202/Jingdezhen/China/HFMDSevere/2011/C4/2011 (C4)	TTAAAACAGCCTGTGGGTTGCACC
109. HM002485.1/China/BJ972008 (C4)	TTAAAACAGCCTGTGGGTTGCACC
110. GU366191.1/China/Henan/Henan10-08-China/2008/C4a/2008 (C4)	TTAAAACAGCCTGTGGGTTGCACC
111. FJ713137.1/Shanghai/Shanghai-036-2009/C4a/2009 (C4)	TTAAAACAGCCTGTGGGTTGCACC
112. EU753384.2/China/Shandong/522-04T/SD/CHN/07/C4a/2007 (C4)	TTAAAACAGCCTGTGGGTTGCACC
113. HM003207.1/China/87-2008-Xi'an-Shaanxi/2008 (C4)	TTAAAACAGCCTGTGGGTTGCACC
114. EU753365.2/China/Shandong/518-03F/SD/CHN/07/C4a/2007 (C4)	TTAAAACAGCCTGTGGGTTGCACC
115. EU753407.2/China/Shandong/TC03F/SD/CHN/07/C4a/2007 (C4)	TTAAAACAGCCTGTGGGTTGCACC
116. GQ994988.1/China/Anhui/Anhui1-09-China/C4a/2009 (C4)	TTAAAACACCCTGTGGGTTGCACC
117. JQ708210.1/China/isolateTS/C4/2009 (C4)	TTAAAACAGCCTGTGGGTTGCACC
118. HQ611148.1/China/AH08/06/C4/2008 (C4)	TTAAAACAGCCTGTGGGTTGCACC
119. EU753398.2/China/Shandong/523-07T/SD/CHN/07/2007 (C4)	TTAAAACAGCCTGTGGGTTGCACC
120. EU753397.2/China/Shandong/523-05T/SD/CHN/07/C4a/2007 (C4)	TTAAAACAGCCTGTGGGTTGCACC
121. JX678882.1/China/Shandong/SD07-1/SD/CHN/2007/C4a/2007 (C4)	TTAAAAC-GCCTGTGGGTTGCACC
122. EU753375.2/China/Shandong/521-18S/SD/CHN/07/C4a/2007 (C4)	TTAAAACAGCCTGTGGGTTGCACC
123. HM002487.1/China/BJ303/2008 (C4)	TTAAAACAGCCTGTGGGTTGCACC
124. JX678878.1/China/Anhui/FY17.08-7/AH/CHN/2008/C4a/2008 (C4)	TTAAAAC-GCCTGTGGGTTGCACC
125. JX025561.1/China/FY7VP5/AH/CHN/2008 (C4)	TTAAAACAGCCTGTGGGTTGCACC
126. JQ316638.1/China/HQ09231463/2011 (C4)	TTAAAACAGCCTGTGGGTTGCACC
127. HQ426649.1/China/Anhui/2010FJLY008/2010/C4a/2010 (C4)	TTAAAACAG-CTGTGGGTTGCACC
128. GQ994991.1/China/Chongqing3-09-China/2009 (C4)	TTAAAACAGCCTGTGGGTTGCACC
129. HM053670.1/China/strain-BJ393/2009 (C4)	TTAAAACAGCCTGTGGGTTGCACC
130. FJ360544.1/China/GZ-08-01/C4/2008 (C4)	TTAAAACAG-CTGTGGGTTGTACC
131. HQ998852.1/China/Henan/EV71/Henan/106/2009/C4a/2009 (C4)	TTAAAACAGCCTGTGGGTTGCACC
132. JX244187.1/China/SDLY153/2010 (C4)	TTAAAACAGCCTGTGGGTTGCACC
133. GQ994990.1China/Chongqing2-09-China/C4/2009 (C4)	TTAAAACAGCCTGTGGGTTGCACC
134. EU864507.1/China/Zhejiang/EV71/Zhejiang08/2008/C4a/2008 (C4)	TTAAAACAGCCTGTGGGTTGCACC
135. FJ194965.1/China/EV71/GDSG/17/2008/C/2008 (C4)	TTAAAACAGCCTGTGGGTTGCACC
136. FJ607334.1/China/SHENZHEN/08/China/HFMD/2008/2008 (C4)	TTAAAACAG-CTGTGGGTTGCACC
137. GQ279370.1/China/SZ/HK08-6/2008 (C4)	TTAAAACAGCCTGTGGGTTGCACC
138. FJ607335.1/China/4/SHENZHEN/08/China/HFMD/2008/2008 (C4)	TTAAAACAG-CTGTGGGTTGCACC
139. JQ736684.2/China/SHAPHC695F/SH/CHN/10/C4a/2010 (C4)	TTAAAACAGCCTGTGGGTTGCACC
140. JQ074187.1/China/Jinan1002/2010 (C4)	TTAAAACAGCCTGTGGGTTGCACC
141. JQ074189.1/China/Jinan1005/2010 (C4)	TTAAAACAGCCTGTGGGTTGCACC
142. JQ074188.1/China/EV71/Jinan1004/2010 (C4)	TTAAAACAGCCTGTGGGTTGCACC
143. JF830007.1/China/Zhejiang/EV71/Ningbo.CHN/065/2010/C4a/2010 (C4)	TTAAAACAGCCTGTGGGTTGCACC

Sequence	10 · 20
Consensus	TTAAAACAGCCTGTGGGTTGCACC
	FL5F; Primer Bind
142. JQ074188.1/China/EV71/Jinan1004/2010 (C4)	TTAAAACAGCCTGTGGGTTGCACC
143. JF830007.1/China/Zhejiang/EV71/Ningbo.CHN/065/2010/C4a/2010 (C4)	TTAAAACAGCCTGTGGGTTGCACC
144. JX111893.1/China/GD-DG2011-9/C4/2011 (C4)	CAGCCTGTGGGTTGCACC
145. JX111891.1/China/GD-DG2011-5/C4/2011 (C4)	TTAAAACAGCCTGTGGGTTGCACC
146. JX111889.1/China/GD-DG2011-2/C4/2011 (C4)	TTAAAACAGCCTGTGGGTTGCACC
147. JX111888.1/China/GD-DG2011-1/C4/2011 (C4)	TAAAACAGCCTGTGGGTTGCACC
148. JX111892.1/China/GD-DG2011-8/C4/2011 (C4)	TTAAAACAGCCTGTGGGTTGCACC
149. JX111890.1/China/GD-DG2011-3/C4/2011 (C4)	TTAAAACAGCCTGTGGGTTGCACC
150. JQ806378.1/China/Jiangxi/35/Jingdezhen/China/HFMDSevere/2011/C4a/2011 (C4)	TTAAAACAGCCTGTGGGTTGCACC
151. JX017384.1/China/01011Y/2011 (C4)	TTAAAACAGCCTGTGGGTTGCACC
152. HQ891925.1/China/Shanghai-36-2009/C4/2009 (C4)	TTAAAACAGCCTGTGGGTTGCACC
153. JX244186.1/China/SDLY107/2010 (C4)	TTAAAACAGCCTGTGGGTTGCACC
154. HQ850973.1/China/003/XM/2010/2010 (C4)	
155. FJ360546.1/China/GZ-08-03/C4/2008 (C4)	TTAAAACAG-CTGTGGGTTGCACC
156. HQ891926.1/China/Shanghai-51-2009/C4/2009 (C4)	TTAAAACAGCCTGTGGGTTGCACC
157. GQ892830.1/China/GX/LZ (C4)	TTAAAACAGCCTGTGGGTTGTACC
158. JN020147.1/China/Henan/Luoyang/2011/C4a/2011 (C4)	TTAAAACAGCCTGTGGGTTGCACC
159. JQ517316.1/China/Henan/Kaifeng/2010/C4a/2010 (C4)	TTAAAACAGCCTGTGGGTTGCACC
160. JN052925.1/China/Henan/Nanyang/2011-China/2011/C4a/2011 (C4)	TTAAAACAGCCTGTGGGTTGCACC
161. HQ828086.1/China/Zhejiang/NBChina01/2010/C4a/2010 (C4)	TTAAAACAGCCTGTGGGTTGCACC
162. JX986739.1/China/Wuhan1143/HuB/CHN/2011/C4/2011 (C4)	TTAAAACAGCCTGTGGGTTGCACC
163. HQ407557.1/China/Liangning/LN009/2010/C4a/2010 (C4)	TTAAAACAGCCTGTGGGTTGCACC
164. JQ074190.1/China/Shandong/Jinan1006/2010/C4a/2010 (C4)	TTAAAACAGCCTGTGGGTTGCACC
165. JN001860.1/China/Zhejiang/NB/2010/01/C4a/2010 (C4)	TTAAAACAGCCTGTGGGTTGCACC
166. JQ804832.1/China/EV71/Hubei-XF/CHN/2010/C4/2010 (C4)	TTAAAACAGCCTGTGGGTTGCACC
167. JQ514785.1/China/BC08/C4a/2008 (C4)	TTAAAACAGCCTGTGGGTTGCACC
168. HM053669.1/China/BJ293/2008 (C4)	TTAAAACAGCCTGTGGGTTGCACC
169. HM002484.1/China/BJ67/2008 (C4)	TTAAAACAGCCTGTGGGTTGCACC
170. GU396280.1/China/Gansu/EV71/Lanzhou01/GS/CHN/09/C4a/2009 (C4)	TTAAAACAGCCTGTGGGTTGCACC
171. HQ400942.1/China/HZ08/Hangzhou/2008/C4/2008 (C4)	TTAAAACAGCCTGTGGGTTGCACC
172. HQ891929.1/China/Shanghai-118-2009/C4/2009 (C4)	TTAAAACAGCCTGTGGGTTGCACC
173. FJ607338.1/China/Shenzhen/605/SHENZHEN/08/China/HFMD/C4a/2008 (C4)	TTAAAACAG-CTGTGGGTTGCACC
174. JX986737.1/China/Wuhan1042/HuB/CHN/2011/C4/2011 (C4)	TAAAAACAGCCTGTGGGTTGCACC
175. HQ891927.1/China/Shanghai-64-2009/C4/2009 (C4)	TTAAAACAGCCTGTGGGTTGCACC
176. HQ891928.1/China/Shanghai-117-2009/C4/2009 (C4)	TTAAAACAGCCTGTGGGTTGCACC
177. HQ891924.1/China/Shanghai-28-2009/C4/2009 (C4)	TTAAAACAGCCTGTGGGTTGCACC
178. HQ891923.1/China/Shanghai/Shanghai-27-2009/C4a/2009 (C4)	TTTAAACAGCCTGTGGGTTGCACC
179. HM002489.1/China/Beijing/Beijing398/2009/C4a/2009 (C4)	TTAAAACAGCCTGTGGGTTGCACC
180. JN256063.1/China/RDcells/M186-1179F/HeN/CHN/2009/C4a (C4)	TTAAAACAGCCTGTGGGTTGCACC
181. JN256062.1/China/RDcells/M183-1176F/HeN/CHN/2009/C4a (C4)	TTAAAACAGCCTGTGGGTTGCACC
182. JN256064.1/China/Henan/RDcells/M188-1181F/HeN/CHN/2009/C4a/2009 (C4)	TTAAAACAGCCTGTGGGTTGCACC
183. JX678881.1/China/Henan/HeN09-17/HeN/CHN/2009/C4a/2009 (C4)	TTAAAAC-GCCTGTGGGTTGCACC
184. JX678880.1/China/Henan/HeN09-1/HeN/CHN/2009/C4a/2009 (C4)	TTAAAAC-GCCTGTGGGTTGCACC
185. EU812515.1/China/Anhui/FY23/2008/C4a/2008 (C4)	TTAAAACAGCCTGTGGGTTGCACC
186. GU459071.1/China/FY23-K14/C4/2008 (C4)	TTAAAACAG-CTGTGGGTTGCACC
187. GU459070.1/China/FY23-K12/C4/2008 (C4)	TTAAAACAGCCTGTGGGTTGCACC
188. JX244185.1/China/SDLY96/2009 (C4)	TTAAAACAGCCTGTGGGTTGCACC
189. JX244183.1/China/SDLY11/2009 (C4)	TTAAAACAGCCTGTGGGTTGCACC
190. JN256061.1/China/RDcells/G398-1037F/HeN/CHN/2009/C4a (C4)	TTAAAACAGCCTGTGGGTTGCACC
191. HQ825317.1/China/Shandong/EV71/JN200804/2008/C4a/2008 (C4)	TTAAAACAGCCTGTGGGTTGCACC
192. JF913464.1/China/Shandong/EV71/JN200803/2008/C4a/2008 (C4)	TTAAAACAGCCTGTGGGTTGCACC
193. JN256060.1/China/RDcellsG333-972F/HeN/CHN/2009/C4a (C4)	TTAAAACAGCCTGTGGGTTGCACC
194. JN256059.1/China/Henan/RDcells/G288-927F/HeN/CHN/2009/C4a/2009 (C4)	TTAAAACAGCCTGTGGGTTGCACC
195. JX678884.1/China/Shandong/SD09-21/SD/CHN/2009/C4a/2009 (C4)	TAAAAC-GCCTGTGGGTTGCACC
196. JX678883.1/China/Shandong/SD09-14/SD/CHN/2009/C4a/2009 (C4)	TTAAAACGCCTGTGGGTTGCACC
197. JQ319054.1/China/Beijing/BJ09/07/C4a/2009 (C4)	TTAAAACAG-CTGTGGGTTGCACC
198. JF820315.1/China/AH01/C4/2010 (C4)	
199. JF820314.1/China/BJ02/C4/2010 (C4)	
200. JQ708209.1/China/isolateBJ/C4/2010 (C4)	TTAAAACAG-CTGTGGGTTGCACC
201. JF820316.1/China/BJ01/C4/2010 (C4)	
202. JF820313.1/China/LCH02/C4/2010 (C4)	
203. FJ606449.1/China/BJ08-Z020-1/2008 (C4)	TTAAAACAGCCTGTGGGTTGCACC
204. GU198371.1/China/FY08-C30-P14/2008 (C4)	
205. GU198370.1/China/C2/FY08-C30/2008 (C4)	
206. GU198367.1/China/Anhui/FY08-C30-P2/2008/C4a/2008 (C4)	
207. GU198369.1/China/C1/FY08-C30-P11/2008 (C4)	
208. GU198368.1/China/C1/FY08-C30-P9/2008 (C4)	
209. FJ439769.1/China/Anhui/Fuyang-0805/2008/C4a/2008 (C4)	TTAAAACAGCCTGTGGGTTGCACC
210. EU703814.1/China/Anhui/2008/EV71/Fuyang.Anhui.P.R.C/17.08/3/2008/C4a/2008 (C4)	TTAAAACAGCCTGTGGGTTGCACC
211. EU703812.1/China/Anhui/EV71/Fuyang.Anhui.P.R.C/17.08/1/2008/C4a/2008 (C4)	TTAAAACAGCCTGTGGGTTGCACC
212. JX678875.1/China/Anhui/FY17.08-4/AH/CHN/2008/C4a/2008 (C4)	TTAAAA-AGCCTGTGGGTTGCACC
213. FJ158601.1/China/DTID/ZJU-74/2008 (C4)	TTAAAACAGCCTGTGGGTTGCACC
214. FJ158600.1/China/Zhejiang/DTID/ZJU-62/2008/C4a/2008 (C4)	TTAAAACAGCCTGTGGGTTGCACC

Sequence	10 … 20
Consensus	TTAAAACAGCCTGTGGGTTGCACC
	FL5F; Primer Bind
212. JX678875.1/China/Anhui/FY17.08-4/AH/CHN/2008/C4a/2008 (C4)	TTAAAA-AGCCTGTGGGTTGCACC
213. FJ158601.1/China/DTID/ZJU-74/2008 (C4)	TTAAAACAGCCTGTGGGTTGCACC
214. FJ158600.1/China/Zhejiang/DTID/ZJU-62/2008/C4a/2008 (C4)	TTAAAACAGCCTGTGGGTTGCACC
215. FJ606448.1/China/BJ08-Z011-4/C4/2008 (C4)	TTAAAACAGCCTGTGGGTTGCACC
216. EU703813.1/China/Anhui/EV71/Fuyang.Anhui.P.R.C/17.08/2/2008/C4a/2008 (C4)	TTAAAACAGCCTGTGGGTTGCACC
217. JX678879.1/China/Anhui/FY17.08-8/AH/CHN/2008/C4a/2008 (C4)	TTAAAAC-GCCTGTGGGTTGCACC
218. JX678877.1/China/Anhui/FY17.08-6/AH/CHN/2008/C4a/2008 (C4)	TTAAAAC-GCCTGTGGGTTGCACC
219. JX678876.1/China/Anhui/FY17.08-5/AH/CHN/2008/C4a/2008 (C4)	TTAAAAC-GCCTGTGGGTTGCACC
220. JF799986.1/China/LabstrainGuangdong/2009 (C4)	TTAAAACAGCCTGTGGGTTGCACC
221. JF738002.1/Thailand/THA-EV71-044/C4/2009 (C4)	
222. FJ607337.1/China/Shenzhen/121/SHENZHEN/08/China/HFMD-Fatal/2008/C4a/2008 (C4)	TTAAAACAG-CTGTGGGTTGCACC
223. FJ606450.1/China/BJ08-Z025-5/2008 (C4)	TTTAAACAGCCTGTGGGTTGCACC
224. HM622391.1/Taiwan/2008-00643/C2-like/2008 (C2)	CTGTGGGTTGTTCC
225. JQ280307.1/Taiwan/3149/C2/2008 (C2)	TTAAAACAGCCTGTGGGTTGTTCC
226. HM622392.1/Taiwan/2008-07776/C2-like/2008 (C2)	CTGTGGGTTGTTCC
227. EU527983.1/Taiwan/2007-07364/2007/C5/2007 (C5)	GTACC
228. EF063152.1/Taiwan/E2005125-TW (C5)	TTAAAACAGCCTGTGGGTTGTACC
229. DQ341355.1/SouthKorea/06-KOR-00/C3/2000 (C3)	TTAAAACAGCCTGTGGGTTGTACC
230. DQ341356.1/SouthKorea/03-KOR-00/C3/2000 (C3)	TTAAAACAGCCTGTGGGTTGTGCC
231. AF119795.2/Taiwan/TW/2272/98/C/1998 (UNKNOWN)	TTAAAACAG-CTGTGGGTTGCACC
232. AF119796.3/Taiwan/TW/2086/98/C2/1998 (C2)	TTAAAACAG-CTGTGGGTTGCACC
233. DQ060149.1/Taiwan/pinf7-54A/C2/2005 (C2)	TTAAAACAGCCTGTGGGTTGCACC
234. AF304457.1/Taiwan/5746/98/C2/1998 (C2)	TTAAAACAGCCTGTGGGTTGCACC
235. AF176044.1/Taiwan/1245a/98/tw (C2)	TTAAAACAGCCTGTGGGTTGCACC
236. JN544418.1/China/4643-TW98/C2/1998 (C2)	TTAAAACAGCCTGTGGGTTGCACC
237. AF304458.1/Taiwan/Tainan/4643/98/C2 (C2)	TTAAAACAGCCTGTGGGTTGCACC
238. AF304459.1/Taiwan/Tainan/6092/98/C2 (C2)	TTAAAACAGCCTGTGGGTTGCACC
239. N544419.1/China/mousestrain/MP4/C2/2004 (C2)	TTAAAACAGCCTGTGGGTTGCACC
240. AF136379.1/NCKU9822 (C2)	TTAAAACAGCCTGTGGGTTGCACC
241. AB550333.1/Japan/Labstrain/1095-LPS1/C2/1997 (C2)	TTAAAACAGCCTGTGGGTTGCACC
242. AB550332.1/Japan/1095-org/C2/1997 (C2)	TTAAAACAGCCTGTGGGTTGCACC
243. JN992283.1/Australia/0964/SYD/98 (C2)	TTAAAACAGCCTGTGGGTTGTACC
244. JX025559.1/Australia/Labstrain/LAZ60-TR.C2/2010 (C2)	TTAAAACAGCCTGTGGGTTGCACC
245. DQ381846.1/Australia/6F/AUS/6/99/C2/1999 (C2)	TTAAAACAGCCTGTGGGTTGCACC
246. DQ341357.1/Australia/7F-AUS-6-99 (C2)	TTAAAACAGCCTGTGGGTTGCACC
247. AB575939.1/Netherlands/365/C2/2000 (C2)	
248. HQ647173.1/Canada/EV003-07/2006 (C2)	TTAAAACAG-CTGTGGGTTGCACC
249. HQ647168.1/Canada/EV92-07/2007 (C2)	TTAAAACAG-CTGTGGGTTGCACC
250. HQ647174.1/Cana/EV123-07/2007 (C2)	TTAAAACAG-CTGTGGGTTGCACC
251. HQ647170.1/Canada/EV124-07/2007 (C2)	TTAAAACAG-CTGTGGGTTGCACC
252. HQ647176.1/Canada/EV049-07/2007 (C2)	TTAAAACAGCT--TGGGTTGCACC
253. HQ647169.1/Canada/EV090-07/2007 (C2)	ACC
254. HQ647178.1/Canada/EV082-07/2007 (C2)	TTAAAACAG-CTGTGGGTTGCACC
255. HQ647177.1/Canada/EV073-07/2007 (C2)	TTAAAACAG-CTGTGGGTTGCACC
256. HQ647175.1/Canada/EV053-07/2007 (C2)	TTAAAACAG-CTGTGGGTTGCACC
257. HQ647167.1/Canda/EV054-07/2007 (C2)	TTAAAACAG-CTGTGGGTTGCACC
258. FJ172159.1/Singapore/NUH0075/SIN/08/2008 (C2)	
259. AB575942.1/Netherlands/3692/C2/2007 (C2)	
260. AB575941.1/Netherlands/2485/C2/2007 (C2)	
261. JN835312.1/France//MRS/09/3663/C3/2009 (C3)	TTAAAACAGGCTGTGGGTTGTACC
262. AB575948.1/Netherlands/10118/C2/2010 (C2)	
263. AB575938.1/Netherlands/10098/C1/2010 (C1)	
264. JF738000.1/Thailand/THA-EV71-002/C1/2009 (C1)	
265. EU414335.2/Switzerland/PLday1-CH-06/C1 (C1)	
266. EU414334.2/Switzerland/PLday4-CH-06/C1 (C1)	
267. EU414333.2/Switzerland/CSF-CH-06/C1 (C1)	
268. EU414331.2/Switzerland/LR-CH-06/C1 (C1)	
269. DQ341360.1/Malaysia/J115-MAL-01 (C1)	TTAAAACAGCCTGTGGGTTGTACC
270. DQ341358.1/Malaysia/S40221-SAR-00/C1/2000 (C1)	TTAAAACAGCCTGTGGGTTGTACC
271. AB550341.1/Malaysia/Labstrain/KED005-LPS2/C1/1997 (C1)	TTAAAACAGCCTGTGGGTTGTACC
272. AB550340.1/Malaysia/KED005-org/C1/1997 (C1)	TTAAAACAGCCTGTGGGTTGTACC
273. DQ452074.1/Norway/804/NO/03/C1/2003 (C1)	TTAAAACAGCCTGTGGGTTGCACC
274. DQ341361.1/Australia/1M-AUS-12-00/C1 (C1)	TTAAAACAGCCTGTGGGTTGTACC
275. AB575937.1/Netherlands/1416/C1/2001 (C1)	
276. DQ341359.1/Malaysia/S10862-SAR-98/C1/1998 (C1)	TTAAAACAGCCTGTGGGTTGCACC
277. HQ647172.1/Canada/EV063-04/1994 (C1)	TTAAAACAG-CTGTGGGTTGCACC
278. AB575936.1/Netherlands/9612/C1/1991 (C1)	
279. AB575935.1/Netherlands/480/C1/1991 (C1)	

	3,190 / 3,200
Consensus	TCCAAGTCCAAGTACCCTTTAGT
	FL9R
1. GU434678.1/China/Hubei/EV71-Hubei-09-China/B5/2009 (B5)	TCAAAGTCAAAATATCCCTTAGT
2. AB204853.1/BrCr-ts/A (A)	TCACAGTCAAAATATCCCTTAGT
3. AB575911.1/Netherlands/10076/B0/1966 (B0)	TCGAAATCCAAGTACCCTCTAGT
4. AB575912.1/Netherlands/10857/B0/1966 (B0)	TCGAAATCCAAGTACCCTCTAGT
5. DQ341354.1/Singapore/3799-SIN-98/B3/1998 (B3)	TCAAAGTCCAAGTACCCTCTGGT
6. DQ341368.1/Malaysia/MY104-9-SAR-97/B3 (B3)	TCAAAGTCCAAATACCCTCTGGT
7. DQ341367.1/Malaysia/MY821-3-SAR-97/B3/1997 (B3)	TCAAAGTCCAAGTACCCTCTGGT
8. AB550335.1/Malaysia/Labstrain/SK-EV006-LPS1/B3/1997 (B3)	TTAAAGTCCAAGTACCCTCTGGT
9. AB550334.1/Malaysia/SK-EV006-org/B3/1997 (B3)	TTAAAGTCCAAGTACCCTCTGGT
10. AB469182.1/Malaysia/SK-EV006/Malaysia/97 (B3)	TTAAAGTCCAAGTACCCTCTGGT
11. JQ950555.1/Australia26M/AUS/4/99/GuaR1/B3/2010 (B3)	TCAAAGTCCAAGTACCCTCTGGT
12. EU364841.1/Australia/26M/AUS/4/99/B3/1999 (B3)	TCAAAGTCCAAGTACCCTCTGGT
13. EF373575.1/Taiwan/E2002042-TW-CDC (B4)	TCAAAGTCCAAGTACCCTTTAGT
14. FJ357377.1/Taiwan/S0296-TW00/B4/2000 (B4)	TCAAAGTCCAAGTACCCCTTAGT
15. FJ357376.1/Taiwan/S0318-TW01/B4/2001 (B4)	TCAAAGTCCAAGTACCCTTTAGT
16. DQ341366.1/Malaysia/SB2864-SAR-00/B4/2000 (B4)	TCAAAGTCCAAGTACCCTTTGGT
17. DQ341365.1/Malaysia/PP37-MAL-01/B4/2001 (B4)	TCAAAGTCCAAGTACCCTTTGGT
18. AF316321.2/Singapore/5865/sin/000009/B4 (B4)	TCAAAGTCCAAGTATCCTTTGGT
19. AF352027.1/Singapore/5666/sin/002209/B4 (B4)	TCAAAGTCCAAGTATCCTTTGGT
20. FJ357375.1/Taiwan/N7008-TW99/B4/1999 (B4)	TCAAAGTCCAAGTACCCTTTGGT
21. AB550337.1/Japan/Labstrain/C7/Osaka-LPS1/B4/1997 (B4)	TTAAAGTCCAAGTACCCTTTGGT
22. AB550336.1/Japan/C7/Osaka-org/B4/1997 (B4)	TTAAAGTCCAAGTACCCTTTGGT
23. FJ461781.1/Singapore/NUH0083/SIN/08/B5/2008 (B5)	TCAAAGTCCAAGTACCCTTTGGT
24. JF738001.1/Thailand/THA-EV71-019/B5/2009 (B5)	TCAAAGTCCAAGTACCCTTTGGT
25. DQ341364.1/Singapore/5511-SIN-00 (B5)	TCAAAGTCCAAGTACCCTTTGGT
26. JN992282.1/Brunei/BRU/2006/35334 (B5)	TCAAAGTCCAAGTACCCTTTGGT
27. FJ357378.1/Taiwan/N2838-TW03/B5/2003 (B5)	TCAAAGTCCAAGTACCCTTTGGT
28. DQ341363.1/Malaysia/S19841-SAR-03/B5/2003 (B5)	TCAAAGTCCAAGTACCCTTTGGT
29. DQ341362.1/Malaysia/SB12736-SAR-03/B4/2003 (B4)	TCAAAGTCCAAGTACCCTTTGGT
30. JN964686.1/China/Fujian/EV71/Xiamen/2009/B5/2009 (B5)	TCAAAGTCCAAGTACCCTTTGGT
31. GQ231942.1/Taiwan/TW/96016/08/B5 (B5)	TCAAAGTCCAAGTATCCTTTGGT
32. GQ231925.1/Taiwan/TW/1101/08/B5 (B5)	TCAAAGTCCAAGTATCCTTTGGT
33. GQ231934.1/Taiwan/TW/70811/08/B5 (B5)	TCAAAGTCCAAGTATCCTTTGGT
34. GQ231943.1/Taiwan/TW/96022/08/B5 (B5)	TCAAAGTCCAAGTATCCTTTGGT
35. GQ231935.1/Taiwan//TW/70886/08/B5 (B5)	TCAAAGTCCAAGTATCCTTTGGT
36. GQ231941.1/Taiwan/TW/96002/08/B5 (B5)	TCAAAGTCCAAGTATCCTTTGGT
37. EU527985.1/Taiwan/2007-08747/2007/B5/2007 (B5)	TCAAAGTCCAAGTATCCTTTGGT
38. FJ357385.1/Taiwan/M0380-TW08/B5/2008 (B5)	TCAAAGTCCAAGTACCCTTTGGT
39. HM622390.1/Taiwan/2009-03531/B5/2009 (B5)	TCAAAGTCCAAGTACCCTTTGGT
40. GQ231936.1/Taiwan/TW/70902/08/B5 (B5)	TCAAAGTCCAAGTACCCTTTGGT
41. AB575927.1/Netherlands/11316/B2/1986 (B2)	TTGAAGTCCAAGTATCCCTTAGT
42. AB575928.1/Netherlands/11590/B2/1986 (B2)	TCGAAGTCCAAGTATCCCTTAGT
43. AB575923.1/Netherlands/20233/B2/1983 (B2)	TCGAAGTCTAAGTATCCCTTAGT
44. U22522.1/USA/MS/7423/87/B2/1987 (B2)	TCGAAGTCCAAGTATCCCTTAGT
45. FJ357384.1/Taiwan/266-TW86/B1/1986 (B1)	TCGAAATCCAAGTATCCTTTAGT
46. FJ357383.1/Taiwan/252-TW86/B1/1986 (B1)	TCGAAATCCAAGTATCCTTTAGT
47. FJ357380.1/Taiwan/237-TW86/B1/1986 (B1)	TCGAAATCCAAGTATCCCTTAGT
48. FJ357379.1/Taiwan/236-TW86/B1/1986 (B1)	TCGAAATCCAAGTATCCTTTAGT
49. FJ357382.1/Taiwan/238-TW86/B1/1986 (B1)	TCGAAATCCAAGTATCCTTTAGT
50. FJ357381.1/Taiwan/244-TW86/B1/1986 (B1)	TCGAAATCCAAGTATCCTTTAGT
51. AB575914.1/Netherlands/17000/B1/1971 (B1)	TCGAAATCCAAGTACCCCTTAGT
52. AB575913.1/Netherlands/11977/B1/1971 (B1)	TCGAAATCCAAGTACCCCTTAGT
53. AB575918.1/Netherlands/20574/B1/1978 (B1)	TCGAAATCCAAGTATCCCTTAGT
54. AB575917.1/Netherlands/10196/B1/1977 (B1)	TCGAAGTCCAAGTATCCCTTAGT
55. HQ189392.1/Hungary/HUN/1978/1978 (B1)	TCGAAATCCAAGTATCCCTTAGT
56. AB575916.1/Netherlands/16173/B1/1976 (B1)	TCGAAATCCAAGTATCCCTTAGT
57. AB575915.1/Netherlands/9443/B1/1974 (B1)	TCGAAATCCAAGTACCCCTTAGT
58. AB482183.1/Japan/Nagoya/B1/1973 (B1)	TTGAAATCCAAGTATCCCTTAGT
59. HQ423143.1/China/Yunnan/KM186/09/2009/C4a/2009 (C4)	TCCAAGTCCAAGTACCCTTTGGT
60. HQ423142.1/China/KMM/09/2009 (C4)	TCCAAGTCCAAGTACCCTTTGGT
61. JQ742001.1/China/Guangxi/AFP2001064/EV71/GX/CHN/2001/C4b/2001 (C4)	TCCAAGTCCAAGTACCCTTTAGT
62. EU131776.1/Taiwan/N3340-TW-02/C4b/2002 (C4)	TCCAAGTCCAAGTACCCTTTAGT
63. JX678885.1/China/Shanghai/SH-17/SH/CHN/2002/C4b/2002 (C4)	TCCAAGTCCAAGTACCCTTTAGT
64. JQ742002.1/China/AFP2001071/EV71/GX/CHN/2001/C4b/2001 (C4)	TCCAAGTCCAAGTACCCTTTAGT
65. GU350629.1/China/AnHui/Fuyang-0805a (C4)	TCCAAGTCCAAGTACCCTTTAGT
66. FJ194964.1/China/Guangdong/EV71/GDFS/3/2008/C4b/2008 (C4)	TCCAAGTCCAAGTACCCTTTGGT
67. GQ994989.1/China/Chongqing/Chongqing1-09-China/2009/C4b/2009 (C4)	TCCAAGTCCAAGTACCCTTTAGT
68. HM807310.1/Taiwan/cmuh-050530-5/2005 (C4)	TCCAAGTCCAAGTACCCTTTGGT
69. AY465356.1/China/Shenzhen/SHZH03/C4b/2003 (C4)	TCCAAATCCAAGTACCCTTTGGT
70. JX678874.1/China/Chongqing/CQ03-1/CQ/CHN/2003/C4b/2003 (C4)	TCCAAGTCCAAGTACCCTTTAGT
71. HQ647171.1/Canada/EV141-06/2006/C4a/2006 (C4)	TCCAAGTCCAAGTACCCTTTAGT
72. HQ647180.1/Canada/EV034-06/2006 (C4)	TCCAAGTCCAAGTACCCTTTAGT

	3,190 / 3,200 (FL9R)
Consensus	TCCAAGTCCAAGTACCCTTTAGT
71. HQ647171.1/Canada/EV141-06/2006/C4a/2006 (C4)	TCCAAGTCCAAGTACCCTTTAGT
72. HQ647180.1/Canada/EV034-06/2006 (C4)	TCCAAGTCCAAGTACCCTTTAGT
73. HQ647179.1/Canada/EV144-06/2006 (C4)	TCCAAGTCCAAGTACCCTTTAGT
74. JX678886.1/China/Shanghai/SH-6/SH/CHN/2002/C4b/2002 (C4)	TCCAAGTCCAAGTACCCTTTAGT
75. AB550339.1/Japan/Labstrain/75-Yamagata-LPS1/C4/2003 (C4)	TCCAAGTCCAAGTACCCTTTAGT
76. AB550338.1/Japan/75-Yamagata-org/C4/2003 (C4)	TCCAAGTCCAAGTACCCTTTAGT
77. DQ133459.1/Taiwan/1235/C4/2004 (C4)	TCCAAGTCCAAGTACCCTTTAGT
78. DQ133458.1/Taiwan/984/C4/2004 (C4)	TCCAAGTCCAAGTACCCTTTGGT
79. FJ357373.1/Taiwan/S0584-TW04/C4/2004 (C4)	TCCAAGTCCAAGTACCCTTTGGT
80. EF373576.1/Taiwan/E2004104-TW-CDC (C4)	TCCAAGTCCAAGTACCCTTTGGT
81. GQ231938.1/TaiwanTW/71552/05 (C4)	TCCAAGTCCAAGTACCCTTTGGT
82. GQ231926.1/Taiwan/TW/1956/05/C4a/2005 (C4)	TCCAAGTCCAAGTACCCTTTGGT
83. GQ231940.1/Taiwan/TW/72232/04/C4 (C4)	TCCAAGTCCAAGTACCCTTTGGT
84. GQ231937.1/Taiwan/TW/71428/05/C4 (C4)	TCCAAGTCCAAGTACCCTTTGGT
85. GQ231939.1/Taiwan/TW/71595/04/C4 (C4)	TCCAAGTCCAAGTACCCTTTGGT
86. GQ231929.1/Taiwan/TW/2728/04/C4a/2004 (C4)	TCCAAGTCCAAGTACCCTTTGGT
87. FJ357374.1/Taiwan/N2121-TW05/C4/2005 (C4)	TCCAAGTCCAAGTACCCTTTGGT
88. GQ231931.1/Taiwan/TW/2824/04/C4 (C4)	TCCAAGTCCAAGTACCCTTTGGT
89. GQ231928.1/Taiwan/TW/2639/04/C4 (C4)	TCCAAGTCCAAGTACCCTTTGGT
90. GQ231927.1/Taiwan/TW/2429/04/C4 (C4)	TCCAAGTCCAAGTACCCTTTGGT
91. GQ231930.1/Taiwan/TW/2815/04/C4 (C4)	TCCAAGTCCAAGTACCCTTTGGT
92. JQ965759.1/Vietnam/540V/VNM/05/C4/2005 (C4)	TCCAAGTCCAAGTACCCTTTGGT
93. GQ231932.1/Taiwan/TW/2871/04/C4 (C4)	TCCAAGTCCAAGTACCCTTTGGT
94. GQ994992.1/China/Henan/Henan2-09-China/2009/C4a/2009 (C4)	TCCAAGTCCAAGTACCCTTTAGT
95. GQ279369.1/China/SZ/HK08-5/2008 (C4)	TCCAAATCCAAGTATCCTTTAGT
96. JX244184.1/China/SDLY48/C/2009 (C4)	TCCAAGTCCAAATATCCTTTAGT
97. FJ606447.1/China/Beijing/BJ08-Z004-3/2008/C4a/2008 (C4)	TCCAAGTCCAAGTACCCTTTAGT
98. JQ681218.1/China/Henan/H8-1/C4a/2008 (C4)	TCCAAGTCCAAGTACCCTTTAGT
99. GU196833.1/China/Henan1-09-China/2009 (C4)	TCCAAGTCCAAGTACCCTTTAGT
100. HQ129932.1/China/BJ06-SJS06/06/C4/2006 (C4)	TCCAAGTCCAAGTACCCTTTAGT
101. FJ607336.1/China/28/SHENZHEN/08/China/HFMD/2008/2008 (C4)	TCCAAGTCCAAGTACCCTTTAGT
102. FJ828519.1/China/BJ08/C4/2008 (C4)	TCCAAGTCCAAGTACCCTTTAGT
103. FJ360545.1/China/GZ-08-02/C4/2008 (C4)	TCCAAATCCAAGTACCCTTTGGT
104. JX244182.1/China/SDLY1/2009 (C4)	TCCAAGTCCAAGTACCCCTTAGT
105. JF820312.1/China/LCH01/C4/2010 (C4)	TCCAAGTCCAAGTACCCTTTAGT
106. HM002488.1/China/BJ366/2009 (C4)	TCCAAGTCCAAGTACCCTTTAGT
107. GQ231933.1/Taiwan/TW/70516/08/C4a/2008 (C4)	TCCAAGTCCAAGTACCCTTTAGT
108. KC109780.1/China/202/Jingdezhen/China/HFMDSevere/2011/C4/2011 (C4)	TCCAAGTCCAAGTACCCATTAGT
109. HM002485.1/China/BJ972008 (C4)	TCCAAGTCCAAGTATCCTTTAGT
110. GU366191.1/China/Henan/Henan10-08-China/2008/C4a/2008 (C4)	TCCAAGTCCAAGTACCCTTTAGT
111. FJ713137.1/Shanghai/Shanghai-036-2009/C4a/2009 (C4)	TCCAAGTCCAAGTATCCTTTAGT
112. EU753384.2/China/Shandong/522-04T/SD/CHN/07/C4a/2007 (C4)	TCCAAGTCCAAGTACCCTTTAGT
113. HM003207.1/China/87-2008-Xi'an-Shaanxi/2008 (C4)	TCCAAGTCCAAGTACCCTTTAGT
114. EU753365.2/China/Shandong/518-03F/SD/CHN/07/C4a/2007 (C4)	TCCAAGTCCAAGTACCCTTTAGT
115. EU753407.2/China/Shandong/TC03F/SD/CHN/07/C4a/2007 (C4)	TCCAAGTCCAAGTACCCTTTAGT
116. GQ994988.1/China/Anhui/Anhui1-09-China/C4a/2009 (C4)	TCCAAGTCCAAGTACCCTTTAGT
117. JQ708210.1/China/isolateTS/C4/2009 (C4)	TCCAAGTCCAAGTACCCTTTAGT
118. HQ611148.1/China/AH08/06/C4/2008 (C4)	TCCAAGTCCAAGTACCCTTTAGT
119. EU753398.2/China/Shandong/523-07T/SD/CHN/07/2007 (C4)	TCCAAGTCCAAGTACCCTTTAGT
120. EU753397.2/China/Shandong/523-05T/SD/CHN/07/C4a/2007 (C4)	TCCAAGTCCAAGTACCCTTTAGT
121. JX678882.1/China/Shandong/SD07-1/SD/CHN/2007/C4a/2007 (C4)	TCCAAGTCCAAGTACCCTTTAGT
122. EU753375.2/China/Shandong/521-18S/SD/CHN/07/C4a/2007 (C4)	TCCAAGTCCAAGTACCCTTTAGT
123. HM002487.1/China/BJ303/2008 (C4)	TCCAAGTCCAAGTACCCTTTAGT
124. JX678878.1/China/Anhui/FY17.08-7/AH/CHN/2008/C4a/2008 (C4)	TCCAAGTCCAAGTACCCTTTAGT
125. JX025561.1/China/FY7VP5/AH/CHN/2008 (C4)	TCCAAGTCCAAGTACCCTTTAGT
126. JQ316638.1/China/HQ09231463/2011 (C4)	TCCAAGTCCAAGTACCCTTTAGT
127. HQ426649.1/China/Anhui/2010FJLY008/2010/C4a/2010 (C4)	TCCAAGTCCAAGTACCCTTTGGT
128. GQ994991.1/China/Chongqing3-09-China/2009 (C4)	TCCAAGTCTAAGTACCCTTTAGT
129. HM053670.1/China/strain-BJ393/2009 (C4)	TCCAAGTCCAAGTATCCTTTAGT
130. FJ360544.1/China/GZ-08-01/C4/2008 (C4)	TCCAAGTCCAAGTACCCTTTAGT
131. HQ998852.1/China/Henan/EV71/Henan/106/2009/C4a/2009 (C4)	TCCAAGTCCAAGTACCCTTTAGT
132. JX244187.1/China/SDLY153/2010 (C4)	TCCAAGTCCAAGTACCCTTTAGT
133. GQ994990.1China/Chongqing2-09-China/C4/2009 (C4)	TCCAAGTCCAAGTACCCTCTAGT
134. EU864507.1/China/Zhejiang/EV71/Zhejiang08/2008/C4a/2008 (C4)	TCCAAGTCCAAGTACCCTTTAGT
135. FJ194965.1/China/EV71/GDSG/17/2008/C/2008 (C4)	TCCAAGTCCAAGTACCCTTTAGT
136. FJ607334.1/China/SHENZHEN/08/China/HFMD/2008/2008 (C4)	TCCAAGTCCAAGTACCCTTTAGT
137. GQ279370.1/China/SZ/HK08-6/2008 (C4)	TCCAAGTCCAAGTACCCTTTAGT
138. FJ607335.1/China/4/SHENZHEN/08/China/HFMD/2008/2008 (C4)	TCCAAGTCCAAGTACCCTTTAGT
139. JQ736684.2/China/SHAPHC695F/SH/CHN/10/C4a/2010 (C4)	TCCAAGTCCAAGTACCCTTTAGT
140. JQ074187.1/China/Jinan1002/2010 (C4)	TCCAAGTCCAAGTACCCTTTAGT
141. JQ074189.1/China/Jinan1005/2010 (C4)	TCCAAGTCCAAGTACCCTTTAGT
142. JQ074188.1/China/EV71/Jinan1004/2010 (C4)	TCCAAGTCCAAGTACCCTTTAGT
143. JF830007.1/China/Zhejiang/EV71/Ningbo.CHN/065/2010/C4a/2010 (C4)	TCCAAGTCCAAGTACCCATTAGT

	3,190 / 3,200
Consensus	TCCAAGTCCAAGTACCCTTTAGT
	FL9R
143. JF830007.1/China/Zhejiang/EV71/Ningbo.CHN/065/2010/C4a/2010 (C4)	TCCAAGTCCAAGTACCCATTAGT
144. JX111893.1/China/GD-DG2011-9/C4/2011 (C4)	TCCAAGTCCAAGTACCCTTTAGT
145. JX111891.1/China/GD-DG2011-5/C4/2011 (C4)	TCCAAGTCCAAGTACCCTTTAGT
146. JX111889.1/China/GD-DG2011-2/C4/2011 (C4)	TCCAAGTCCAAGTACCCTTTAGT
147. JX111888.1/China/GD-DG2011-1/C4/2011 (C4)	TCCAAGTCCAAGTACCCTTTAGT
148. JX111892.1/China/GD-DG2011-8/C4/2011 (C4)	TCCAAGTCCAAGTACCCTTTAGT
149. JX111890.1/China/GD-DG2011-3/C4/2011 (C4)	TCCAAGTCCAAGTACCCTTTAGT
150. JQ806378.1/China/Jiangxi/35/Jingdezhen/China/HFMDSevere/2011/C4a/2011 (C4)	TCCAAGTCCAAGTACCCTTTAGT
151. JX017384.1/China/01011Y/2011 (C4)	TCCAAGTCCAAGTACCCTTTAGT
152. HQ891925.1/China/Shanghai-36-2009/C4/2009 (C4)	TCCAAGTCCAAGTACCCTTTAGT
153. JX244186.1/China/SDLY107/2010 (C4)	TCCAAGTCCAAGTACCCTTTAGT
154. HQ850973.1/China/003/XM/2010/2010 (C4)	TCCAAGTCCAAGTACCCTTTAGT
155. FJ360546.1/China/GZ-08-03/C4/2008 (C4)	TCCAAGTCCAAGTACCCTTTAGT
156. HQ891926.1/China/Shanghai-51-2009/C4/2009 (C4)	TCCAAGTCCAAGTACCCTTTAGT
157. GQ892830.1/China/GX/LZ (C4)	TCCAAGTCCAAGTACCCTTTAGT
158. JN020147.1/China/Henan/Luoyang/2011/C4a/2011 (C4)	TCCAAGTCCAAGTACCCTTTAGT
159. JQ517316.1/China/Henan/Kaifeng/2010/C4a/2010 (C4)	TCCAAGTCCAAGTACCCTTTAGT
160. JN052925.1/China/Henan/Nanyang/2011-China/2011/C4a/2011 (C4)	TCCAAGTCCAAGTACCCTTTAGT
161. HQ828086.1/China/Zhejiang/NBChina01/2010/C4a/2010 (C4)	TCCAAGTCCAAGTACCCTTTAGT
162. JX986739.1/China/Wuhan1143/HuB/CHN/2011/C4/2011 (C4)	TCCAAGTCCAAGTACCCTTTAGT
163. HQ407557.1/China/Liangning/LN009/2010/C4a/2010 (C4)	TCCAAGTCTAAGTACCCTTTAGT
164. JQ074190.1/China/Shandong/Jinan1006/2010/C4a/2010 (C4)	TCCAAGTCCAAGTACCCTTTAGT
165. JN001860.1/China/Zhejiang/NB/2010/01/C4a/2010 (C4)	TCCAAGTCCAAGTACCCTTTAGT
166. JQ804832.1/China/EV71/Hubei-XF/CHN/2010/C4/2010 (C4)	TCCAAGTCCAAGTACCCTTTAGT
167. JQ514785.1/China/BC08/C4a/2008 (C4)	TCCAAGTCCAAGTACCCTTTAGT
168. HM053669.1/China/BJ293/2008 (C4)	TCCAAGTCCAAGTACCCTTTAGT
169. HM002484.1/China/BJ67/2008 (C4)	TCCAAGTCCAAGTACCCTTTAGT
170. GU396280.1/China/Gansu/EV71/Lanzhou01/GS/CHN/09/C4a/2009 (C4)	TCCAAGTCCAAGTACCCTTTAGT
171. HQ400942.1/China/HZ08/Hangzhou/2008/C4/2008 (C4)	TCCAAGTCCAAGTACCCTTTAGT
172. HQ891929.1/China/Shanghai-118-2009/C4/2009 (C4)	TCCAAGTCCAAGTACCCTTTAGT
173. FJ607338.1/China/Shenzhen/605/SHENZHEN/08/China/HFMD/C4a/2008 (C4)	TCCAAGTCCAAGTACCCTTTAGT
174. JX986737.1/China/Wuhan1042/HuB/CHN/2011/C4/2011 (C4)	TCCAAGTCCAAGTACCCTTTAGT
175. HQ891927.1/China/Shanghai-64-2009/C4/2009 (C4)	TCCAAGTCCAAGTACCCTTTAGT
176. HQ891928.1/China/Shanghai-117-2009/C4/2009 (C4)	TCCAAGTCCAAGTACCCTTTAGT
177. HQ891924.1/China/Shanghai-28-2009/C4/2009 (C4)	TCCAAGTCCAAGTACCCTTTAGT
178. HQ891923.1/China/Shanghai/Shanghai-27-2009/C4a/2009 (C4)	TCCAAGTCCAAGTACCCTTTAGT
179. HM002489.1/China/Beijing/Beijing398/2009/C4a/2009 (C4)	TCCAAGTCCAAGTACCCTTTAGT
180. JN256063.1/China/RDcells/M186-1179F/HeN/CHN/2009/C4a (C4)	TCCAAGTCCAAGTACCCTTTAGT
181. JN256062.1/China/RDcells/M183-1176F/HeN/CHN/2009/C4a (C4)	TCCAAGTCCAAGTACCCTTTAGT
182. JN256064.1/China/Henan/RDcells/M188-1181F/HeN/CHN/2009/C4a/2009 (C4)	TCCAAGTCCAAGTACCCTTTAGT
183. JX678881.1/China/Henan/HeN09-17/HeN/CHN/2009/C4a/2009 (C4)	TCCAAGTCCAAGTATCCTTTAGT
184. JX678880.1/China/Henan/HeN09-1/HeN/CHN/2009/C4a/2009 (C4)	TCCAAGTCCAAGTACCCTTTAGT
185. EU812515.1/China/Anhui/FY23/2008/C4a/2008 (C4)	TCCAAGTCCAAGTACCCTTTAGT
186. GU459071.1/China/FY23-K14/C4/2008 (C4)	TCCAAGTCCAAGTACCCTTTAGT
187. GU459070.1/China/FY23-K12/C4/2008 (C4)	TCCAAGTCCAAGTACCCTTTAGT
188. JX244185.1/China/SDLY96/2009 (C4)	TCCAAGTCCAAGTACCCTTTAGT
189. JX244183.1/China/SDLY11/2009 (C4)	TCCAAGTCCAAGTACCCTTTAGT
190. JN256061.1/China/RDcells/G398-1037F/HeN/CHN/2009/C4a (C4)	TCCAAGTCCAAGTACCCTTTAGT
191. HQ825317.1/China/Shandong/EV71/JN200804/2008/C4a/2008 (C4)	TCCAAGTCCAAGTACCCTTTAGT
192. JF913464.1/China/Shandong/EV71/JN200803/2008/C4a/2008 (C4)	TCCAAGTCCAAGTACCCTTTAGT
193. JN256060.1/China/RDcellsG333-972F/HeN/CHN/2009/C4a (C4)	TCCAAGTCCAAGTATCCTTTAGT
194. JN256059.1/China/Henan/RDcells/G288-927F/HeN/CHN/2009/C4a/2009 (C4)	TCCAAGTCCAAGTACCCTTTAGT
195. JX678884.1/China/Shandong/SD09-21/SD/CHN/2009/C4a/2009 (C4)	TCCAAGTCCAAGTACCCTTTAGT
196. JX678883.1/China/Shandong/SD09-14/SD/CHN/2009/C4a/2009 (C4)	TCCAAGTCCAAGTACCCTTTAGT
197. JQ319054.1/China/Beijing/BJ09/07/C4a/2009 (C4)	TCCAAGTCCAAGTACCCTTTAGT
198. JF820315.1/China/AH01/C4/2010 (C4)	TCCAAGTCCAAGTACCCTTTAGT
199. JF820314.1/China/BJ02/C4/2010 (C4)	TCCAAGTCCAAGTACCCTTTAGT
200. JQ708209.1/China/isolateBJ/C4/2010 (C4)	TCCAAGTCCAAGTACCCTTTAGT
201. JF820316.1/China/BJ01/C4/2010 (C4)	TCCAAGTCCAAGTACCCTTTAGT
202. JF820313.1/China/LCH02/C4/2010 (C4)	TCCAAGTCCAAGTACCCTTTAGT
203. FJ606449.1/China/BJ08-Z020-1/2008 (C4)	TCCAAGTCCAAGTACCCTTTAGT
204. GU198371.1/China/FY08-C30-P14/2008 (C4)	TCCAAGTCCAAGTACCCTTTAGT
205. GU198370.1/China/C2/FY08-C30/2008 (C4)	TCCAAGTCCAAGTACCCTTTAGT
206. GU198367.1/China/Anhui/FY08-C30-P2/2008/C4a/2008 (C4)	TCCAAGTCCAAGTACCCTTTAGT
207. GU198369.1/China/C1/FY08-C30-P11/2008 (C4)	TCCAAGTCCAAGTACCCTTTAGT
208. GU198368.1/China/C1/FY08-C30-P9/2008 (C4)	TCCAAGTCCAAGTACCCTTTAGT
209. FJ439769.1/China/Anhui/Fuyang-0805/2008/C4a/2008 (C4)	TCCAAGTCCAAGTACCCTTTAGT
210. EU703814.1/China/Anhui/2008/EV71/Fuyang.Anhui.P.R.C/17.08/3/2008/C4a/2008 (C4)	TCCAAGTCTAAGTACCCTTTAGT
211. EU703812.1/China/Anhui/EV71/Fuyang.Anhui.P.R.C/17.08/1/2008/C4a/2008 (C4)	TCCAAGTCTAAGTACCCTTTAGT
212. JX678875.1/China/Anhui/FY17.08-4/AH/CHN/2008/C4a/2008 (C4)	TCCAAGTCTAAGTACCCTTTAGT
213. FJ158601.1/China/DTID/ZJU-74/2008 (C4)	TCCAAGTCCAAGTACCCTTTAGT
214. FJ158600.1/China/Zhejiang/DTID/ZJU-62/2008/C4a/2008 (C4)	TCCAAGTCCAAGTACCCTTTAGT

	3,190 3,200
Consensus	TCCAAGTCCAAGTACCCTTTAGT
	FL9R
[illegible]	[illegible]
214. FJ158600.1/China/Zhejiang/DTID/ZJU-62/2008/C4a/2008 (C4)	TCCAAGTCCAAGTACCCTTTAGT
215. FJ606448.1/China/BJ08-Z011-4/C4/2008 (C4)	TCCAAGTCCAAGTACCCTTTAGT
216. EU703813.1/China/Anhui/EV71/Fuyang.Anhui.P.R.C/17.08/2/2008/C4a/2008 (C4)	TCCAAGTCCAAGTACCCTTTAGT
217. JX678879.1/China/Anhui/FY17.08-8/AH/CHN/2008/C4a/2008 (C4)	TCCAAGTCCAAGTACCCTTTAGT
218. JX678877.1/China/Anhui/FY17.08-6/AH/CHN/2008/C4a/2008 (C4)	TCCAAGTCCAAGTACCCTTTAGT
219. JX678876.1/China/Anhui/FY17.08-5/AH/CHN/2008/C4a/2008 (C4)	TCCAAGTCCAAGTACCCTTTAGT
220. JF799986.1/China/LabstrainGuangdong/2009 (C4)	TCCAAGTCCAAGTACCCTTTAGT
221. JF738002.1/Thailand/THA-EV71-044/C4/2009 (C4)	TCCAAGTCCAAGTACCCTTTAGT
222. FJ607337.1/China/Shenzhen/121/SHENZHEN/08/China/HFMD-Fatal/2008/C4a/2008 (C4)	TCCAAGTCCAAGTACCCTTTAGT
223. FJ606450.1/China/BJ08-Z025-5/2008 (C4)	TCCAAGTCCAAGTACCCTTTAGT
224. HM622391.1/Taiwan/2008-00643/C2-like/2008 (C2)	TCAAAGTCCAAATACCCATTGGT
225. JQ280307.1/Taiwan/3149/C2/2008 (C2)	TCAAAGTCCAAATACCCATTGGT
226. HM622392.1/Taiwan/2008-07776/C2-like/2008 (C2)	TCAAAGTCCAAATACCCATTGGT
227. EU527983.1/Taiwan/2007-07364/2007/C5/2007 (C5)	TCGAAGTCCAAGTACCCTTTAGT
228. EF063152.1/Taiwan/E2005125-TW (C5)	TCGAAGTCCAAGTACCCTTTAGT
229. DQ341355.1/SouthKorea/06-KOR-00/C3/2000 (C3)	TCAAAATCCAAGTACCCATTGGT
230. DQ341356.1/SouthKorea/03-KOR-00/C3/2000 (C3)	TCAAAATCCAAGTACCCATTGGT
231. AF119795.2/Taiwan/TW/2272/98/C/1998 (UNKNOWN)	TCGAAGTCCAAGTACCCATTGGT
232. AF119796.3/Taiwan/TW/2086/98/C2/1998 (C2)	TCGAAGTCCAAGTACCCATTGGT
233. DQ060149.1/Taiwan/pinf7-54A/C2/2005 (C2)	TCGAAGTCCAAGTGCCCATTGGT
234. AF304457.1/Taiwan/5746/98/C2/1998 (C2)	TCGAAGTCCAAGTACCCATTGGT
235. AF176044.1/Taiwan/1245a/98/tw (C2)	TCGAAGTCCAAGTACCCATTGGT
236. JN544418.1/China/4643-TW98/C2/1998 (C2)	TCGAAGTCCAAGTACCCATTGGT
237. AF304458.1/Taiwan/Tainan/4643/98/C2 (C2)	TCGAAGTCCAAGTACCCATTGGT
238. AF304459.1/Taiwan/Tainan/6092/98/C2 (C2)	TCGAAGTCCAAGTACCCATTGGT
239. N544419.1/China/mousestrain/MP4/C2/2004 (C2)	TCGAAGTCCAAGTACCCATTGGT
240. AF136379.1/NCKU9822 (C2)	TCGAAGTCCAAGTACCCATTGGT
241. AB550333.1/Japan/Labstrain/1095-LPS1/C2/1997 (C2)	TCGAAATCCAAGTACCCATTAGT
242. AB550332.1/Japan/1095-org/C2/1997 (C2)	TCGAAATCCAAGTACCCATTAGT
243. JN992283.1/Australia/0964/SYD/98 (C2)	TCGAAGTCCAAGTACCCATTGGT
244. JX025559.1/Australia/Labstrain/LAZ60-TR.C2/2010 (C2)	TCGAAGTCCAAGTACCCATTGGT
245. DQ381846.1/Australia/6F/AUS/6/99/C2/1999 (C2)	TCGAAGTCCAAGTACCCATTGGT
246. DQ341357.1/Australia/7F-AUS-6-99 (C2)	TCGAAGTCCAAGTACCCATTGGT
247. AB575939.1/Netherlands/365/C2/2000 (C2)	TCGAAATCCAAGTACCCATTGGT
248. HQ647173.1/Canada/EV003-07/2006 (C2)	TCGAAGTCCAAGTACCCATTGGT
249. HQ647168.1/Canada/EV92-07/2007 (C2)	TCGAAGTCCAAGTACCCATTGGT
250. HQ647174.1/Cana/EV123-07/2007 (C2)	TCGAAGTCCAAGTACCCATTGGT
251. HQ647170.1/Canada/EV124-07/2007 (C2)	TCGAAGTCCAAGTACCCATTGGT
252. HQ647176.1/Canada/EV049-07/2007 (C2)	TCGAAGTCCAAGTACCCATTGGT
253. HQ647169.1/Canada/EV090-07/2007 (C2)	TCGAAGTCCAAGTACCCATTGGT
254. HQ647178.1/Canada/EV082-07/2007 (C2)	TCGAAGTCCAAGTACCCATTGGT
255. HQ647177.1/Canada/EV073-07/2007 (C2)	TCGAAGTCCAAGTACCCATTGGT
256. HQ647175.1/Canada/EV053-07/2007 (C2)	TCGAAGTCCAAGTACCCACTGGT
257. HQ647167.1/Canda/EV054-07/2007 (C2)	TCGAAGTCCAAGTACCCACTGGT
258. FJ172159.1/Singapore/NUH0075/SIN/08/2008 (C2)	TCGAAGTCCAAGTACCCACTGGT
259. AB575942.1/Netherlands/3692/C2/2007 (C2)	TCGAAGTCCAAGTACCCACTGGT
260. AB575941.1/Netherlands/2485/C2/2007 (C2)	TCGAAGTCCAAGTACCCACTGGT
261. JN835312.1/France//MRS/09/3663/C3/2009 (C3)	TCGAAGTCCAAGTACCCACTGGT
262. AB575948.1/Netherlands/10118/C2/2010 (C2)	TCGAAGTCCAAGTACCCACTGGT
263. AB575938.1/Netherlands/10098/C1/2010 (C1)	TCGAAATCCAAGTACCCTTTGGT
264. JF738000.1/Thailand/THA-EV71-002/C1/2009 (C1)	TCGAAATCCAAATACCCTCTGGT
265. EU414335.2/Switzerland/PLday1-CH-06/C1 (C1)	TCGAAATCTAAGTACCCTCTGGT
266. EU414334.2/Switzerland/PLday4-CH-06/C1 (C1)	TCGAAATCTAAGTACCCTCTGGT
267. EU414333.2/Switzerland/CSF-CH-06/C1 (C1)	TCGAAATCTAAGTACCCTCTGGT
268. EU414331.2/Switzerland/LR-CH-06/C1 (C1)	TCGAAATCTAAGTACCCTCTGGT
269. DQ341360.1/Malaysia/J115-MAL-01 (C1)	TCGAAGTCCAAGTACCCTTTGGT
270. DQ341358.1/Malaysia/S40221-SAR-00/C1/2000 (C1)	TCGAAATCCAAGTACCCTTTGGT
271. AB550341.1/Malaysia/Labstrain/KED005-LPS2/C1/1997 (C1)	TCGAAATCCAAGTACCCTTTGGT
272. AB550340.1/Malaysia/KED005-org/C1/1997 (C1)	TCGAAATCCAAGTACCCTTTGGT
273. DQ452074.1/Norway/804/NO/03/C1/2003 (C1)	TCGAAGTCCAAGTACCCTTTGGT
274. DQ341361.1/Australia/1M-AUS-12-00/C1 (C1)	TCGAAATCCAAGTACCCTTTGGT
275. AB575937.1/Netherlands/1416/C1/2001 (C1)	TCGAAATCCAAGTACCCTTTGGT
276. DQ341359.1/Malaysia/S10862-SAR-98/C1/1998 (C1)	TCGAAATCCAAGTACCCTTTGGT
277. HQ647172.1/Canada/EV063-04/1994 (C1)	TCGAAGTCCAAGTACCCTTTGGT
278. AB575936.1/Netherlands/9612/C1/1991 (C1)	TCGAAGTCCAAGTACCCTTTGGT
279. AB575935.1/Netherlands/480/C1/1991 (C1)	TCGAAGTCCAAGTACCCTTTGGT

	2,730 2,740
Consensus	G G A T T A G T T G G A G A G A T A G A T C T
	FL15-18F; Primer Bind
1. GU434678.1/China/Hubei/EV71-Hubei-09-China/B5/2009 (B5)	G G C T T A G T T G G G G A G A T A G A T C T
2. AB204853.1/BrCr-ts/A (A)	G G C T T A G T T G G G G A G A T A G A T C T
3. AB575911.1/Netherlands/10076/B0/1966 (B0)	G G T T T A G T C G G G G A G A T A G A C C T
4. AB575912.1/Netherlands/10857/B0/1966 (B0)	G G T T T A G T C G G G G A G A T A G A C C T
5. DQ341354.1/Singapore/3799-SIN-98/B3/1998 (B3)	G G T T T G G T A G G G G A G A T A G A T C T
6. DQ341368.1/Malaysia/MY104-9-SAR-97/B3 (B3)	G G T T T G G T A G G G G A G A T A G A T C T
7. DQ341367.1/Malaysia/MY821-3-SAR-97/B3/1997 (B3)	G G T T T G G T A G G G G A G A T A G A T C T
8. AB550335.1/Malaysia/Labstrain/SK-EV006-LPS1/B3/1997 (B3)	G G T T T G G T A G G G G A G A T A G A T C T
9. AB550334.1/Malaysia/SK-EV006-org/B3/1997 (B3)	G G T T T G G T A G G G G A G A T A G A T C T
10. AB469182.1/Malaysia/SK-EV006/Malaysia/97 (B3)	G G T T T G G T A G G G G A G A T A G A T C T
11. JQ950555.1/Australia26M/AUS/4/99/GuaR1/B3/2010 (B3)	G G T T T G G T A G G G G A G A T A A A T C T
12. EU364841.1/Australia/26M/AUS/4/99/B3/1999 (B3)	G G T T T G G T A G G G G A G A T A G A T C T
13. EF373575.1/Taiwan/E2002042-TW-CDC (B4)	G G A T T G G T A G G A G A A A T A G A T C T
14. FJ357377.1/Taiwan/S0296-TW00/B4/2000 (B4)	G G A T T G G T A G G A G A G A T A G A T C T
15. FJ357376.1/Taiwan/S0318-TW01/B4/2001 (B4)	G G A T T G G T A G G A G A G A T A G A T C T
16. DQ341366.1/Malaysia/SB2864-SAR-00/B4/2000 (B4)	G G C T T G G T A G G A G A G A T A G A T C T
17. DQ341365.1/Malaysia/PP37-MAL-01/B4/2001 (B4)	G G C T T G G T A G G A G A G A T A G A T C T
18. AF316321.2/Singapore/5865/sin/000009/B4 (B4)	G G C T T G G T A G G A G A G A T A G A T C T
19. AF352027.1/Singapore/5666/sin/002209/B4 (B4)	G G C T T G G T A G G A G A G A T A G A T C T
20. FJ357375.1/Taiwan/N7008-TW99/B4/1999 (B4)	G G T T T G G T A G G A G A G A T A G A T C T
21. AB550337.1/Japan/Labstrain/C7/Osaka-LPS1/B4/1997 (B4)	G G T T T G G T A G G A G A G A T A G A T C T
22. AB550336.1/Japan/C7/Osaka-org/B4/1997 (B4)	G G T T T G G T A G G A G A G A T A G A T C T
23. FJ461781.1/Singapore/NUH0083/SIN/08/B5/2008 (B5)	G G T T T G G T A G G A G A G A T A G A T C T
24. JF738001.1/Thailand/THA-EV71-019/B5/2009 (B5)	G G T T T G G T A G G A G A G A T A G A T C T
25. DQ341364.1/Singapore/5511-SIN-00 (B5)	G G T T T G G T A G G A G A G A T A G A T C T
26. JN992282.1/Brunei/BRU/2006/35334 (B5)	G G T T T A G T A G G A G A G A T A G A T C T
27. FJ357378.1/Taiwan/N2838-TW03/B5/2003 (B5)	G G T T T G G T A G G A G A G A T A G A T C T
28. DQ341363.1/Malaysia/S19841-SAR-03/B5/2003 (B5)	G G T T T G G T A G G A G A G A T A G A T C T
29. DQ341362.1/Malaysia/SB12736-SAR-03/B4/2003 (B4)	G G T T T G G T A G G A G A G A T A G A T C T
30. JN964686.1/China/Fujian/EV71/Xiamen/2009/B5/2009 (B5)	G G T T T G G T A G G A G A G A T A G A T C T
31. GQ231942.1/Taiwan/TW/96016/08/B5 (B5)	G G T T T G G T A G G A G A G A T A G A C C T
32. GQ231925.1/Taiwan/TW/1101/08/B5 (B5)	G G T T T G G T A G G A G A G A T A G A C C T
33. GQ231934.1/Taiwan/TW/70811/08/B5 (B5)	G G T T T G G T A G G A G A G A T A G A C C T
34. GQ231943.1/Taiwan/TW/96022/08/B5 (B5)	G G T T T G G T A G G A G A G A T A G A C C T
35. GQ231935.1/Taiwan//TW/70886/08/B5 (B5)	G G T T T G G T A G G A G A G A T A G A C C T
36. GQ231941.1/Taiwan/TW/96002/08/B5 (B5)	G G T T T G G T A G G A G A G A T A G A C C T
37. EU527985.1/Taiwan/2007-08747/2007/B5/2007 (B5)	G G T T T G G T A G G A G A G A T A G A C C T
38. FJ357385.1/Taiwan/M0380-TW08/B5/2008 (B5)	G G T T T G G T A G G A G A G A T A G A T C T
39. HM622390.1/Taiwan/2009-03531/B5/2009 (B5)	G G T T T G G T A G G A G A G A T A G A T C T
40. GQ231936.1/Taiwan/TW/70902/08/B5 (B5)	G G T T T G G T A G G A G A G A T A G A T C T
41. AB575927.1/Netherlands/11316/B2/1986 (B2)	G G C T T G G T A G G G G A G A T A G A T C T
42. AB575928.1/Netherlands/11590/B2/1986 (B2)	G G C T T A G T A G G G G A G A T A G A T C T
43. AB575923.1/Netherlands/20233/B2/1983 (B2)	G G C T T G G T A G G G G A G A T A G A T C T
44. U22522.1/USA/MS/7423/87/B2/1987 (B2)	G G C T T G G T A G G G G A G A T A G A T C T
45. FJ357384.1/Taiwan/266-TW86/B1/1986 (B1)	G G T T T G G T A G G A G A A A T A G A T C T
46. FJ357383.1/Taiwan/252-TW86/B1/1986 (B1)	G G C T T G G T A G G G G A A A T A G A T C T
47. FJ357380.1/Taiwan/237-TW86/B1/1986 (B1)	G G C T T G G T A G G G G A A A T A G A T C T
48. FJ357379.1/Taiwan/236-TW86/B1/1986 (B1)	G G C T T G G T A G G G G A A A T A G A T C T
49. FJ357382.1/Taiwan/238-TW86/B1/1986 (B1)	G G C T T G G T A G G G G A A A T A G A T C T
50. FJ357381.1/Taiwan/244-TW86/B1/1986 (B1)	G G C T T G G T A G G G G A A A T A G A T C T
51. AB575914.1/Netherlands/17000/B1/1971 (B1)	G G C T T A G T C G G G G A G A T A G A C C T
52. AB575913.1/Netherlands/11977/B1/1971 (B1)	G G C T T A G T C G G G G A G A T A G A T C T
53. AB575918.1/Netherlands/20574/B1/1978 (B1)	G G C T T G G T A G G G G A G A T A G A C C T
54. AB575917.1/Netherlands/10196/B1/1977 (B1)	G G C T T G G T A G G G G A G A T A G A T C T
55. HQ189392.1/Hungary/HUN/1978/1978 (B1)	G G C T T G G T G G G G G A G A T A G A C C T
56. AB575916.1/Netherlands/16173/B1/1976 (B1)	G G C T T G G T A G G G G A G A T A G A C C T
57. AB575915.1/Netherlands/9443/B1/1974 (B1)	G G C T T G G T A G G G G A G A T A G A C C T
58. AB482183.1/Japan/Nagoya/B1/1973 (B1)	G G C T T G G T A G G G G A G A T A G A C C T
59. HQ423143.1/China/Yunnan/KM186/09/2009/C4a/2009 (C4)	G G A T T G G T T G G A G A G A T A G A T C T
60. HQ423142.1/China/KMM/09/2009 (C4)	G G A T T A G T T G G G G A G A T A G A T C T
61. JQ742001.1/China/Guangxi/AFP2001064/EV71/GX/CHN/2001/C4b/2001 (C4)	G G A T T A G T T G G A G A A A T A G A C C T
62. EU131776.1/Taiwan/N3340-TW-02/C4b/2002 (C4)	G G A T T A G T T G G T G A G A T A G A C C T
63. JX678885.1/China/Shanghai/SH-17/SH/CHN/2002/C4b/2002 (C4)	G G G T T A G T T G G A G A G A T A G A C C T
64. JQ742002.1/China/AFP2001071/EV71/GX/CHN/2001/C4b/2001 (C4)	G G A T T A G T T G G A G A G A T A G A C C T
65. GU350629.1/China/AnHui/Fuyang-0805a (C4)	G G A T T A G T T G G A G A G A T A G A C C T
66. FJ194964.1/China/Guangdong/EV71/GDFS/3/2008/C4b/2008 (C4)	G G A T T A G T T G G A G A G A T A G A T C T
67. GQ994989.1/China/Chongqing/Chongqing1-09-China/2009/C4b/2009 (C4)	G G A T T A G T T G G G G A G A T A G A T C T
68. HM807310.1/Taiwan/cmuh-050530-5/2005 (C4)	G G A T T A G T T G G A G A G A T A G A T C T
69. AY465356.1/China/Shenzhen/SHZH03/C4b/2003 (C4)	G G A T T A G T T G G A G A G A T A G A C C T
70. JX678874.1/China/Chongqing/CQ03-1/CQ/CHN/2003/C4b/2003 (C4)	G G A T T A G T T G G A G A G A T A G A T C T
71. HQ647171.1/Canada/EV141-06/2006/C4a/2006 (C4)	G G A T T A G T T G G A G A G A T A G A T C T
72. HQ647180.1/Canada/EV034-06/2006 (C4)	G G A T T A G T T G G A G A G A T A G A T C T

	2,730 … 2,740
Consensus	GGATTAGTTGGAGAGATAGATCT
	FL15-18F; Primer Bind
72. HQ647180.1/Canada/EV034-06/2006 (C4)	GGATTAGTTGGAGAGATAGATCT
73. HQ647179.1/Canada/EV144-06/2006 (C4)	GGATTAGTTGGAGAGATAGATCT
74. JX678886.1/China/Shanghai/SH-6/SH/CHN/2002/C4b/2002 (C4)	GGATTAGTTGGAGAGATAGATCT
75. AB550339.1/Japan/Labstrain/75-Yamagata-LPS1/C4/2003 (C4)	GGATTGGTTGGAGAGATAGATCT
76. AB550338.1/Japan/75-Yamagata-org/C4/2003 (C4)	GGATTGGTTGGAGAGATAGATCT
77. DQ133459.1/Taiwan/1235/C4/2004 (C4)	GGATTAGTTGGAGAGATAGATCT
78. DQ133458.1/Taiwan/984/C4/2004 (C4)	GGATTAGTTGGAGAGATAGATCT
79. FJ357373.1/Taiwan/S0584-TW04/C4/2004 (C4)	GGATTAGTTGGAGAGATAGATCT
80. EF373576.1/Taiwan/E2004104-TW-CDC (C4)	GGATTAGTTGGAGAGATAGATCT
81. GQ231938.1/TaiwanTW/71552/05 (C4)	GGGTTAGTTGGAGAGATAGATCT
82. GQ231926.1/Taiwan/TW/1956/05/C4a/2005 (C4)	GGGTTAGTTGGAGAGATAGATCT
83. GQ231940.1/Taiwan/TW/72232/04/C4 (C4)	GGATTAGTTGGAGAGATAGATCT
84. GQ231937.1/Taiwan/TW/71428/05/C4 (C4)	GGGTTAGTTGGAGAGATAGATCT
85. GQ231939.1/Taiwan/TW/71595/04/C4 (C4)	GGATTAGTTGGAGAGATAGATCT
86. GQ231929.1/Taiwan/TW/2728/04/C4a/2004 (C4)	GGATTAGTTGGAGAGATAGATCT
87. FJ357374.1/Taiwan/N2121-TW05/C4/2005 (C4)	GGATTAGTTGGAGAGATAGATCT
88. GQ231931.1/Taiwan/TW/2824/04/C4 (C4)	GGGTTAGTTGGAGAGATAGATCT
89. GQ231928.1/Taiwan/TW/2639/04/C4 (C4)	GGATTAGTTGGAGAGATAGATCT
90. GQ231927.1/Taiwan/TW/2429/04/C4 (C4)	GGATTAGTTGGAGAGATAGATCT
91. GQ231930.1/Taiwan/TW/2815/04/C4 (C4)	GGATTAGTTGGAGAGATAGATCT
92. JQ965759.1/Vietnam/540V/VNM/05/C4/2005 (C4)	GGATTAGTTGGAGAGATAGATCT
93. GQ231932.1/Taiwan/TW/2871/04/C4 (C4)	GGATTAGTTGGAGAGATAGATCT
94. GQ994992.1/China/Henan/Henan2-09-China/2009/C4a/2009 (C4)	GGATTAGTTGGAGAGATAGATCT
95. GQ279369.1/China/SZ/HK08-5/2008 (C4)	GGATTAGTTGGAGAGATAGATCT
96. JX244184.1/China/SDLY48/C/2009 (C4)	GGGTTAGTTGGAGAGATAGATCT
97. FJ606447.1/China/Beijing/BJ08-Z004-3/2008/C4a/2008 (C4)	GGATTGGTTGGAGAAATAGATCT
98. JQ681218.1/China/Henan/H8-1/C4a/2008 (C4)	GGATTAGTTGGAGAGATAGATCT
99. GU196833.1/China/Henan1-09-China/2009 (C4)	GGATTAGTTGGAGAGATAGATCT
100. HQ129932.1/China/BJ06-SJS06/06/C4/2006 (C4)	GGATTAGTTGGAGAGATAGATCT
101. FJ607336.1/China/28/SHENZHEN/08/China/HFMD/2008/2008 (C4)	GGATTAGTTGGGGAGATAGATCT
102. FJ828519.1/China/BJ08/C4/2008 (C4)	GGATTAGTTGGAGAGATAGATCT
103. FJ360545.1/China/GZ-08-02/C4/2008 (C4)	GGATTAGTTGGAGAGATAGATCT
104. JX244182.1/China/SDLY1/2009 (C4)	GGATTAGTCGGAGAGATAGATCT
105. JF820312.1/China/LCH01/C4/2010 (C4)	GGATTAGTTGGAGAGATAGATCT
106. HM002488.1/China/BJ366/2009 (C4)	GGATTAGTTGGAGAGATAGATCT
107. GQ231933.1/Taiwan/TW/70516/08/C4a/2008 (C4)	GGATTAGTTGGAGAGATAGATCT
108. KC109780.1/China/202/Jingdezhen/China/HFMDSevere/2011/C4/2011 (C4)	GGGTTAGTTGGAGAGATAGATCT
109. HM002485.1/China/BJ972008 (C4)	GGATTAGTTGGAGAGATAGATCT
110. GU366191.1/China/Henan/Henan10-08-China/2008/C4a/2008 (C4)	GGATTAGTTGGAGAGATAGATCT
111. FJ713137.1/Shanghai/Shanghai-036-2009/C4a/2009 (C4)	GGATTAGTTGGAGAGATAGATCT
112. EU753384.2/China/Shandong/522-04T/SD/CHN/07/C4a/2007 (C4)	GGATTAGTTGGAGAGATAGATCT
113. HM003207.1/China/87-2008-Xi'an-Shaanxi/2008 (C4)	GGATTAGTTGGAGAGATAGATCT
114. EU753365.2/China/Shandong/518-03F/SD/CHN/07/C4a/2007 (C4)	GGATTAGTTGGAGAGATAGATCT
115. EU753407.2/China/Shandong/TC03F/SD/CHN/07/C4a/2007 (C4)	GGATTAGTTGGAGAGATAGATCT
116. GQ994988.1/China/Anhui/Anhui1-09-China/C4a/2009 (C4)	GGATTAGTTGGAGAGATAGATCT
117. JQ708210.1/China/isolateTS/C4/2009 (C4)	GGATTAGTTGGAGAGATAGATCT
118. HQ611148.1/China/AH08/06/C4/2008 (C4)	GGATTAGTTGGAGAGATAGATCT
119. EU753398.2/China/Shandong/523-07T/SD/CHN/07/2007 (C4)	GGATTAGTTGGAGAGGTAGATCT
120. EU753397.2/China/Shandong/523-05T/SD/CHN/07/C4a/2007 (C4)	GGATTAGTTGGAGAGATAGATCT
121. JX678882.1/China/Shandong/SD07-1/SD/CHN/2007/C4a/2007 (C4)	GGATTAGTTGGAGAGATAGACCT
122. EU753375.2/China/Shandong/521-18S/SD/CHN/07/C4a/2007 (C4)	GGATTAGTTGGAGAGATAGATCT
123. HM002487.1/China/BJ303/2008 (C4)	GGATTAGTTGGAGAGATAGATCT
124. JX678878.1/China/Anhui/FY17.08-7/AH/CHN/2008/C4a/2008 (C4)	GGATTAGTTGGAGAGATAGATCT
125. JX025561.1/China/FY7VP5/AH/CHN/2008 (C4)	GGATTAGTTGGAGAGATAGATCT
126. JQ316638.1/China/HQ09231463/2011 (C4)	GGATTAGTTGGAGAGATAGATCT
127. HQ426649.1/China/Anhui/2010FJLY008/2010/C4a/2010 (C4)	GGATTAGTTGGAGAGATAGATCT
128. GQ994991.1/China/Chongqing3-09-China/2009 (C4)	GGATTAGTTGGAGAGATAGATCT
129. HM053670.1/China/strain-BJ393/2009 (C4)	GGATTAGTTGGAGAGATAGATCT
130. FJ360544.1/China/GZ-08-01/C4/2008 (C4)	GGATTAGTTGGAGAGATAGATCT
131. HQ998852.1/China/Henan/EV71/Henan/106/2009/C4a/2009 (C4)	GGATTAGTTGGAGAGATAGATCT
132. JX244187.1/China/SDLY153/2010 (C4)	GGATTAGTTGGAGAGATAGATCT
133. GQ994990.1China/Chongqing2-09-China/C4/2009 (C4)	GGATTGGTTGGAGAGATAGATCT
134. EU864507.1/China/Zhejiang/EV71/Zhejiang08/2008/C4a/2008 (C4)	GGATTAGTTGGAGAGATAGATCT
135. FJ194965.1/China/EV71/GDSG/17/2008/C/2008 (C4)	GGATTAGTTGGAGAGATAGATCT
136. FJ607334.1/China/SHENZHEN/08/China/HFMD/2008/2008 (C4)	GGATTAGTTGGAGAGATAGACCT
137. GQ279370.1/China/SZ/HK08-6/2008 (C4)	GGATTAGTTGGAGAGATAGACCT
138. FJ607335.1/China/4/SHENZHEN/08/China/HFMD/2008/2008 (C4)	GGATTAGTTGGAGAGATAGACCT
139. JQ736684.2/China/SHAPHC695F/SH/CHN/10/C4a/2010 (C4)	GGATTAGTTGGAGAGATAGATCT
140. JQ074187.1/China/Jinan1002/2010 (C4)	GGATTAGTTGGAGAGATAGATCT
141. JQ074189.1/China/Jinan1005/2010 (C4)	GGATTAGTTGGAGAGATAGATCT
142. JQ074188.1/China/EV71/Jinan1004/2010 (C4)	GGATTAGTTGGAGAGATAGATCT
143. JF830007.1/China/Zhejiang/EV71/Ningbo.CHN/065/2010/C4a/2010 (C4)	GGATTGGTTGGAGAGATAGATCT

	2,730 … 2,740
Consensus	GGATTAGTTGGAGAGATAGATCT
	FL15-18F; Primer Bind
[illegible]	[illegible]
143. JF830007.1/China/Zhejiang/EV71/Ningbo.CHN/065/2010/C4a/2010 (C4)	GGATTGGTTGGAGAGATAGATCT
144. JX111893.1/China/GD-DG2011-9/C4/2011 (C4)	GGATTAGTTGGGGAGATAGACCT
145. JX111891.1/China/GD-DG2011-5/C4/2011 (C4)	GGATTAGTTGGGGAGATAGACCT
146. JX111889.1/China/GD-DG2011-2/C4/2011 (C4)	GGATTAGTTGGGGAGATAGACCT
147. JX111888.1/China/GD-DG2011-1/C4/2011 (C4)	GGATTAGTTGGGGAGATAGACCT
148. JX111892.1/China/GD-DG2011-8/C4/2011 (C4)	GGATTAGTTGGGGAGATAGACCT
149. JX111890.1/China/GD-DG2011-3/C4/2011 (C4)	GGATTAGTTGGGGAGATAGACCT
150. JQ806378.1/China/Jiangxi/35/Jingdezhen/China/HFMDSevere/2011/C4a/2011 (C4)	GGATTAGTTGGAGAGATAGATCT
151. JX017384.1/China/01011Y/2011 (C4)	GGATTAGTTGGAGAGATAGATCT
152. HQ891925.1/China/Shanghai-36-2009/C4/2009 (C4)	GGATTAGTTGGAGAGATAGATCT
153. JX244186.1/China/SDLY107/2010 (C4)	GGATTAGTTGGAGAGATAGATCT
154. HQ850973.1/China/003/XM/2010/2010 (C4)	GGATTAGTTGGAGAGATAGATCT
155. FJ360546.1/China/GZ-08-03/C4/2008 (C4)	GGATTAGTTGGAGAGATAGATCT
156. HQ891926.1/China/Shanghai-51-2009/C4/2009 (C4)	GGATTAGTTGGAGAGATAGATCT
157. GQ892830.1/China/GX/LZ (C4)	GGATTAGTTGGAGAGATAGATCT
158. JN020147.1/China/Henan/Luoyang/2011/C4a/2011 (C4)	GGATTAGTTGGAGAGATAGATCT
159. JQ517316.1/China/Henan/Kaifeng/2010/C4a/2010 (C4)	GGATTAGTTGGAGAGATAGATCT
160. JN052925.1/China/Henan/Nanyang/2011-China/2011/C4a/2011 (C4)	GGATTAGTTGGAGAGATAGATCT
161. HQ828086.1/China/Zhejiang/NBChina01/2010/C4a/2010 (C4)	GGATTAGTTGGAGAGATAGATCT
162. JX986739.1/China/Wuhan1143/HuB/CHN/2011/C4/2011 (C4)	GGATTAGTTGGAGAGATAGATCT
163. HQ407557.1/China/Liangning/LN009/2010/C4a/2010 (C4)	GGATTAGTTGGAGAGATAGATCT
164. JQ074190.1/China/Shandong/Jinan1006/2010/C4a/2010 (C4)	GGATTAGTTGGAGAGATAGATCT
165. JN001860.1/China/Zhejiang/NB/2010/01/C4a/2010 (C4)	GGATTGGTTGGAGAGATAGATCT
166. JQ804832.1/China/EV71/Hubei-XF/CHN/2010/C4/2010 (C4)	GGATTAGTTGGAGAGATAGATCT
167. JQ514785.1/China/BC08/C4a/2008 (C4)	GGATTAGTTGGAGAGATAGATCT
168. HM053669.1/China/BJ293/2008 (C4)	GGATTAGTTGGAGAAATAGATCT
169. HM002484.1/China/BJ67/2008 (C4)	GGATTAGTTGGAGAGATAGATCT
170. GU396280.1/China/Gansu/EV71/Lanzhou01/GS/CHN/09/C4a/2009 (C4)	GGATTAGTTGGAGAGATAGATCT
171. HQ400942.1/China/HZ08/Hangzhou/2008/C4/2008 (C4)	GGATTAGTTGGAGAGATAGATCT
172. HQ891929.1/China/Shanghai-118-2009/C4/2009 (C4)	GGATTAGTTGGAGAGATAGATCT
173. FJ607338.1/China/Shenzhen/605/SHENZHEN/08/China/HFMD/C4a/2008 (C4)	GGATTAGTTGGAGAGATAGATCT
174. JX986737.1/China/Wuhan1042/HuB/CHN/2011/C4/2011 (C4)	GGATTAGTTGGAGAGATAGATCT
175. HQ891927.1/China/Shanghai-64-2009/C4/2009 (C4)	GGATTAGTTGGAGAGATAGATCT
176. HQ891928.1/China/Shanghai-117-2009/C4/2009 (C4)	GGATTAGTTGGAGAGATAGATCT
177. HQ891924.1/China/Shanghai-28-2009/C4/2009 (C4)	GGATTAGTTGGAGAGACAGATCT
178. HQ891923.1/China/Shanghai/Shanghai-27-2009/C4a/2009 (C4)	GGATTAGTTGGAGAGATAGATCT
179. HM002489.1/China/Beijing/Beijing398/2009/C4a/2009 (C4)	GGACTAGTTGGAGAGATAGATCT
180. JN256063.1/China/RDcells/M186-1179F/HeN/CHN/2009/C4a (C4)	GGATTAGTTGGAGAGATAGATCT
181. JN256062.1/China/RDcells/M183-1176F/HeN/CHN/2009/C4a (C4)	GGATTAGTTGGAGAGATAGATCT
182. JN256064.1/China/Henan/RDcells/M188-1181F/HeN/CHN/2009/C4a/2009 (C4)	GGATTAGTTGGAGAGATAGATCT
183. JX678881.1/China/Henan/HeN09-17/HeN/CHN/2009/C4a/2009 (C4)	GGATTAGTTGGAGAGATAGATCT
184. JX678880.1/China/Henan/HeN09-1/HeN/CHN/2009/C4a/2009 (C4)	GGATTAGTTGGAGAGATAGATCT
185. EU812515.1/China/Anhui/FY23/2008/C4a/2008 (C4)	GGATTAGTTGGAGAGATAGATCT
186. GU459071.1/China/FY23-K14/C4/2008 (C4)	GGATTAGTTGGAGAGATAGATCT
187. GU459070.1/China/FY23-K12/C4/2008 (C4)	GGATTAGTTGGAGAGATAGATCT
188. JX244185.1/China/SDLY96/2009 (C4)	GGATTAGTTGGAGAGATAGATCT
189. JX244183.1/China/SDLY11/2009 (C4)	GGATTAGTTGGAGAGATAGATCT
190. JN256061.1/China/RDcells/G398-1037F/HeN/CHN/2009/C4a (C4)	GGACTAGTTGGAGAGATAGATCT
191. HQ825317.1/China/Shandong/EV71/JN200804/2008/C4a/2008 (C4)	GGATTAGTTGGAGAGATAGATCT
192. JF913464.1/China/Shandong/EV71/JN200803/2008/C4a/2008 (C4)	GGATTAGTTGGAGAGATAGATCT
193. JN256060.1/China/RDcellsG333-972F/HeN/CHN/2009/C4a (C4)	GGATTAGTTGGAGAGATAGATCT
194. JN256059.1/China/Henan/RDcells/G288-927F/HeN/CHN/2009/C4a/2009 (C4)	GGATTAGTTGGAGAGATAGATCT
195. JX678884.1/China/Shandong/SD09-21/SD/CHN/2009/C4a/2009 (C4)	GGATTAGTCGGAGAGATAGATCT
196. JX678883.1/China/Shandong/SD09-14/SD/CHN/2009/C4a/2009 (C4)	GGATTAGTTGGAGAGATAGATCT
197. JQ319054.1/China/Beijing/BJ09/07/C4a/2009 (C4)	GGATTAGTTGGAGAGATAGATCT
198. JF820315.1/China/AH01/C4/2010 (C4)	GGATTAGTTGGAGAGATAGATCT
199. JF820314.1/China/BJ02/C4/2010 (C4)	GGATTAGTTGGAGAGATAGATCT
200. JQ708209.1/China/isolateBJ/C4/2010 (C4)	GGATTAGTTGGAGAGATAGATCT
201. JF820316.1/China/BJ01/C4/2010 (C4)	GGATTAGTTGGAGAGATAGATCT
202. JF820313.1/China/LCH02/C4/2010 (C4)	GGATTAGTTGGAGAGATAGATCT
203. FJ606449.1/China/BJ08-Z020-1/2008 (C4)	GGATTAGTTGGAGAGATAGATCT
204. GU198371.1/China/FY08-C30-P14/2008 (C4)	GGATTAGTTGGAGAGATAGATCT
205. GU198370.1/China/C2/FY08-C30/2008 (C4)	GGATTAGTTGGAGAGATAGATCT
206. GU198367.1/China/Anhui/FY08-C30-P2/2008/C4a/2008 (C4)	GGATTAGTTGGAGAGATAGATCT
207. GU198369.1/China/C1/FY08-C30-P11/2008 (C4)	GGATTAGTTGGAGAGATAGATCT
208. GU198368.1/China/C1/FY08-C30-P9/2008 (C4)	GGATTAGTTGGAGAGATAGATCT
209. FJ439769.1/China/Anhui/Fuyang-0805/2008/C4a/2008 (C4)	GGATTAGTTGGAGAAATAGATCT
210. EU703814.1/China/Anhui/2008/EV71/Fuyang.Anhui.P.R.C/17.08/3/2008/C4a/2008 (C4)	GGATTAGTTGGAGAGATAGATCT
211. EU703812.1/China/Anhui/EV71/Fuyang.Anhui.P.R.C/17.08/1/2008/C4a/2008 (C4)	GGATTAGTTGGAGAGATAGATCT
212. JX678875.1/China/Anhui/FY17.08-4/AH/CHN/2008/C4a/2008 (C4)	GGATTAGTTGGAGAGATAGATCT
213. FJ158601.1/China/DTID/ZJU-74/2008 (C4)	GGATTAGTTGGAGAGATAGATCT
214. FJ158600.1/China/Zhejiang/DTID/ZJU-62/2008/C4a/2008 (C4)	GGATTAGTTGGAGAGATAGATCT

	2,730 2,740
Consensus	GGATTAGTTGGAGAGATAGATCT
	FL15-18F; Primer Bind
213. FJ158601.1/China/DTID/ZJU-74/2008 (C4)	GGATTAGTTGGAGAGATAGATCT
214. FJ158600.1/China/Zhejiang/DTID/ZJU-62/2008/C4a/2008 (C4)	GGATTAGTTGGAGAGATAGATCT
215. FJ606448.1/China/BJ08-Z011-4/C4/2008 (C4)	GGATTAGTTGGAGAGATAGATCT
216. EU703813.1/China/Anhui/EV71/Fuyang.Anhui.P.R.C/17.08/2/2008/C4a/2008 (C4)	GGATTAGTTGGAGAGATAGATCT
217. JX678879.1/China/Anhui/FY17.08-8/AH/CHN/2008/C4a/2008 (C4)	GGATTAGTTGGAGAGATAGATCT
218. JX678877.1/China/Anhui/FY17.08-6/AH/CHN/2008/C4a/2008 (C4)	GGATTAGTTGGAGAGATAGATCT
219. JX678876.1/China/Anhui/FY17.08-5/AH/CHN/2008/C4a/2008 (C4)	GGATTAGTTGGAGAGATAGATCT
220. JF799986.1/China/LabstrainGuangdong/2009 (C4)	GGATTAGTTGGAGAGATAGATCT
221. JF738002.1/Thailand/THA-EV71-044/C4/2009 (C4)	GGATTAGTTGGAGAGATAGATCT
222. FJ607337.1/China/Shenzhen/121/SHENZHEN/08/China/HFMD-Fatal/2008/C4a/2008 (C4)	GGATTAGTTGGAGAGATAGATCT
223. FJ606450.1/China/BJ08-Z025-5/2008 (C4)	GGATTAGTTGGAGAGATAGATCT
224. HM622391.1/Taiwan/2008-00643/C2-like/2008 (C2)	GGACTGGTTGGAGAGATAGATCT
225. JQ280307.1/Taiwan/3149/C2/2008 (C2)	GGATTGGTTGGAGAGATAGATCT
226. HM622392.1/Taiwan/2008-07776/C2-like/2008 (C2)	GGATTGGTTGGAGAGATAGATCT
227. EU527983.1/Taiwan/2007-07364/2007/C5/2007 (C5)	GGGTTAGTTGGAGAGATAGACCT
228. EF063152.1/Taiwan/E2005125-TW (C5)	GGGTTAGTTGGAGAGATAGACCT
229. DQ341355.1/SouthKorea/06-KOR-00/C3/2000 (C3)	GGGCTAGTTGGAGAGATAGATCT
230. DQ341356.1/SouthKorea/03-KOR-00/C3/2000 (C3)	GGGTTAGTTGGAGAGATAGACCT
231. AF119795.2/Taiwan/TW/2272/98/C/1998 (UNKNOWN)	GGCTTGGTAGGGGAAATAGATCT
232. AF119796.3/Taiwan/TW/2086/98/C2/1998 (C2)	GGATTAGTTGGAGAGATAGACCT
233. DQ060149.1/Taiwan/pinf7-54A/C2/2005 (C2)	GGATTAGTTGGAGAGATAGACCT
234. AF304457.1/Taiwan/5746/98/C2/1998 (C2)	GGATTAGTTGGAGAGATAGACCT
235. AF176044.1/Taiwan/1245a/98/tw (C2)	GGATTAGTTGGAGAGATAGACCT
236. JN544418.1/China/4643-TW98/C2/1998 (C2)	GGATTAGTTGGAGAGATAGACCT
237. AF304458.1/Taiwan/Tainan/4643/98/C2 (C2)	GGATTAGTTGGAGAGATAGACCT
238. AF304459.1/Taiwan/Tainan/6092/98/C2 (C2)	GGATTAGTTGGAGAGATAGACCT
239. N544419.1/China/mousestrain/MP4/C2/2004 (C2)	GGATTAGTTGGAGAGATAGACCT
240. AF136379.1/NCKU9822 (C2)	GGATTAGTTGGAGAGATAGACCT
241. AB550333.1/Japan/Labstrain/1095-LPS1/C2/1997 (C2)	GGATTAGTTGGAGAGATAGACCT
242. AB550332.1/Japan/1095-org/C2/1997 (C2)	GGATTAGTTGGAGAGATAGACCT
243. JN992283.1/Australia/0964/SYD/98 (C2)	GGATTAGTTGGAGAGATAGACCT
244. JX025559.1/Australia/Labstrain/LAZ60-TR.C2/2010 (C2)	GGATTAGTTGGAGAGATAGACCT
245. DQ381846.1/Australia/6F/AUS/6/99/C2/1999 (C2)	GGATTAGTTGGAGAGATAGACCT
246. DQ341357.1/Australia/7F-AUS-6-99 (C2)	GGATTAGTTGGAGAGATAGACCT
247. AB575939.1/Netherlands/365/C2/2000 (C2)	GGATTAGTTGGAGAGATAGACCT
248. HQ647173.1/Canada/EV003-07/2006 (C2)	GGGTTAGTTGGAGAGATAGACCT
249. HQ647168.1/Canada/EV92-07/2007 (C2)	GGGTTAGTTGGAGAGATAGACCT
250. HQ647174.1/Cana/EV123-07/2007 (C2)	GGGTTAGTTGGAGAGATAGACCT
251. HQ647170.1/Canada/EV124-07/2007 (C2)	GGGTTAGTTGGAGAGATAGACCT
252. HQ647176.1/Canada/EV049-07/2007 (C2)	GGGTTAGTTGGAGAGATAGACCT
253. HQ647169.1/Canada/EV090-07/2007 (C2)	GGGTTAGTTGGAGAGATAGACCT
254. HQ647178.1/Canada/EV082-07/2007 (C2)	GGGTTAGTTGGAGAGATAGACCT
255. HQ647177.1/Canada/EV073-07/2007 (C2)	GGGTTAGTTGGAGAGATAGACCT
256. HQ647175.1/Canada/EV053-07/2007 (C2)	GGATTAGTTGGAGAAATAGACCT
257. HQ647167.1/Canda/EV054-07/2007 (C2)	GGATTAGTTGGAGAAATAGACCT
258. FJ172159.1/Singapore/NUH0075/SIN/08/2008 (C2)	GGATTAGTTGGAGAAATAGACCT
259. AB575942.1/Netherlands/3692/C2/2007 (C2)	GGATTAGTTGGAGAAATAGACCT
260. AB575941.1/Netherlands/2485/C2/2007 (C2)	GGATTAGTTGGAGAAATAGACCT
261. JN835312.1/France//MRS/09/3663/C3/2009 (C3)	GGATTGGTTGGAGAAATAGACCT
262. AB575948.1/Netherlands/10118/C2/2010 (C2)	GGATTAGTTGGAGAAATAGACCT
263. AB575938.1/Netherlands/10098/C1/2010 (C1)	GGGTTAGTTGGGGAGATAGACCT
264. JF738000.1/Thailand/THA-EV71-002/C1/2009 (C1)	GGGCTAGTTGGGGAGATAGACCT
265. EU414335.2/Switzerland/PLday1-CH-06/C1 (C1)	GGGCTAGTTGGGGAGATAGACCT
266. EU414334.2/Switzerland/PLday4-CH-06/C1 (C1)	GGGCTAGTTGGGGAGATAGACCT
267. EU414333.2/Switzerland/CSF-CH-06/C1 (C1)	GGGCTAGTTGGGGAGATAGACCT
268. EU414331.2/Switzerland/LR-CH-06/C1 (C1)	GGGCTAGTTGGGGAGATAGACCT
269. DQ341360.1/Malaysia/J115-MAL-01 (C1)	GGGCTAGTTGGAGAGATAGACCT
270. DQ341358.1/Malaysia/S40221-SAR-00/C1/2000 (C1)	GGGCTAGTTGGAGAGATAGACCT
271. AB550341.1/Malaysia/Labstrain/KED005-LPS2/C1/1997 (C1)	GGGCTAGTTGGAGAGATAGACCT
272. AB550340.1/Malaysia/KED005-org/C1/1997 (C1)	GGGCTAGTTGGAGAGATAGACCT
273. DQ452074.1/Norway/804/NO/03/C1/2003 (C1)	GGGCTAGTTGGGGAGATAGACCT
274. DQ341361.1/Australia/1M-AUS-12-00/C1 (C1)	GGGCTAGTTGGAGAGATAGACCT
275. AB575937.1/Netherlands/1416/C1/2001 (C1)	GGGCTAGTTGGAGAGATAGACCT
276. DQ341359.1/Malaysia/S10862-SAR-98/C1/1998 (C1)	GGACTAGTTGGGGAGATAGACCT
277. HQ647172.1/Canada/EV063-04/1994 (C1)	GGGTTAGTTGGGGAGATAGACCT
278. AB575936.1/Netherlands/9612/C1/1991 (C1)	GGGTTAGTTGGGGAAATAGATCT
279. AB575935.1/Netherlands/480/C1/1991 (C1)	GGGTTAGTTGGGGAGATAGACCT

	4,840 · 4,850 (FL19-20R)
Consensus	ATGGACTGTGACATTGAAGTGAC
1. GU434678.1/China/Hubei/EV71-Hubei-09-China/B5/2009 (B5)	ATGGACTGCGATATAGAAGTGAC
2. AB204853.1/BrCr-ts/A (A)	ATGGACTGCGATATAGAAGTGAC
3. AB575911.1/Netherlands/10076/B0/1966 (B0)	ATGGATTGTGACATCGAAGTGAC
4. AB575912.1/Netherlands/10857/B0/1966 (B0)	ATGGATTGTGACATCGAAGTGAC
5. DQ341354.1/Singapore/3799-SIN-98/B3/1998 (B3)	ATGGATTGTGATATCGAAGTCAC
6. DQ341368.1/Malaysia/MY104-9-SAR-97/B3 (B3)	ATGGATTGTGATATCGAAGTCAC
7. DQ341367.1/Malaysia/MY821-3-SAR-97/B3/1997 (B3)	ATGGATTGTGATATCGAAGTCAC
8. AB550335.1/Malaysia/Labstrain/SK-EV006-LPS1/B3/1997 (B3)	ATGGATTGTGATATCGAAGTCAC
9. AB550334.1/Malaysia/SK-EV006-org/B3/1997 (B3)	ATGGATTGTGATATCGAAGTCAC
10. AB469182.1/Malaysia/SK-EV006/Malaysia/97 (B3)	ATGGATTGTGATATCGAAGTCAC
11. JQ950555.1/Australia26M/AUS/4/99/GuaR1/B3/2010 (B3)	ATGGATTGTGATATCGAAGTCAC
12. EU364841.1/Australia/26M/AUS/4/99/B3/1999 (B3)	ATGGATTGTGATATCGAAGTCAC
13. EF373575.1/Taiwan/E2002042-TW-CDC (B4)	ATGGATTGTGACATTGAGGTGAC
14. FJ357377.1/Taiwan/S0296-TW00/B4/2000 (B4)	ATGGATTGTGACATTGAGGTGAC
15. FJ357376.1/Taiwan/S0318-TW01/B4/2001 (B4)	ATGGATTGTGACATTGAGGTGAC
16. DQ341366.1/Malaysia/SB2864-SAR-00/B4/2000 (B4)	ATGGATTGCGACATTGAGGTCAC
17. DQ341365.1/Malaysia/PP37-MAL-01/B4/2001 (B4)	ATGGATTGCGACATTGAGGTCAC
18. AF316321.2/Singapore/5865/sin/000009/B4 (B4)	ATGGATTGCGACATTGAGGTCAC
19. AF352027.1/Singapore/5666/sin/002209/B4 (B4)	ATGGATTGCGACATTGAGGTCAC
20. FJ357375.1/Taiwan/N7008-TW99/B4/1999 (B4)	ATGGATTGCGACATTGAGGTGAC
21. AB550337.1/Japan/Labstrain/C7/Osaka-LPS1/B4/1997 (B4)	ATGGATTGCGACATTGAGGTGAC
22. AB550336.1/Japan/C7/Osaka-org/B4/1997 (B4)	ATGGATTGCGACATTGAGGTGAC
23. FJ461781.1/Singapore/NUH0083/SIN/08/B5/2008 (B5)	ATGGATTGTGACATTGAGGTGAC
24. JF738001.1/Thailand/THA-EV71-019/B5/2009 (B5)	ATGGATTGCGACATTGAGGTGAC
25. DQ341364.1/Singapore/5511-SIN-00 (B5)	ATGGATTGCGACATTGAGGTGAC
26. JN992282.1/Brunei/BRU/2006/35334 (B5)	ATGGATTGCGACATTGAGGTGAC
27. FJ357378.1/Taiwan/N2838-TW03/B5/2003 (B5)	ATGGATTGCGACATTGAGGTGAC
28. DQ341363.1/Malaysia/S19841-SAR-03/B5/2003 (B5)	ATGGATTGCGACATTGAGGTGAC
29. DQ341362.1/Malaysia/SB12736-SAR-03/B4/2003 (B4)	ATGGATTGCGACATTGAGGTGAC
30. JN964686.1/China/Fujian/EV71/Xiamen/2009/B5/2009 (B5)	ATGGACTGCGACATTGAAGTGAC
31. GQ231942.1/Taiwan/TW/96016/08/B5 (B5)	ATGGATTGCGACATTGAGGTGAC
32. GQ231925.1/Taiwan/TW/1101/08/B5 (B5)	ATGGATTGCGACATTGAGGTGAC
33. GQ231934.1/Taiwan/TW/70811/08/B5 (B5)	ATGGATTGCGACATTGAGGTGAC
34. GQ231943.1/Taiwan/TW/96022/08/B5 (B5)	ATGGATTGCGACATTGAGGTGAC
35. GQ231935.1/Taiwan//TW/70886/08/B5 (B5)	ATGGATTGCGACATTGAGGTGAC
36. GQ231941.1/Taiwan/TW/96002/08/B5 (B5)	ATGGATTGCGACATTGAGGTGAC
37. EU527985.1/Taiwan/2007-08747/2007/B5/2007 (B5)	ATGGATTGCGACATTGAGGTGAC
38. FJ357385.1/Taiwan/M0380-TW08/B5/2008 (B5)	ATGGATTGCGACATTGAGGTGAC
39. HM622390.1/Taiwan/2009-03531/B5/2009 (B5)	ATGGATTGCGACATTGAGGTGAC
40. GQ231936.1/Taiwan/TW/70902/08/B5 (B5)	ATGGATTGCGACATTGAGGTGAC
41. AB575927.1/Netherlands/11316/B2/1986 (B2)	ATGGATTGCGATATTGAGGTAAC
42. AB575928.1/Netherlands/11590/B2/1986 (B2)	ATGGATTGCGATATTGAGGTAAC
43. AB575923.1/Netherlands/20233/B2/1983 (B2)	ATGGATTGTGATATTGAGGTGAC
44. U22522.1/USA/MS/7423/87/B2/1987 (B2)	ATGGATTGTGATATTGAGGTAAC
45. FJ357384.1/Taiwan/266-TW86/B1/1986 (B1)	ATGGATTGTGATATTGAAGTGAC
46. FJ357383.1/Taiwan/252-TW86/B1/1986 (B1)	ATGGATTGTGATATTGAAGTGAC
47. FJ357380.1/Taiwan/237-TW86/B1/1986 (B1)	ATGGATTGTGATATTGAAGTGAC
48. FJ357379.1/Taiwan/236-TW86/B1/1986 (B1)	ATGGATTGTGATATTGAAGTGAC
49. FJ357382.1/Taiwan/238-TW86/B1/1986 (B1)	ATGGATTGTGATATTGAAGTGAC
50. FJ357381.1/Taiwan/244-TW86/B1/1986 (B1)	ATGGATTGTGACATTGAAGTGAC
51. AB575914.1/Netherlands/17000/B1/1971 (B1)	ATGGATTGTGATATTGAAGTGAC
52. AB575913.1/Netherlands/11977/B1/1971 (B1)	ATGGATTGCGATATTGAAGTGAC
53. AB575918.1/Netherlands/20574/B1/1978 (B1)	ATGGATTGTGATATTGAAGTGAC
54. AB575917.1/Netherlands/10196/B1/1977 (B1)	ATGGATTGTGATATTGAAGTGAC
55. HQ189392.1/Hungary/HUN/1978/1978 (B1)	ATGGATTGTGATATTGAAGTGAC
56. AB575916.1/Netherlands/16173/B1/1976 (B1)	ATGGATTGTGATATTGAAGTGAC
57. AB575915.1/Netherlands/9443/B1/1974 (B1)	ATGGATTGTGATATTGAAGTGAC
58. AB482183.1/Japan/Nagoya/B1/1973 (B1)	ATGGATTGTGATATTGAAGTGAC
59. HQ423143.1/China/Yunnan/KM186/09/2009/C4a/2009 (C4)	ATGGATTGTGACATCGAAGTGAC
60. HQ423142.1/China/KMM/09/2009 (C4)	ATGGATTGTGACATTGAAGTGAC
61. JQ742001.1/China/Guangxi/AFP2001064/EV71/GX/CHN/2001/C4b/2001 (C4)	ATGGATTGTGACATTGAAGTGAC
62. EU131776.1/Taiwan/N3340-TW-02/C4b/2002 (C4)	ATGGATTGTGACATTGAAGTGAC
63. JX678885.1/China/Shanghai/SH-17/SH/CHN/2002/C4b/2002 (C4)	ATGGATTGTGACATTGAAGTGAC
64. JQ742002.1/China/AFP2001071/EV71/GX/CHN/2001/C4b/2001 (C4)	ATGGATTGTGACATTGAAGTGAC
65. GU350629.1/China/AnHui/Fuyang-0805a (C4)	ATGGATTGTGACATTGAAGTGAC
66. FJ194964.1/China/Guangdong/EV71/GDFS/3/2008/C4b/2008 (C4)	ATGGATTGTGACATTGAAGTGAC
67. GQ994989.1/China/Chongqing/Chongqing1-09-China/2009/C4b/2009 (C4)	ATGGATTGTGACATTGAGGTGAC
68. HM807310.1/Taiwan/cmuh-050530-5/2005 (C4)	ATGGACTGCGATATAGAAGTGAC
69. AY465356.1/China/Shenzhen/SHZH03/C4b/2003 (C4)	ATGGATTGTGACATTGAAGTGAC
70. JX678874.1/China/Chongqing/CQ03-1/CQ/CHN/2003/C4b/2003 (C4)	ATGGATTGTGACATTGAAGTGAC
71. HQ647171.1/Canada/EV141-06/2006/C4a/2006 (C4)	ATGGATTGTGACATTGAAGTGAC
72. HQ647180.1/Canada/EV034-06/2006 (C4)	ATGGATTGTGACATTGAAGTGAC

	4,840 4,850
Consensus	ATGGACTGTGACATTGAAGTGAC
	FL19-20R
72. HQ647180.1/Canada/EV034-06/2006 (C4)	ATGGATTGTGACATTGAAGTGAC
73. HQ647179.1/Canada/EV144-06/2006 (C4)	ATGGATTGTGACATTGAAGTGAC
74. JX678886.1/China/Shanghai/SH-6/SH/CHN/2002/C4b/2002 (C4)	ATGGATTGTGACATTGAAGTGAC
75. AB550339.1/Japan/Labstrain/75-Yamagata-LPS1/C4/2003 (C4)	ATGGATTGTGACATTGAAGTGAC
76. AB550338.1/Japan/75-Yamagata-org/C4/2003 (C4)	ATGGATTGTGACATTGAAGTGAC
77. DQ133459.1/Taiwan/1235/C4/2004 (C4)	ATGGATTGTGACATTGAAGTGAC
78. DQ133458.1/Taiwan/984/C4/2004 (C4)	ATGGATTGTGACATTGAAGTGAC
79. FJ357373.1/Taiwan/S0584-TW04/C4/2004 (C4)	ATGGATTGTGACATTGAAGTGAC
80. EF373576.1/Taiwan/E2004104-TW-CDC (C4)	ATGGATTGTGACATTGAAGTGAC
81. GQ231938.1/TaiwanTW/71552/05 (C4)	ATGGATTGTGACATTGAAGTGAC
82. GQ231926.1/Taiwan/TW/1956/05/C4a/2005 (C4)	ATGGATTGTGACATTGAAGTGAC
83. GQ231940.1/Taiwan/TW/72232/04/C4 (C4)	ATGGATTGTGACATTGAAGTGAC
84. GQ231937.1/Taiwan/TW/71428/05/C4 (C4)	ATGGATTGTGACATTGAAGTGAC
85. GQ231939.1/Taiwan/TW/71595/04/C4 (C4)	ATGGATTGTGACATTGAAGTGAC
86. GQ231929.1/Taiwan/TW/2728/04/C4a/2004 (C4)	ATGGATTGTGACATTGAAGTGAC
87. FJ357374.1/Taiwan/N2121-TW05/C4/2005 (C4)	ATGGATTGTGACATTGAAGTGAC
88. GQ231931.1/Taiwan/TW/2824/04/C4 (C4)	ATGGATTGTGACATTGAAGTGAC
89. GQ231928.1/Taiwan/TW/2639/04/C4 (C4)	ATGGATTGTGACATTGAAGTGGC
90. GQ231927.1/Taiwan/TW/2429/04/C4 (C4)	ATGGATTGTGACATTGAAGTGAC
91. GQ231930.1/Taiwan/TW/2815/04/C4 (C4)	ATGGATTGTGACATTGAAGTGAC
92. JQ965759.1/Vietnam/540V/VNM/05/C4/2005 (C4)	ATGGATTGTGACATTGAAGTGAC
93. GQ231932.1/Taiwan/TW/2871/04/C4 (C4)	ATGGATTGTGACATTGAAGTGAC
94. GQ994992.1/China/Henan/Henan2-09-China/2009/C4a/2009 (C4)	ATGGATTGTGACATTGAAGTGAC
95. GQ279369.1/China/SZ/HK08-5/2008 (C4)	ATGGATTGTGACATTGAAGTGAC
96. JX244184.1/China/SDLY48/C/2009 (C4)	ATGGATTGTGACATTGAAGTGAC
97. FJ606447.1/China/Beijing/BJ08-Z004-3/2008/C4a/2008 (C4)	ATGGATTGTGACATTGAAGTGAC
98. JQ681218.1/China/Henan/H8-1/C4a/2008 (C4)	ATGGAGTGTGACATTGAAGTGAC
99. GU196833.1/China/Henan1-09-China/2009 (C4)	ATGGATTGTGACATTGAAGTGAC
100. HQ129932.1/China/BJ06-SJS06/06/C4/2006 (C4)	ATGGATTGCGACATTGAAGTGAC
101. FJ607336.1/China/28/SHENZHEN/08/China/HFMD/2008/2008 (C4)	ATGGACTGTGATATTGAAGTGAC
102. FJ828519.1/China/BJ08/C4/2008 (C4)	ATGGACTGTGACATTGAGGTGAC
103. FJ360545.1/China/GZ-08-02/C4/2008 (C4)	ATGGACTGTGACATTGAAGTGAC
104. JX244182.1/China/SDLY1/2009 (C4)	ATGGACTGTGACATTGAAGTGAC
105. JF820312.1/China/LCH01/C4/2010 (C4)	ATGGACTGTGACATTGAAGTGAC
106. HM002488.1/China/BJ366/2009 (C4)	ATGGACTGTGACATTGAAGTGAC
107. GQ231933.1/Taiwan/TW/70516/08/C4a/2008 (C4)	ATGGACTGTGACATTGAAGTGAC
108. KC109780.1/China/202/Jingdezhen/China/HFMDSevere/2011/C4/2011 (C4)	ATGGACTGTGACATTGAGGTGAC
109. HM002485.1/China/BJ972008 (C4)	ATGGACTGTGACATTGAAGTGAC
110. GU366191.1/China/Henan/Henan10-08-China/2008/C4a/2008 (C4)	ATGGACTGTGACATTGAAGTGAC
111. FJ713137.1/Shanghai/Shanghai-036-2009/C4a/2009 (C4)	ATGGACTGTGACATTGAAGTGAC
112. EU753384.2/China/Shandong/522-04T/SD/CHN/07/C4a/2007 (C4)	ATGGACTGTGACATTGAAGTGAC
113. HM003207.1/China/87-2008-Xi'an-Shaanxi/2008 (C4)	ATGGACTGTGACATTGAAGTGAC
114. EU753365.2/China/Shandong/518-03F/SD/CHN/07/C4a/2007 (C4)	ATGGACTGTGACATTGAAGTGAC
115. EU753407.2/China/Shandong/TC03F/SD/CHN/07/C4a/2007 (C4)	ATGGACTGTGACATTGAAGTGAC
116. GQ994988.1/China/Anhui/Anhui1-09-China/C4a/2009 (C4)	ATGGACTGTGACATTGAAGTGAC
117. JQ708210.1/China/isolateTS/C4/2009 (C4)	ATGGACTGTGACATTGAAGTGAC
118. HQ611148.1/China/AH08/06/C4/2008 (C4)	ATGGACTGTGACATTGAAGTGAC
119. EU753398.2/China/Shandong/523-07T/SD/CHN/07/2007 (C4)	ATGGACTGTGACATTGAAGTGAC
120. EU753397.2/China/Shandong/523-05T/SD/CHN/07/C4a/2007 (C4)	ATGGACTGTGACATTGAAGTGAC
121. JX678882.1/China/Shandong/SD07-1/SD/CHN/2007/C4a/2007 (C4)	ATGGACTGTGACATTGAAGTGAC
122. EU753375.2/China/Shandong/521-18S/SD/CHN/07/C4a/2007 (C4)	ATGGACTGTGACATTGAAGTGAC
123. HM002487.1/China/BJ303/2008 (C4)	ATGGACTGTGACATTGAGGTGAC
124. JX678878.1/China/Anhui/FY17.08-7/AH/CHN/2008/C4a/2008 (C4)	ATGGACTGTGACATTGAGGTGAC
125. JX025561.1/China/FY7VP5/AH/CHN/2008 (C4)	ATGGACTGTGACATTGAGGTGAC
126. JQ316638.1/China/HQ09231463/2011 (C4)	ATGGACTGTGACATTGAAGTGAC
127. HQ426649.1/China/Anhui/2010FJLY008/2010/C4a/2010 (C4)	ATGGACTGTGACATTGAAGTGAC
128. GQ994991.1/China/Chongqing3-09-China/2009 (C4)	ATGGACTGTGACATTGAAGTGAC
129. HM053670.1/China/strain-BJ393/2009 (C4)	ATGGACTGTGACATTGAAGTGAC
130. FJ360544.1/China/GZ-08-01/C4/2008 (C4)	ATGGACTGTGACATTGAAGTGAC
131. HQ998852.1/China/Henan/EV71/Henan/106/2009/C4a/2009 (C4)	ATGGACTGTGACATTGAAGTGAC
132. JX244187.1/China/SDLY153/2010 (C4)	ATGGACTGTGACATTGAAGTAAC
133. GQ994990.1China/Chongqing2-09-China/C4/2009 (C4)	ATGGACTGTGACATTGAAGTGAC
134. EU864507.1/China/Zhejiang/EV71/Zhejiang08/2008/C4a/2008 (C4)	ATGGACTGTGACATTGAAGTGAC
135. FJ194965.1/China/EV71/GDSG/17/2008/C/2008 (C4)	ATGGACTGTGACATTGAAGTGAC
136. FJ607334.1/China/SHENZHEN/08/China/HFMD/2008/2008 (C4)	ATGGACTGTGATATTGAAGTGAC
137. GQ279370.1/China/SZ/HK08-6/2008 (C4)	ATGGACTGTGATATTGAAGTGAC
138. FJ607335.1/China/4/SHENZHEN/08/China/HFMD/2008/2008 (C4)	ATGGACTGTGATATTGAAGTGAC
139. JQ736684.2/China/SHAPHC695F/SH/CHN/10/C4a/2010 (C4)	ATGGACTGTGACATTGAAGTGAC
140. JQ074187.1/China/Jinan1002/2010 (C4)	ATGGACTGTGACATTGAAGTGAC
141. JQ074189.1/China/Jinan1005/2010 (C4)	ATGGACTGTGACATTGAAGTGAC
142. JQ074188.1/China/EV71/Jinan1004/2010 (C4)	ATGGACTGTGACATTGAAGTGAC
143. JF830007.1/China/Zhejiang/EV71/Ningbo.CHN/065/2010/C4a/2010 (C4)	ATGGACTGTGACATTGAGGTGAC

	4,840 ... 4,850
Consensus	ATGGACTGTGACATTGAAGTGAC
	FL19-20R
142. JQ074188.1/China/EV71/Jinan1004/2010 (C4)	ATGGACTGTGACATTGAAGTGAC
143. JF830007.1/China/Zhejiang/EV71/Ningbo.CHN/065/2010/C4a/2010 (C4)	ATGGACTGTGACATTGAGGTGAC
144. JX111893.1/China/GD-DG2011-9/C4/2011 (C4)	ATGGACTGTGACATTGAAGTGAC
145. JX111891.1/China/GD-DG2011-5/C4/2011 (C4)	ATGGACTGTGACATTGAAGTGAC
146. JX111889.1/China/GD-DG2011-2/C4/2011 (C4)	ATGGACTGTGACATTGAAGTGAC
147. JX111888.1/China/GD-DG2011-1/C4/2011 (C4)	ATGGACTGTGACATTGAAGTGAC
148. JX111892.1/China/GD-DG2011-8/C4/2011 (C4)	ATGGACTGTGACATTGAAGTGAC
149. JX111890.1/China/GD-DG2011-3/C4/2011 (C4)	ATGGACTGTGACATTGAAGTGAC
150. JQ806378.1/China/Jiangxi/35/Jingdezhen/China/HFMDSevere/2011/C4a/2011 (C4)	ATGGACTGTGATATTGAAGTGAC
151. JX017384.1/China/01011Y/2011 (C4)	ATGGACTGTGACATTGAAGTGAC
152. HQ891925.1/China/Shanghai-36-2009/C4/2009 (C4)	ATGGACTGTGACATTGAAGTGAC
153. JX244186.1/China/SDLY107/2010 (C4)	ATGGACTGTGACATTGAAGTGAC
154. HQ850973.1/China/003/XM/2010/2010 (C4)	ATGGACTGTGACATTGAAGTGAC
155. FJ360546.1/China/GZ-08-03/C4/2008 (C4)	ATGGACTGTGACATTGAAGTGAC
156. HQ891926.1/China/Shanghai-51-2009/C4/2009 (C4)	ATGGACTGTGACATTGAAGTGAC
157. GQ892830.1/China/GX/LZ (C4)	ATGGACTGTGACATTGAAGTGAC
158. JN020147.1/China/Henan/Luoyang/2011/C4a/2011 (C4)	ATGGACTGTGACATTGAAGTGAC
159. JQ517316.1/China/Henan/Kaifeng/2010/C4a/2010 (C4)	ATGGACTGTGACATTGAAGTGAC
160. JN052925.1/China/Henan/Nanyang/2011-China/2011/C4a/2011 (C4)	ATGGACTGTGACATTGAAGTGAC
161. HQ828086.1/China/Zhejiang/NBChina01/2010/C4a/2010 (C4)	ATGGACTGTGACATTGAAGTGAC
162. JX986739.1/China/Wuhan1143/HuB/CHN/2011/C4/2011 (C4)	ATGGACTGTGACATTGAAGTGAC
163. HQ407557.1/China/Liangning/LN009/2010/C4a/2010 (C4)	ATGGACTGTGACATTGAAGTGAC
164. JQ074190.1/China/Shandong/Jinan1006/2010/C4a/2010 (C4)	ATGGACTGTGACATTGAAGTGAC
165. JN001860.1/China/Zhejiang/NB/2010/01/C4a/2010 (C4)	ATGGACTGTGACATTGAAGTGAC
166. JQ804832.1/China/EV71/Hubei-XF/CHN/2010/C4/2010 (C4)	ATGGACTGTGACATTGAAGTGAC
167. JQ514785.1/China/BC08/C4a/2008 (C4)	ATGGACTGTGACATTGAAGTGAC
168. HM053669.1/China/BJ293/2008 (C4)	ATGGACTGTGACATTGAAGTGAC
169. HM002484.1/China/BJ67/2008 (C4)	ATGGACTGTGACATTGAAGTGAC
170. GU396280.1/China/Gansu/EV71/Lanzhou01/GS/CHN/09/C4a/2009 (C4)	ATGGACTGTGACATTGAAGTGAC
171. HQ400942.1/China/HZ08/Hangzhou/2008/C4/2008 (C4)	ATGGACTGTGACATTGAAGTGAC
172. HQ891929.1/China/Shanghai-118-2009/C4/2009 (C4)	ATGGACTGTGACATTGAAGTGAC
173. FJ607338.1/China/Shenzhen/605/SHENZHEN/08/China/HFMD/C4a/2008 (C4)	ATGGACTGTGACATTGAAGTGAC
174. JX986737.1/China/Wuhan1042/HuB/CHN/2011/C4/2011 (C4)	ATGGATTGTGACATTGAAGTGAC
175. HQ891927.1/China/Shanghai-64-2009/C4/2009 (C4)	ATGGACTGTGACATTGAAGTGAC
176. HQ891928.1/China/Shanghai-117-2009/C4/2009 (C4)	ATGGACTGTGACATTGAAGTGAC
177. HQ891924.1/China/Shanghai-28-2009/C4/2009 (C4)	ATGGACTGTGACATTGAAGTGAC
178. HQ891923.1/China/Shanghai/Shanghai-27-2009/C4a/2009 (C4)	ATGGACTGTGACATTGAAGTGAC
179. HM002489.1/China/Beijing/Beijing398/2009/C4a/2009 (C4)	ATGGACTGTGACATTGAAGTGAC
180. JN256063.1/China/RDcells/M186-1179F/HeN/CHN/2009/C4a (C4)	ATGGACTGTGACATTGAAGTGAC
181. JN256062.1/China/RDcells/M183-1176F/HeN/CHN/2009/C4a (C4)	ATGGACTGTGACATTGAAGTGAC
182. JN256064.1/China/Henan/RDcells/M188-1181F/HeN/CHN/2009/C4a/2009 (C4)	ATGGACTGTGACATTGAAGTGAC
183. JX678881.1/China/Henan/HeN09-17/HeN/CHN/2009/C4a/2009 (C4)	ATGGACTGTGACATTGAAGTGAC
184. JX678880.1/China/Henan/HeN09-1/HeN/CHN/2009/C4a/2009 (C4)	ATGGACTGTGACATTGAAGTGAC
185. EU812515.1/China/Anhui/FY23/2008/C4a/2008 (C4)	ATGGACTGTGACATTGAAGTGAC
186. GU459071.1/China/FY23-K14/C4/2008 (C4)	ATGGACTGTGACATTGAAGTGAC
187. GU459070.1/China/FY23-K12/C4/2008 (C4)	ATGGACTGTGACATTGAAGTGAC
188. JX244185.1/China/SDLY96/2009 (C4)	ATGGACTGTGACATTGAAGTGAC
189. JX244183.1/China/SDLY11/2009 (C4)	ATGGACTGTGACATTGAAGTGAC
190. JN256061.1/China/RDcells/G398-1037F/HeN/CHN/2009/C4a (C4)	ATGGACTGTGACATTGAAGTGAC
191. HQ825317.1/China/Shandong/EV71/JN200804/2008/C4a/2008 (C4)	ATGGACTGTGACATTGAAATGAC
192. JF913464.1/China/Shandong/EV71/JN200803/2008/C4a/2008 (C4)	ATGGACTGTGACATTGAAGTGAC
193. JN256060.1/China/RDcellsG333-972F/HeN/CHN/2009/C4a (C4)	ATGGACTGTGACATTGAAGTGAC
194. JN256059.1/China/Henan/RDcells/G288-927F/HeN/CHN/2009/C4a/2009 (C4)	ATGGACTGTGACATTGAAGTGAC
195. JX678884.1/China/Shandong/SD09-21/SD/CHN/2009/C4a/2009 (C4)	ATGGACTGTGACATTGAAGTGAC
196. JX678883.1/China/Shandong/SD09-14/SD/CHN/2009/C4a/2009 (C4)	ATGGACTGTGACATTGAAGTGAC
197. JQ319054.1/China/Beijing/BJ09/07/C4a/2009 (C4)	ATGGACTGTGACATTGAAGTGAC
198. JF820315.1/China/AH01/C4/2010 (C4)	ATGGACTGTGACATTGAAGTGAC
199. JF820314.1/China/BJ02/C4/2010 (C4)	ATGGACTGTGACATTGAAGTGAC
200. JQ708209.1/China/isolateBJ/C4/2010 (C4)	ATGGACTGTGACATTGAAGTGAC
201. JF820316.1/China/BJ01/C4/2010 (C4)	ATGGACTGTGACATTGAAGTGAC
202. JF820313.1/China/LCH02/C4/2010 (C4)	ATGGACTGTGACATTGAAGTGAC
203. FJ606449.1/China/BJ08-Z020-1/2008 (C4)	ATGGACTGTGACATTGAAGTGAC
204. GU198371.1/China/FY08-C30-P14/2008 (C4)	ATGGACTGTGACATTGAAGTGAC
205. GU198370.1/China/C2/FY08-C30/2008 (C4)	ATGGACTGTGACATTGAAGTGAC
206. GU198367.1/China/Anhui/FY08-C30-P2/2008/C4a/2008 (C4)	ATGGACTGTGACATTGAAGTGAC
207. GU198369.1/China/C1/FY08-C30-P11/2008 (C4)	ATGGACTGTGACATTGAAGTGAC
208. GU198368.1/China/C1/FY08-C30-P9/2008 (C4)	ATGGACTGTGACATTGAAGTGAC
209. FJ439769.1/China/Anhui/Fuyang-0805/2008/C4a/2008 (C4)	ATGGACTGTGACATTGAAGTGAC
210. EU703814.1/China/Anhui/2008/EV71/Fuyang.Anhui.P.R.C/17.08/3/2008/C4a/2008 (C4)	ATGGACTGTGACATTGAAGTGAC
211. EU703812.1/China/Anhui/EV71/Fuyang.Anhui.P.R.C/17.08/1/2008/C4a/2008 (C4)	ATGGACTGTGACATTGAAGTGAC
212. JX678875.1/China/Anhui/FY17.08-4/AH/CHN/2008/C4a/2008 (C4)	ATGGACTGTGACATTGAAGTGAC
213. FJ158601.1/China/DTID/ZJU-74/2008 (C4)	ATGGACTGTGACATTGAAGTGAC
214. FJ158600.1/China/Zhejiang/DTID/ZJU-62/2008/C4a/2008 (C4)	ATGGACTGTGACATTGAAGTGAC

	4,840 / 4,850
Consensus	ATGGACTGTGACATTGAAGTGAC
	FL19-20R
214. FJ158600.1/China/Zhejiang/DTID/ZJU-62/2008/C4a/2008 (C4)	ATGGACTGTGACATTGAAGTGAC
215. FJ606448.1/China/BJ08-Z011-4/C4/2008 (C4)	ATGGACTGTGACATTGAAGTGAC
216. EU703813.1/China/Anhui/EV71/Fuyang.Anhui.P.R.C/17.08/2/2008/C4a/2008 (C4)	ATGGACTGTGACATTGAAGTGAC
217. JX678879.1/China/Anhui/FY17.08-8/AH/CHN/2008/C4a/2008 (C4)	ATGGACTGTGACATTGAAGTGAC
218. JX678877.1/China/Anhui/FY17.08-6/AH/CHN/2008/C4a/2008 (C4)	ATGGACTGTGACATTGAAGTGAC
219. JX678876.1/China/Anhui/FY17.08-5/AH/CHN/2008/C4a/2008 (C4)	ATGGACTGTGACATTGAAGTGAC
220. JF799986.1/China/LabstrainGuangdong/2009 (C4)	ATGGATTGTGACATTGAAGTGAC
221. JF738002.1/Thailand/THA-EV71-044/C4/2009 (C4)	ATGGATTGTGACATTGAAGTGAC
222. FJ607337.1/China/Shenzhen/121/SHENZHEN/08/China/HFMD-Fatal/2008/C4a/2008 (C4)	ATGGATTGTGACATTGAAGTGAC
223. FJ606450.1/China/BJ08-Z025-5/2008 (C4)	ATGGATTGTGACATTGAAGTGAC
224. HM622391.1/Taiwan/2008-00643/C2-like/2008 (C2)	ATGGACTGTGACATTGAGGTGAC
225. JQ280307.1/Taiwan/3149/C2/2008 (C2)	ATGGACTGTGACATTGAGGTGAC
226. HM622392.1/Taiwan/2008-07776/C2-like/2008 (C2)	ATGGACTGTGACATTGAGGTGAC
227. EU527983.1/Taiwan/2007-07364/2007/C5/2007 (C5)	ATGGATTGCGACATTGAGGTGAC
228. EF063152.1/Taiwan/E2005125-TW (C5)	ATGGATTGCGACATTGAGGTGAC
229. DQ341355.1/SouthKorea/06-KOR-00/C3/2000 (C3)	ATGGACTGTGATATTGAAGTGAC
230. DQ341356.1/SouthKorea/03-KOR-00/C3/2000 (C3)	ATGGACTGTGATATTGAAGTGAC
231. AF119795.2/Taiwan/TW/2272/98/C/1998 (UNKNOWN)	ATGGACTGCGACATTGAGGGGAC
232. AF119796.3/Taiwan/TW/2086/98/C2/1998 (C2)	ATGGACTGCGACATTGAGGGGAC
233. DQ060149.1/Taiwan/pinf7-54A/C2/2005 (C2)	ATGGACTGCGAAATTGAAGTGAC
234. AF304457.1/Taiwan/5746/98/C2/1998 (C2)	ATGGACTGCGATATTGAGGTGAC
235. AF176044.1/Taiwan/1245a/98/tw (C2)	ATGGACTGCGATATTGAGGTGAC
236. JN544418.1/China/4643-TW98/C2/1998 (C2)	ATGGACTGCGATATTGAGGTGAC
237. AF304458.1/Taiwan/Tainan/4643/98/C2 (C2)	ATGGACTGCGATATTGAGGTGAC
238. AF304459.1/Taiwan/Tainan/6092/98/C2 (C2)	ATGGACTGCGATATTGAGGTGAC
239. N544419.1/China/mousestrain/MP4/C2/2004 (C2)	ATGGACTGCGATATTGAGGTGAC
240. AF136379.1/NCKU9822 (C2)	ATGGACTGCGATATTGAGGTGAC
241. AB550333.1/Japan/Labstrain/1095-LPS1/C2/1997 (C2)	ATGGACTGCGATATTGAGGTGAC
242. AB550332.1/Japan/1095-org/C2/1997 (C2)	ATGGACTGCGATATTGAGGTGAC
243. JN992283.1/Australia/0964/SYD/98 (C2)	ATGGACTGCGATATTGAGGTGAC
244. JX025559.1/Australia/Labstrain/LAZ60-TR.C2/2010 (C2)	ATGGACTGTGATATTGAGGTAAC
245. DQ381846.1/Australia/6F/AUS/6/99/C2/1999 (C2)	ATGGACTGTGATATTGAGGTAAC
246. DQ341357.1/Australia/7F-AUS-6-99 (C2)	ATGGACTGCGATATTGAGGTAAC
247. AB575939.1/Netherlands/365/C2/2000 (C2)	ATGGATTGCGATATTGAGGTAAC
248. HQ647173.1/Canada/EV003-07/2006 (C2)	ATGGACTGCGATATTGAGGTAAC
249. HQ647168.1/Canada/EV92-07/2007 (C2)	ATGGACTGCGATATTGAGGTAAC
250. HQ647174.1/Cana/EV123-07/2007 (C2)	ATGGACTGCGATATTGAGGTAAC
251. HQ647170.1/Canada/EV124-07/2007 (C2)	ATGGACTGCGATATTGAGGTAAC
252. HQ647176.1/Canada/EV049-07/2007 (C2)	ATGGACTGCGATATTGAGGTAAC
253. HQ647169.1/Canada/EV090-07/2007 (C2)	ATGGACTGCGATATTGAGGTAAC
254. HQ647178.1/Canada/EV082-07/2007 (C2)	ATGGACTGCGATATTGAGGTAAC
255. HQ647177.1/Canada/EV073-07/2007 (C2)	ATGGACTGCGATATTGAGGTAAC
256. HQ647175.1/Canada/EV053-07/2007 (C2)	ATGGACTGTGACATTGAGGTAAC
257. HQ647167.1/Canda/EV054-07/2007 (C2)	ATGGACTGTGACATTGAGGTAAC
258. FJ172159.1/Singapore/NUH0075/SIN/08/2008 (C2)	ATGGACTGTGACATTGAGGTAAC
259. AB575942.1/Netherlands/3692/C2/2007 (C2)	ATGGACTGTGACATTGAGGTAAC
260. AB575941.1/Netherlands/2485/C2/2007 (C2)	ATGGACTGTGACATTGAGGTAAC
261. JN835312.1/France//MRS/09/3663/C3/2009 (C3)	ATGGACTGTGACATTGAGGTAAC
262. AB575948.1/Netherlands/10118/C2/2010 (C2)	ATGGACTGTGACATTGAGGTAAC
263. AB575938.1/Netherlands/10098/C1/2010 (C1)	ATGGACTGCGATATTGAAGTGAC
264. JF738000.1/Thailand/THA-EV71-002/C1/2009 (C1)	ATGGACTGCGATATTGAAGTGAC
265. EU414335.2/Switzerland/PLday1-CH-06/C1 (C1)	ATGGACTGCGATATTGAAGTGAC
266. EU414334.2/Switzerland/PLday4-CH-06/C1 (C1)	ATGGACTGCGATATTGAAGTGAC
267. EU414333.2/Switzerland/CSF-CH-06/C1 (C1)	ATGGACTGCGATATTGAAGTGAC
268. EU414331.2/Switzerland/LR-CH-06/C1 (C1)	ATGGACTGCGATATTGAAGTGAC
269. DQ341360.1/Malaysia/J115-MAL-01 (C1)	ATGGACTGCGATATTGAAGTGAC
270. DQ341358.1/Malaysia/S40221-SAR-00/C1/2000 (C1)	ATGGACTGCGATATCGAAGTGAC
271. AB550341.1/Malaysia/Labstrain/KED005-LPS2/C1/1997 (C1)	ATGGACTGCGATATTGAAGTGAC
272. AB550340.1/Malaysia/KED005-org/C1/1997 (C1)	ATGGACTGCGATATTGAAGTGAC
273. DQ452074.1/Norway/804/NO/03/C1/2003 (C1)	ATGGACTGCGATATTGAAGTGAC
274. DQ341361.1/Australia/1M-AUS-12-00/C1 (C1)	ATGGACTGCGATATTGAAGTGAC
275. AB575937.1/Netherlands/1416/C1/2001 (C1)	ATGGACTGCGATATTGAAGTGAC
276. DQ341359.1/Malaysia/S10862-SAR-98/C1/1998 (C1)	ATGGACTGCGACATTGAAGTGAC
277. HQ647172.1/Canada/EV063-04/1994 (C1)	ATGGACTGCGATATTGAAGTGAC
278. AB575936.1/Netherlands/9612/C1/1991 (C1)	ATGGACTGCGATATTGAAGTGAC
279. AB575935.1/Netherlands/480/C1/1991 (C1)	ATGGACTGCGATATTGAAGTGAC

	50 … 4,460 … 4,470
Consensus	CAAACACCGAATTGAACCTGT
	FL10F; Primer Bind
1. GU434678.1/China/Hubei/EV71-Hubei-09-China/B5/2009 (B5)	CAAACACCGTATTGAACCTGT
2. AB204853.1/BrCr-ts/A (A)	CAAACACCGTATTGAACCTGT
3. AB575911.1/Netherlands/10076/B0/1966 (B0)	CAAACACCGTATTGAACCTGT
4. AB575912.1/Netherlands/10857/B0/1966 (B0)	CAAACACCGTATTGAACCTGT
5. DQ341354.1/Singapore/3799-SIN-98/B3/1998 (B3)	CAAACACCGAATTGAACCTGT
6. DQ341368.1/Malaysia/MY104-9-SAR-97/B3 (B3)	CAAACACCGAATTGAACCTGT
7. DQ341367.1/Malaysia/MY821-3-SAR-97/B3/1997 (B3)	CAAACACCGAATTGAACCTGT
8. AB550335.1/Malaysia/Labstrain/SK-EV006-LPS1/B3/1997 (B3)	CAAACACCGAATTGAACCTGT
9. AB550334.1/Malaysia/SK-EV006-org/B3/1997 (B3)	CAAACACCGAATTGAACCTGT
10. AB469182.1/Malaysia/SK-EV006/Malaysia/97 (B3)	CAAACACCGAATTGAACCTGT
11. JQ950555.1/Australia26M/AUS/4/99/GuaR1/B3/2010 (B3)	CAAACACCGAATTGAACCTGT
12. EU364841.1/Australia/26M/AUS/4/99/B3/1999 (B3)	CAAACACCGAATTGAACCTGT
13. EF373575.1/Taiwan/E2002042-TW-CDC (B4)	CAAACACCGTATTGAACCTGT
14. FJ357377.1/Taiwan/S0296-TW00/B4/2000 (B4)	CAAACACCGTATTGAACCTGT
15. FJ357376.1/Taiwan/S0318-TW01/B4/2001 (B4)	CAAACACCGTATTGAACCTGT
16. DQ341366.1/Malaysia/SB2864-SAR-00/B4/2000 (B4)	CAAACACCGTATTGAACCTGT
17. DQ341365.1/Malaysia/PP37-MAL-01/B4/2001 (B4)	CAAACACCGTATTGAACCTGT
18. AF316321.2/Singapore/5865/sin/000009/B4 (B4)	CAAACACCGTATTGAACCTGT
19. AF352027.1/Singapore/5666/sin/002209/B4 (B4)	CAAACACCGTATTGAACCTGT
20. FJ357375.1/Taiwan/N7008-TW99/B4/1999 (B4)	CAAACACCGTATTGAACCTGT
21. AB550337.1/Japan/Labstrain/C7/Osaka-LPS1/B4/1997 (B4)	CAAACACCGTATTGAACCTGT
22. AB550336.1/Japan/C7/Osaka-org/B4/1997 (B4)	CAAACACCGTATTGAACCTGT
23. FJ461781.1/Singapore/NUH0083/SIN/08/B5/2008 (B5)	CAAACACCGTATTGAACCTGT
24. JF738001.1/Thailand/THA-EV71-019/B5/2009 (B5)	CAAACACCGTATTGAACCTGT
25. DQ341364.1/Singapore/5511-SIN-00 (B5)	CAAACACCGTATTGAACCTGT
26. JN992282.1/Brunei/BRU/2006/35334 (B5)	CAAACACCGTATTGAACCTGT
27. FJ357378.1/Taiwan/N2838-TW03/B5/2003 (B5)	CAAACACCGTATTGAACCTGT
28. DQ341363.1/Malaysia/S19841-SAR-03/B5/2003 (B5)	CAAACACCGTATTGAACCTGT
29. DQ341362.1/Malaysia/SB12736-SAR-03/B4/2003 (B4)	CAAACACCGTATTGAACCTGT
30. JN964686.1/China/Fujian/EV71/Xiamen/2009/B5/2009 (B5)	CAAACACCGTATTGAACCTGT
31. GQ231942.1/Taiwan/TW/96016/08/B5 (B5)	CAAACACCGTATTGAACCTGT
32. GQ231925.1/Taiwan/TW/1101/08/B5 (B5)	CAAACACCGTATTGAACCTGT
33. GQ231934.1/Taiwan/TW/70811/08/B5 (B5)	CAAACACCGTATTGAACCTGT
34. GQ231943.1/Taiwan/TW/96022/08/B5 (B5)	CAAACACCGTATTGAACCTGT
35. GQ231935.1/Taiwan//TW/70886/08/B5 (B5)	CAAACACCGTATTGAACCTGT
36. GQ231941.1/Taiwan/TW/96002/08/B5 (B5)	CAAACACCGTATTGAACCTGT
37. EU527985.1/Taiwan/2007-08747/2007/B5/2007 (B5)	CAAACACCGTATTGAACCTGT
38. FJ357385.1/Taiwan/M0380-TW08/B5/2008 (B5)	CAAACACCGTATTGAACCTGT
39. HM622390.1/Taiwan/2009-03531/B5/2009 (B5)	CAAACACCGTATTGAACCTGT
40. GQ231936.1/Taiwan/TW/70902/08/B5 (B5)	CAAACACCGTATTGAACCTGT
41. AB575927.1/Netherlands/11316/B2/1986 (B2)	CAAACACCGTATTGAACCTGT
42. AB575928.1/Netherlands/11590/B2/1986 (B2)	CAAACACCGTATTGAACCTGT
43. AB575923.1/Netherlands/20233/B2/1983 (B2)	CAAACACCGTATTGAACCTGT
44. U22522.1/USA/MS/7423/87/B2/1987 (B2)	CACACACCGTATTGAACCTGT
45. FJ357384.1/Taiwan/266-TW86/B1/1986 (B1)	CAAACACCGTATTGAACCTGT
46. FJ357383.1/Taiwan/252-TW86/B1/1986 (B1)	CAAACACCGTATTGAACCTGT
47. FJ357380.1/Taiwan/237-TW86/B1/1986 (B1)	CAAACACCGTATTGAACCTGT
48. FJ357379.1/Taiwan/236-TW86/B1/1986 (B1)	CAAACACCGTATTGAACCTGT
49. FJ357382.1/Taiwan/238-TW86/B1/1986 (B1)	CAAACACCGTATTGAACCTGT
50. FJ357381.1/Taiwan/244-TW86/B1/1986 (B1)	CAAACACCGTATTGAACCTGT
51. AB575914.1/Netherlands/17000/B1/1971 (B1)	CAAACACCGTATTGAACCTGT
52. AB575913.1/Netherlands/11977/B1/1971 (B1)	CAAACACCGTATTGAACCTGT
53. AB575918.1/Netherlands/20574/B1/1978 (B1)	CAAACACCGTATTGAACCTGT
54. AB575917.1/Netherlands/10196/B1/1977 (B1)	CAAACACCGTATTGAACCTGT
55. HQ189392.1/Hungary/HUN/1978/1978 (B1)	CAAACACCGTATTGAACCTGT
56. AB575916.1/Netherlands/16173/B1/1976 (B1)	CAAACACCGTATTGAACCTGT
57. AB575915.1/Netherlands/9443/B1/1974 (B1)	CAAACACCGTATTGAACCTGT
58. AB482183.1/Japan/Nagoya/B1/1973 (B1)	CAAACACCGTATTGAACCTGT
59. HQ423143.1/China/Yunnan/KM186/09/2009/C4a/2009 (C4)	CAAACACCGAATTGAACCTGT
60. HQ423142.1/China/KMM/09/2009 (C4)	CAAACACCGAATTGAACCTGT
61. JQ742001.1/China/Guangxi/AFP2001064/EV71/GX/CHN/2001/C4b/2001 (C4)	CAAACACCGAATTGAACCTGT
62. EU131776.1/Taiwan/N3340-TW-02/C4b/2002 (C4)	CAAACACCGAATTGAACCTGT
63. JX678885.1/China/Shanghai/SH-17/SH/CHN/2002/C4b/2002 (C4)	CAAACATCGAATTGAACCTGT
64. JQ742002.1/China/AFP2001071/EV71/GX/CHN/2001/C4b/2001 (C4)	CAAACACCGAATTGAACCTGT
65. GU350629.1/China/AnHui/Fuyang-0805a (C4)	CAAACACCGAATTGAACCTGT
66. FJ194964.1/China/Guangdong/EV71/GDFS/3/2008/C4b/2008 (C4)	CAAACACCGAATTGAACCTGT
67. GQ994989.1/China/Chongqing/Chongqing1-09-China/2009/C4b/2009 (C4)	CAAACACCGAATTGAACCTGT
68. HM807310.1/Taiwan/cmuh-050530-5/2005 (C4)	CAAACACCGAATTGAACCTGT
69. AY465356.1/China/Shenzhen/SHZH03/C4b/2003 (C4)	CAAGCACCGAATTGAACCTGT
70. JX678874.1/China/Chongqing/CQ03-1/CQ/CHN/2003/C4b/2003 (C4)	CAAACACCGAATTGAACCTGT
71. HQ647171.1/Canada/EV141-06/2006/C4a/2006 (C4)	CAAACACCGAATTGAACCTGT
72. HQ647180.1/Canada/EV034-06/2006 (C4)	CAAACACCGAATTGAACCTGT

50 4,460 4,470

Sequence	Alignment
Consensus	CAAACACCGAATTGAACCTGT
	FL10F; Primer Bind
72. HQ647180.1/Canada/EV034-06/2006 (C4)	CAAACACCGAATTGAACCTGT
73. HQ647179.1/Canada/EV144-06/2006 (C4)	CAAACACCGAATTGAACCTGT
74. JX678886.1/China/Shanghai/SH-6/SH/CHN/2002/C4b/2002 (C4)	CAAACACCGAATTGAACCTGT
75. AB550339.1/Japan/Labstrain/75-Yamagata-LPS1/C4/2003 (C4)	CAAACACCGAATTGAACCTGT
76. AB550338.1/Japan/75-Yamagata-org/C4/2003 (C4)	CAAACACCGAATTGAACCTGT
77. DQ133459.1/Taiwan/1235/C4/2004 (C4)	CAAACACCGAATTGAACCTGT
78. DQ133458.1/Taiwan/984/C4/2004 (C4)	CAAACACCGAATTGAACCTGT
79. FJ357373.1/Taiwan/S0584-TW04/C4/2004 (C4)	CAAACACCGAATTGAACCTGT
80. EF373576.1/Taiwan/E2004104-TW-CDC (C4)	CAAACACCGAATTGAACCTGT
81. GQ231938.1/TaiwanTW/71552/05 (C4)	CAAACACCGGATTGAACCTGT
82. GQ231926.1/Taiwan/TW/1956/05/C4a/2005 (C4)	CAAACATCGGATTGAACCTGT
83. GQ231940.1/Taiwan/TW/72232/04/C4 (C4)	CAAACACCGAATTGAACCTGT
84. GQ231937.1/Taiwan/TW/71428/05/C4 (C4)	CAAACACCGAATTGAACCTGT
85. GQ231939.1/Taiwan/TW/71595/04/C4 (C4)	CAAACACCGAATTGAACCTGT
86. GQ231929.1/Taiwan/TW/2728/04/C4a/2004 (C4)	CAAACACCGAATTGAACCTGT
87. FJ357374.1/Taiwan/N2121-TW05/C4/2005 (C4)	CAAACACCGAATTGAACCTGT
88. GQ231931.1/Taiwan/TW/2824/04/C4 (C4)	CAAACACCGAATTGAACCTGT
89. GQ231928.1/Taiwan/TW/2639/04/C4 (C4)	CAAACACCGAATTGAACCTGT
90. GQ231927.1/Taiwan/TW/2429/04/C4 (C4)	CAAACACCGAATTGAACCTGT
91. GQ231930.1/Taiwan/TW/2815/04/C4 (C4)	CAAACACCGAATTGAACCTGT
92. JQ965759.1/Vietnam/540V/VNM/05/C4/2005 (C4)	CAAACACCGAATTGAACCTGT
93. GQ231932.1/Taiwan/TW/2871/04/C4 (C4)	CAAACACCGAATTGAACCTGT
94. GQ994992.1/China/Henan/Henan2-09-China/2009/C4a/2009 (C4)	CAAACACCGAATTGAACCTGT
95. GQ279369.1/China/SZ/HK08-5/2008 (C4)	CAAACACCGAATTGAACCTGT
96. JX244184.1/China/SDLY48/C/2009 (C4)	CAAACACCGAATTGAACCTGT
97. FJ606447.1/China/Beijing/BJ08-Z004-3/2008/C4a/2008 (C4)	CAAACACCGAATTGAACCTGT
98. JQ681218.1/China/Henan/H8-1/C4a/2008 (C4)	CAAACACCGAATTGAACCTGT
99. GU196833.1/China/Henan1-09-China/2009 (C4)	CAAACACCGAATTGAACCTGT
100. HQ129932.1/China/BJ06-SJS06/06/C4/2006 (C4)	CAAACACCGAATTGAACCTGT
101. FJ607336.1/China/28/SHENZHEN/08/China/HFMD/2008/2008 (C4)	CAAACACCGAATTGAACCTGT
102. FJ828519.1/China/BJ08/C4/2008 (C4)	CAAACACCGAATTGAACCTGT
103. FJ360545.1/China/GZ-08-02/C4/2008 (C4)	CAAACACCGAATTGAACCTGT
104. JX244182.1/China/SDLY1/2009 (C4)	CAAACACCGAATTGAACCTGT
105. JF820312.1/China/LCH01/C4/2010 (C4)	CAAACACCGAATTGAACCTGT
106. HM002488.1/China/BJ366/2009 (C4)	CAAACACCGAATTGAACCTGT
107. GQ231933.1/Taiwan/TW/70516/08/C4a/2008 (C4)	CAAACACCGAATTGAACCTGT
108. KC109780.1/China/202/Jingdezhen/China/HFMDSevere/2011/C4/2011 (C4)	CAAACACCGAATTGAACCTGT
109. HM002485.1/China/BJ972008 (C4)	CAAACACCGAATTGAACCTGT
110. GU366191.1/China/Henan/Henan10-08-China/2008/C4a/2008 (C4)	CAAACACCGAATTGAACCTGT
111. FJ713137.1/Shanghai/Shanghai-036-2009/C4a/2009 (C4)	CAAACACCGAATTGAACCTGT
112. EU753384.2/China/Shandong/522-04T/SD/CHN/07/C4a/2007 (C4)	CAAACACCGAATTGAACCTGT
113. HM003207.1/China/87-2008-Xi'an-Shaanxi/2008 (C4)	CAAACATCGAATTGGACCTGT
114. EU753365.2/China/Shandong/518-03F/SD/CHN/07/C4a/2007 (C4)	CAAACACCGAATTGAACCTGT
115. EU753407.2/China/Shandong/TC03F/SD/CHN/07/C4a/2007 (C4)	CAAACACCGAATTGAACCTGT
116. GQ994988.1/China/Anhui/Anhui1-09-China/C4a/2009 (C4)	CAAACATCGAATTGAACCTGT
117. JQ708210.1/China/isolateTS/C4/2009 (C4)	CAAACATCGAATTGAACCTGT
118. HQ611148.1/China/AH08/06/C4/2008 (C4)	CAAACATCGAATTGAACCTGT
119. EU753398.2/China/Shandong/523-07T/SD/CHN/07/2007 (C4)	CAAACACCGGATTGAACCTGT
120. EU753397.2/China/Shandong/523-05T/SD/CHN/07/C4a/2007 (C4)	CAAACACCGGATTGAACCTGT
121. JX678882.1/China/Shandong/SD07-1/SD/CHN/2007/C4a/2007 (C4)	CAAACACCGGATTGAACCTGT
122. EU753375.2/China/Shandong/521-18S/SD/CHN/07/C4a/2007 (C4)	CAAACACCGGATTGAACCTGT
123. HM002487.1/China/BJ303/2008 (C4)	CAAACACCGGATTGAACCTGT
124. JX678878.1/China/Anhui/FY17.08-7/AH/CHN/2008/C4a/2008 (C4)	CAAACACCGGATTGAACCTGT
125. JX025561.1/China/FY7VP5/AH/CHN/2008 (C4)	CAAACACCGGATTGAACCTGT
126. JQ316638.1/China/HQ09231463/2011 (C4)	CAAACACCGAATTGAACCTGT
127. HQ426649.1/China/Anhui/2010FJLY008/2010/C4a/2010 (C4)	CAAACACCGAATTGAACCTGT
128. GQ994991.1/China/Chongqing3-09-China/2009 (C4)	CAAACACCGAATTGAACCTGT
129. HM053670.1/China/strain-BJ393/2009 (C4)	CAAACACCGAATTGAACCTGT
130. FJ360544.1/China/GZ-08-01/C4/2008 (C4)	CAAACACCGAATTGAACCTGT
131. HQ998852.1/China/Henan/EV71/Henan/106/2009/C4a/2009 (C4)	CAAACACCGAATTGAACCTGT
132. JX244187.1/China/SDLY153/2010 (C4)	CAAACACCGAATTGAACCTGT
133. GQ994990.1China/Chongqing2-09-China/C4/2009 (C4)	CAAACACCGAATTGAACCTGT
134. EU864507.1/China/Zhejiang/EV71/Zhejiang08/2008/C4a/2008 (C4)	CAAACACCGAATTGAACCTGT
135. FJ194965.1/China/EV71/GDSG/17/2008/C/2008 (C4)	CAAACACCGAATTGAACCTGT
136. FJ607334.1/China/SHENZHEN/08/China/HFMD/2008/2008 (C4)	CAAACACCGAATTGAACCTGT
137. GQ279370.1/China/SZ/HK08-6/2008 (C4)	CAAACACCGAATTGAACCTGT
138. FJ607335.1/China/4/SHENZHEN/08/China/HFMD/2008/2008 (C4)	CAAACACCGAATTGAACCTGT
139. JQ736684.2/China/SHAPHC695F/SH/CHN/10/C4a/2010 (C4)	CAAACACCGAATTGAACCTGT
140. JQ074187.1/China/Jinan1002/2010 (C4)	CAAACACCGAATTGAACCTGT
141. JQ074189.1/China/Jinan1005/2010 (C4)	CAAACACCGAATTGAACCTGT
142. JQ074188.1/China/EV71/Jinan1004/2010 (C4)	CAAACACCGAATTGAACCTGT
143. JF830007.1/China/Zhejiang/EV71/Ningbo.CHN/065/2010/C4a/2010 (C4)	CAAACACCGAATTGAACCTGT

	50 4,460 4,470
Consensus	CAAACACCGAATTGAACCTGT
	FL10F; Primer Bind
142. [illegible]	[illegible]
143. JF830007.1/China/Zhejiang/EV71/Ningbo.CHN/065/2010/C4a/2010 (C4)	CAAACACCGAATTGAACCTGT
144. JX111893.1/China/GD-DG2011-9/C4/2011 (C4)	CAAACACCGAATTGAACCTGT
145. JX111891.1/China/GD-DG2011-5/C4/2011 (C4)	CAAACACCGAATTGAACCTGT
146. JX111889.1/China/GD-DG2011-2/C4/2011 (C4)	CAAACACCGAATTGAACCTGT
147. JX111888.1/China/GD-DG2011-1/C4/2011 (C4)	CAAACACCGAATTGAACCTGT
148. JX111892.1/China/GD-DG2011-8/C4/2011 (C4)	CAAACACCGAATTGAACCTGT
149. JX111890.1/China/GD-DG2011-3/C4/2011 (C4)	CAAACACCGAATTGAACCTGT
150. JQ806378.1/China/Jiangxi/35/Jingdezhen/China/HFMDSevere/2011/C4a/2011 (C4)	CAAACACCGAATTGAACCTGT
151. JX017384.1/China/01011Y/2011 (C4)	CAAACACCGAATTGAACCTGT
152. HQ891925.1/China/Shanghai-36-2009/C4/2009 (C4)	CAAACACCGAATTGAACCTGT
153. JX244186.1/China/SDLY107/2010 (C4)	CAAACACCGAATTGAACCTGT
154. HQ850973.1/China/003/XM/2010/2010 (C4)	CAAACACCGAATTGAACCTGT
155. FJ360546.1/China/GZ-08-03/C4/2008 (C4)	CAAACACCGAATTGAACCTGT
156. HQ891926.1/China/Shanghai-51-2009/C4/2009 (C4)	CAAACACCGAATTGAACCTGT
157. GQ892830.1/China/GX/LZ (C4)	CAAACACCGTATTGAACCTGT
158. JN020147.1/China/Henan/Luoyang/2011/C4a/2011 (C4)	CAAACACCGAATTGAACCTGT
159. JQ517316.1/China/Henan/Kaifeng/2010/C4a/2010 (C4)	CAAACACCGAATTGAACCTGT
160. JN052925.1/China/Henan/Nanyang/2011-China/2011/C4a/2011 (C4)	CAAACACCGAATTGAACCTGT
161. HQ828086.1/China/Zhejiang/NBChina01/2010/C4a/2010 (C4)	CAAACACCGAATTGAACCTGT
162. JX986739.1/China/Wuhan1143/HuB/CHN/2011/C4/2011 (C4)	CAAACACCGAATTGAACCTGT
163. HQ407557.1/China/Liangning/LN009/2010/C4a/2010 (C4)	CAAACACCGAATTGAACCTGT
164. JQ074190.1/China/Shandong/Jinan1006/2010/C4a/2010 (C4)	CAAACACCGAATTGAACCTGT
165. JN001860.1/China/Zhejiang/NB/2010/01/C4a/2010 (C4)	CAAACACCGAATTGAACCTGT
166. JQ804832.1/China/EV71/Hubei-XF/CHN/2010/C4/2010 (C4)	CAAACACCGAATTGAACCTGT
167. JQ514785.1/China/BC08/C4a/2008 (C4)	CAAACACCGAATTGAACCTGT
168. HM053669.1/China/BJ293/2008 (C4)	CAAACACCGAATTGAACCTGT
169. HM002484.1/China/BJ67/2008 (C4)	CAAACACCGAATTGAACCTGT
170. GU396280.1/China/Gansu/EV71/Lanzhou01/GS/CHN/09/C4a/2009 (C4)	CAAACACCGAATTGAACCTGT
171. HQ400942.1/China/HZ08/Hangzhou/2008/C4/2008 (C4)	CAAACACCGAATTGAACCTGT
172. HQ891929.1/China/Shanghai-118-2009/C4/2009 (C4)	CAAACACCGAATTGAACCTGT
173. FJ607338.1/China/Shenzhen/605/SHENZHEN/08/China/HFMD/C4a/2008 (C4)	CAAACACCGAATTGAACCTGT
174. JX986737.1/China/Wuhan1042/HuB/CHN/2011/C4/2011 (C4)	CAAACACCGAATTGAACCTGT
175. HQ891927.1/China/Shanghai-64-2009/C4/2009 (C4)	CAAACACCGAATTGAACCTGT
176. HQ891928.1/China/Shanghai-117-2009/C4/2009 (C4)	CAAACACCGAATTGAACCTGT
177. HQ891924.1/China/Shanghai-28-2009/C4/2009 (C4)	CAAACACCGAATTGAACCTGT
178. HQ891923.1/China/Shanghai/Shanghai-27-2009/C4a/2009 (C4)	CAAACACCGAATTGAACCTGT
179. HM002489.1/China/Beijing/Beijing398/2009/C4a/2009 (C4)	CAAACACCGAATTGAACCTGT
180. JN256063.1/China/RDcells/M186-1179F/HeN/CHN/2009/C4a (C4)	CAAACACCGAATTGAACCTGT
181. JN256062.1/China/RDcells/M183-1176F/HeN/CHN/2009/C4a (C4)	CAAACACCGAATTGAACCTGT
182. JN256064.1/China/Henan/RDcells/M188-1181F/HeN/CHN/2009/C4a/2009 (C4)	CAAACACCGAATTGAACCTGT
183. JX678881.1/China/Henan/HeN09-17/HeN/CHN/2009/C4a/2009 (C4)	CAAACACCGAATTGAACCTGT
184. JX678880.1/China/Henan/HeN09-1/HeN/CHN/2009/C4a/2009 (C4)	CAAACACCGAATTGAACCTGT
185. EU812515.1/China/Anhui/FY23/2008/C4a/2008 (C4)	CAAACACCGAATTGAACCTGT
186. GU459071.1/China/FY23-K14/C4/2008 (C4)	CAAACACCGAATTGAACCTGT
187. GU459070.1/China/FY23-K12/C4/2008 (C4)	CAAACACCGAATTGAACCTGT
188. JX244185.1/China/SDLY96/2009 (C4)	CAAACACCGAATTGAACCTGT
189. JX244183.1/China/SDLY11/2009 (C4)	CAAACACCGAATTGAACCTGT
190. JN256061.1/China/RDcells/G398-1037F/HeN/CHN/2009/C4a (C4)	CAAACACCGAATTGAACCTGT
191. HQ825317.1/China/Shandong/EV71/JN200804/2008/C4a/2008 (C4)	CAAACACCGAATTGAACCTGT
192. JF913464.1/China/Shandong/EV71/JN200803/2008/C4a/2008 (C4)	CAAACACCGAATTGAACCTGT
193. JN256060.1/China/RDcellsG333-972F/HeN/CHN/2009/C4a (C4)	CAAACACCGAATTGAACCTGT
194. JN256059.1/China/Henan/RDcells/G288-927F/HeN/CHN/2009/C4a/2009 (C4)	CAAACACCGAATTGAACCTGT
195. JX678884.1/China/Shandong/SD09-21/SD/CHN/2009/C4a/2009 (C4)	CAAACACCGAATTGAACCTGT
196. JX678883.1/China/Shandong/SD09-14/SD/CHN/2009/C4a/2009 (C4)	CAAACACCGAATTGAACCTGT
197. JQ319054.1/China/Beijing/BJ09/07/C4a/2009 (C4)	CAAACACCGAATTGAACCTGT
198. JF820315.1/China/AH01/C4/2010 (C4)	CAAACACCGAATTGAACCTGT
199. JF820314.1/China/BJ02/C4/2010 (C4)	CAAACACCGAATTGAACCTGT
200. JQ708209.1/China/isolateBJ/C4/2010 (C4)	CAAACACCGAATTGAACCTGT
201. JF820316.1/China/BJ01/C4/2010 (C4)	CAAACACCGAATTGAACCTGT
202. JF820313.1/China/LCH02/C4/2010 (C4)	CAAACACCGAATTGAACCTGT
203. FJ606449.1/China/BJ08-Z020-1/2008 (C4)	CAAACACCGAATTGAACCTGT
204. GU198371.1/China/FY08-C30-P14/2008 (C4)	CAAACACCGAATTGAACCTGT
205. GU198370.1/China/C2/FY08-C30/2008 (C4)	CAAACACCGAATTGAACCTGT
206. GU198367.1/China/Anhui/FY08-C30-P2/2008/C4a/2008 (C4)	CAAACACCGAATTGAACCTGT
207. GU198369.1/China/C1/FY08-C30-P11/2008 (C4)	CAAACACCGAATTGAACCTGT
208. GU198368.1/China/C1/FY08-C30-P9/2008 (C4)	CAAACACCGAATTGAACCTGT
209. FJ439769.1/China/Anhui/Fuyang-0805/2008/C4a/2008 (C4)	CAAACACCGAATTGAACCTGT
210. EU703814.1/China/Anhui/2008/EV71/Fuyang.Anhui.P.R.C/17.08/3/2008/C4a/2008 (C4)	CAAACACCGAATTGAACCTGT
211. EU703812.1/China/Anhui/EV71/Fuyang.Anhui.P.R.C/17.08/1/2008/C4a/2008 (C4)	CAAACACCGAATTGAACCTGT
212. JX678875.1/China/Anhui/FY17.08-4/AH/CHN/2008/C4a/2008 (C4)	CAAACACCGAATTGAACCTGT
213. FJ158601.1/China/DTID/ZJU-74/2008 (C4)	CAAACACCGAATTGAACCTGT
214. FJ158600.1/China/Zhejiang/DTID/ZJU-62/2008/C4a/2008 (C4)	CAAACACCGAATTGAACCTGT

	50 4,460 4,470
Consensus	CAAACACCGAATTGAACCTGT
	FL10F; Primer Bind
214. FJ158600.1/China/Zhejiang/DTID/ZJU-62/2008/C4a/2008 (C4)	CAAACACCGAATTGAACCTGT
215. FJ606448.1/China/BJ08-Z011-4/C4/2008 (C4)	CAAACACCGAATTGAACCTGT
216. EU703813.1/China/Anhui/EV71/Fuyang.Anhui.P.R.C/17.08/2/2008/C4a/2008 (C4)	CAAACACCGAATTGAACCTGT
217. JX678879.1/China/Anhui/FY17.08-8/AH/CHN/2008/C4a/2008 (C4)	CAAACACCGAATTGAACCTGT
218. JX678877.1/China/Anhui/FY17.08-6/AH/CHN/2008/C4a/2008 (C4)	CAAACACCGAATTGAACCTGT
219. JX678876.1/China/Anhui/FY17.08-5/AH/CHN/2008/C4a/2008 (C4)	CAAACACCGAATTGAACCTGT
220. JF799986.1/China/LabstrainGuangdong/2009 (C4)	CAAACACCGTATTGAACCTGT
221. JF738002.1/Thailand/THA-EV71-044/C4/2009 (C4)	CAAACACCGTATTGAACCTGT
222. FJ607337.1/China/Shenzhen/121/SHENZHEN/08/China/HFMD-Fatal/2008/C4a/2008 (C4)	CAAACACCGAATTGAACCTGT
223. FJ606450.1/China/BJ08-Z025-5/2008 (C4)	CAAACACCGAATTGAACCTGT
224. HM622391.1/Taiwan/2008-00643/C2-like/2008 (C2)	CAAACACCGTATTGAACCTGT
225. JQ280307.1/Taiwan/3149/C2/2008 (C2)	CAAACACCGTATTGAACCTGT
226. HM622392.1/Taiwan/2008-07776/C2-like/2008 (C2)	CAAACACCGTATTGAACCTGT
227. EU527983.1/Taiwan/2007-07364/2007/C5/2007 (C5)	CAAACACCGTATTGAACCTGT
228. EF063152.1/Taiwan/E2005125-TW (C5)	CAAACACCGTATTGAACCTGT
229. DQ341355.1/SouthKorea/06-KOR-00/C3/2000 (C3)	CAAACACCGAATTGAACCTGT
230. DQ341356.1/SouthKorea/03-KOR-00/C3/2000 (C3)	CAAACACCGAATTGAACCTGT
231. AF119795.2/Taiwan/TW/2272/98/C/1998 (UNKNOWN)	CAAACACCGTATTGAACCTGT
232. AF119796.3/Taiwan/TW/2086/98/C2/1998 (C2)	CAAACACCGTATTGAACCTGT
233. DQ060149.1/Taiwan/pinf7-54A/C2/2005 (C2)	CAAACACCGTATTGAACCTGT
234. AF304457.1/Taiwan/5746/98/C2/1998 (C2)	CAAACACCGTATTGAACCTGT
235. AF176044.1/Taiwan/1245a/98/tw (C2)	CAAACACCGTATTGAACCTGT
236. JN544418.1/China/4643-TW98/C2/1998 (C2)	CAAACACCGTATTGAACCTGT
237. AF304458.1/Taiwan/Tainan/4643/98/C2 (C2)	CAAACACCGTATTGAACCTGT
238. AF304459.1/Taiwan/Tainan/6092/98/C2 (C2)	CAAACACCGTATTGAACCTGT
239. N544419.1/China/mousestrain/MP4/C2/2004 (C2)	CAAACACCGTATTGAACCTGT
240. AF136379.1/NCKU9822 (C2)	CAAACACCGTATTGAACCTGT
241. AB550333.1/Japan/Labstrain/1095-LPS1/C2/1997 (C2)	CAAACACCGTATTGAACCTGT
242. AB550332.1/Japan/1095-org/C2/1997 (C2)	CAAACACCGTATTGAACCTGT
243. JN992283.1/Australia/0964/SYD/98 (C2)	CAAACACCGTATTGAACCTGT
244. JX025559.1/Australia/Labstrain/LAZ60-TR.C2/2010 (C2)	CAAACACCGTATTGAACCTGT
245. DQ381846.1/Australia/6F/AUS/6/99/C2/1999 (C2)	CAAACACCGTATTGAACCTGT
246. DQ341357.1/Australia/7F-AUS-6-99 (C2)	CAAACACCGTATTGAACCTGT
247. AB575939.1/Netherlands/365/C2/2000 (C2)	CAAACACCGTATTGAACCTGT
248. HQ647173.1/Canada/EV003-07/2006 (C2)	CAAACACCGTATTGAACCTGT
249. HQ647168.1/Canada/EV92-07/2007 (C2)	CAAACACCGTATTGAACCTGT
250. HQ647174.1/Cana/EV123-07/2007 (C2)	CAAACACCGTATTGAACCTGT
251. HQ647170.1/Canada/EV124-07/2007 (C2)	CAAACACCGTATTGAACCTGT
252. HQ647176.1/Canada/EV049-07/2007 (C2)	CAAACACCGTATTGAACCTGT
253. HQ647169.1/Canada/EV090-07/2007 (C2)	CAAACACCGTATTGAACCTGT
254. HQ647178.1/Canada/EV082-07/2007 (C2)	CAAACACCGTATTGAACCTGT
255. HQ647177.1/Canada/EV073-07/2007 (C2)	CAAACACCGTATTGAACCTGT
256. HQ647175.1/Canada/EV053-07/2007 (C2)	CAAACACCGTATTGAACCTGT
257. HQ647167.1/Canda/EV054-07/2007 (C2)	CAAACACCGTATTGAACCTGT
258. FJ172159.1/Singapore/NUH0075/SIN/08/2008 (C2)	CAAACACCGTATTGAACCTGT
259. AB575942.1/Netherlands/3692/C2/2007 (C2)	CAAACACCGTATTGAACCTGT
260. AB575941.1/Netherlands/2485/C2/2007 (C2)	CAAACACCGTATTGAACCTGT
261. JN835312.1/France//MRS/09/3663/C3/2009 (C3)	CAAACACCGTATTGAACCTGT
262. AB575948.1/Netherlands/10118/C2/2010 (C2)	CAAACACCGTATTGAACCTGT
263. AB575938.1/Netherlands/10098/C1/2010 (C1)	CAAACACCGTATTGAACCTGT
264. JF738000.1/Thailand/THA-EV71-002/C1/2009 (C1)	CAAACACCGTATTGAACCTGT
265. EU414335.2/Switzerland/PLday1-CH-06/C1 (C1)	CAAACACCGTATTGAACCTGT
266. EU414334.2/Switzerland/PLday4-CH-06/C1 (C1)	CAAACACCGTATTGAACCTGT
267. EU414333.2/Switzerland/CSF-CH-06/C1 (C1)	CAAACACCGTATTGAACCTGT
268. EU414331.2/Switzerland/LR-CH-06/C1 (C1)	CAAACACCGTATTGAACCTGT
269. DQ341360.1/Malaysia/J115-MAL-01 (C1)	CAAACACCGTATTGAACCTGT
270. DQ341358.1/Malaysia/S40221-SAR-00/C1/2000 (C1)	CAAACACCGTATTGAACCTGT
271. AB550341.1/Malaysia/Labstrain/KED005-LPS2/C1/1997 (C1)	CAAACACCGTATTGAACCTGT
272. AB550340.1/Malaysia/KED005-org/C1/1997 (C1)	CAAACACCGTATTGAACCTGT
273. DQ452074.1/Norway/804/NO/03/C1/2003 (C1)	CAAACACCGTATTGAACCTGT
274. DQ341361.1/Australia/1M-AUS-12-00/C1 (C1)	CAAACACCGTATTGAACCTGT
275. AB575937.1/Netherlands/1416/C1/2001 (C1)	CAAACACCGTATTGAACCTGT
276. DQ341359.1/Malaysia/S10862-SAR-98/C1/1998 (C1)	CAAACACCGTATTGAACCTGT
277. HQ647172.1/Canada/EV063-04/1994 (C1)	CAAACACCGTATTGAACCTGT
278. AB575936.1/Netherlands/9612/C1/1991 (C1)	CAAACACCGTATTGAACCTGT
279. AB575935.1/Netherlands/480/C1/1991 (C1)	CAAACACCGTATTGAACCTGT

	7,420 · 7,430 · 7,440
Consensus	GGTAAATTTGTTATAACCAGAATAGC
	FL3R2
1. GU434678.1/China/Hubei/EV71-Hubei-09-China/B5/2009 (B5)	GGTAAATTTGTTATAACCAGAATAGC
2. AB204853.1/BrCr-ts/A (A)	GGTAAATTTGTTATAACCAGAATAGC
3. AB575911.1/Netherlands/10076/B0/1966 (B0)	GGTAATTATACCATA
4. AB575912.1/Netherlands/10857/B0/1966 (B0)	GT--------TTCCAGCCC
5. DQ341354.1/Singapore/3799-SIN-98/B3/1998 (B3)	GGTAAATTTGTTATAACCAGAATAGC
6. DQ341368.1/Malaysia/MY104-9-SAR-97/B3 (B3)	GGTAAATTTGTTATAACCAGAATAGC
7. DQ341367.1/Malaysia/MY821-3-SAR-97/B3/1997 (B3)	GGTAAATTTGTTATAACCAGAATAGC
8. AB550335.1/Malaysia/Labstrain/SK-EV006-LPS1/B3/1997 (B3)	GGTAAATTTGTTATAACCAGAATAGC
9. AB550334.1/Malaysia/SK-EV006-org/B3/1997 (B3)	GGTAAATTTGTTATAACCAGAATAGC
10. AB469182.1/Malaysia/SK-EV006/Malaysia/97 (B3)	GGTAAATTTGTTATAACCAGAATAGC
11. JQ950555.1/Australia26M/AUS/4/99/GuaR1/B3/2010 (B3)	
12. EU364841.1/Australia/26M/AUS/4/99/B3/1999 (B3)	GGTAAATTTGTTATAACCAGAATAGC
13. EF373575.1/Taiwan/E2002042-TW-CDC (B4)	GGTAAATTTGTTATAACCAGAATAGC
14. FJ357377.1/Taiwan/S0296-TW00/B4/2000 (B4)	GGTAAATTTGTTATAACCAGAATAGC
15. FJ357376.1/Taiwan/S0318-TW01/B4/2001 (B4)	GGTAAATTTGTTATAACCAGAATAGC
16. DQ341366.1/Malaysia/SB2864-SAR-00/B4/2000 (B4)	GGTAAATTTGTTATAACCAGAATAGC
17. DQ341365.1/Malaysia/PP37-MAL-01/B4/2001 (B4)	GGTAAATTTGTTATAACCAGAATAGC
18. AF316321.2/Singapore/5865/sin/000009/B4 (B4)	GGTAAATTTGTTATAACCGGAATAGC
19. AF352027.1/Singapore/5666/sin/002209/B4 (B4)	GGTAAATTTGTTATAACCGGAATAGC
20. FJ357375.1/Taiwan/N7008-TW99/B4/1999 (B4)	GGTAAATTTGTTATAACCAGAATAGC
21. AB550337.1/Japan/Labstrain/C7/Osaka-LPS1/B4/1997 (B4)	GGTAAATTTGTTATAACCAGAATAGC
22. AB550336.1/Japan/C7/Osaka-org/B4/1997 (B4)	GGTAAATTTGTTATAACCAGAATAGC
23. FJ461781.1/Singapore/NUH0083/SIN/08/B5/2008 (B5)	GGTA
24. JF738001.1/Thailand/THA-EV71-019/B5/2009 (B5)	GGTAAATTTGTTATAACCAAAATAGC
25. DQ341364.1/Singapore/5511-SIN-00 (B5)	GGTAAATTTGTTATAACCAGAATAGC
26. JN992282.1/Brunei/BRU/2006/35334 (B5)	GGTAAATTTGTTATAACCAGAATAGC
27. FJ357378.1/Taiwan/N2838-TW03/B5/2003 (B5)	GGTAAATTTGTTATAACCAGAATAGC
28. DQ341363.1/Malaysia/S19841-SAR-03/B5/2003 (B5)	GGTAAATTTGTTATAACCAGAATAGC
29. DQ341362.1/Malaysia/SB12736-SAR-03/B4/2003 (B4)	GGTAAATTTGTTATAACCAGAATAGC
30. JN964686.1/China/Fujian/EV71/Xiamen/2009/B5/2009 (B5)	GGTAAATTTGTTATAACCAGAATAGC
31. GQ231942.1/Taiwan/TW/96016/08/B5 (B5)	GGTAAATTTGTTATAACCGGAATAGC
32. GQ231925.1/Taiwan/TW/1101/08/B5 (B5)	GGTAAATTTGTTATAACCGGAATAGC
33. GQ231934.1/Taiwan/TW/70811/08/B5 (B5)	GGTAAATTTGTTATAACCGGAATAGC
34. GQ231943.1/Taiwan/TW/96022/08/B5 (B5)	GGTAAATTTGTTATAACCGGAATAGC
35. GQ231935.1/Taiwan//TW/70886/08/B5 (B5)	GGTAAATTTGTTATAACCGGAATAGC
36. GQ231941.1/Taiwan/TW/96002/08/B5 (B5)	GGTAAATTTGTTATAACCGGAATAGC
37. EU527985.1/Taiwan/2007-08747/2007/B5/2007 (B5)	
38. FJ357385.1/Taiwan/M0380-TW08/B5/2008 (B5)	GGTAAATTTGTT-TAACCAGAATAGC
39. HM622390.1/Taiwan/2009-03531/B5/2009 (B5)	
40. GQ231936.1/Taiwan/TW/70902/08/B5 (B5)	GGTAAATTTGTTATAACCGGAATAGC
41. AB575927.1/Netherlands/11316/B2/1986 (B2)	
42. AB575928.1/Netherlands/11590/B2/1986 (B2)	
43. AB575923.1/Netherlands/20233/B2/1983 (B2)	
44. U22522.1/USA/MS/7423/87/B2/1987 (B2)	GGTAAATTTGTTATAACCAGAATAGC
45. FJ357384.1/Taiwan/266-TW86/B1/1986 (B1)	GGTAAATTTGTTATAACCAGAATAGC
46. FJ357383.1/Taiwan/252-TW86/B1/1986 (B1)	GGTAAATTTGTTATAACCAGAATAGC
47. FJ357380.1/Taiwan/237-TW86/B1/1986 (B1)	GGTAAATTTGTTATAACCAGAATAGC
48. FJ357379.1/Taiwan/236-TW86/B1/1986 (B1)	GGTAAATTTGTTATAA-CAGAATAGC
49. FJ357382.1/Taiwan/238-TW86/B1/1986 (B1)	GGTAAATTTGTTATAACCAGAATAGC
50. FJ357381.1/Taiwan/244-TW86/B1/1986 (B1)	GGTAAATTTGTTATAACCAGAATAGC
51. AB575914.1/Netherlands/17000/B1/1971 (B1)	
52. AB575913.1/Netherlands/11977/B1/1971 (B1)	
53. AB575918.1/Netherlands/20574/B1/1978 (B1)	
54. AB575917.1/Netherlands/10196/B1/1977 (B1)	
55. HQ189392.1/Hungary/HUN/1978/1978 (B1)	GGTAAATTTGTTATAACCAGAATAGC
56. AB575916.1/Netherlands/16173/B1/1976 (B1)	GGTAAATTTGTTATA
57. AB575915.1/Netherlands/9443/B1/1974 (B1)	
58. AB482183.1/Japan/Nagoya/B1/1973 (B1)	GGTAAATTTGTTATAACCAGAATAGC
59. HQ423143.1/China/Yunnan/KM186/09/2009/C4a/2009 (C4)	GGTAAATTTGTTATAACCAGAATAGC
60. HQ423142.1/China/KMM/09/2009 (C4)	GGTAAATTTGTTATAACCAGATTAGC
61. JQ742001.1/China/Guangxi/AFP2001064/EV71/GX/CHN/2001/C4b/2001 (C4)	GGTAAATTTGTTATAACCAGAATAGC
62. EU131776.1/Taiwan/N3340-TW-02/C4b/2002 (C4)	GGTAAATTTGTTATAACCAGAATAGC
63. JX678885.1/China/Shanghai/SH-17/SH/CHN/2002/C4b/2002 (C4)	GGTAAATTTGTTATAACCAGAATAGC
64. JQ742002.1/China/AFP2001071/EV71/GX/CHN/2001/C4b/2001 (C4)	GGTAAATTTGTTATAACCAGAATAGC
65. GU350629.1/China/AnHui/Fuyang-0805a (C4)	
66. FJ194964.1/China/Guangdong/EV71/GDFS/3/2008/C4b/2008 (C4)	GGTAAATTTGTTATAACCAGAATAGC
67. GQ994989.1/China/Chongqing/Chongqing1-09-China/2009/C4b/2009 (C4)	GGTAAATTTGTTATAACCAGAATAGC
68. HM807310.1/Taiwan/cmuh-050530-5/2005 (C4)	GGTAAATTTGTTATAACCAGAATAGC
69. AY465356.1/China/Shenzhen/SHZH03/C4b/2003 (C4)	GGTAAATTTGTTATAACCAGAATAGC
70. JX678874.1/China/Chongqing/CQ03-1/CQ/CHN/2003/C4b/2003 (C4)	GGTAAATTTGTTATAACCAGAATAGC
71. HQ647171.1/Canada/EV141-06/2006/C4a/2006 (C4)	GGTAAATTTGTTATAACCAGAATAGC
72. HQ647180.1/Canada/EV034-06/2006 (C4)	GGTAAATTTGTTATAACCAGAATAGC

Sequence	7,420 – 7,440
Consensus	GGTAAATTTGTTATAACCAGAATAGC
	FL3R2
71. HQ647171.1/Canada/EV141-06/2006/C4a/2006 (C4)	GGTAAATTTGTTATAACCAGAATAGC
72. HQ647180.1/Canada/EV034-06/2006 (C4)	GGTAAATTTGTTATAACCAGAATAGC
73. HQ647179.1/Canada/EV144-06/2006 (C4)	GGTAAATTTGTTATAACCAGAATAGC
74. JX678886.1/China/Shanghai/SH-6/SH/CHN/2002/C4b/2002 (C4)	GGTAAATTTGTTATAACCAGAATAGC
75. AB550339.1/Japan/Labstrain/75-Yamagata-LPS1/C4/2003 (C4)	GGTAAATTTGTTATAACCAGAATAGC
76. AB550338.1/Japan/75-Yamagata-org/C4/2003 (C4)	GGTAAATTTGTTATAACCAGAATAGC
77. DQ133459.1/Taiwan/1235/C4/2004 (C4)	GGTAAATTTGTTATAACCAGAATAGC
78. DQ133458.1/Taiwan/984/C4/2004 (C4)	GGTAAATTTGTTATAACCAGAATAGC
79. FJ357373.1/Taiwan/S0584-TW04/C4/2004 (C4)	GGTAAATTTGTTATAACCAGAATAGC
80. EF373576.1/Taiwan/E2004104-TW-CDC (C4)	GGTAAATTTGTTATAACCAGAATAGC
81. GQ231938.1/TaiwanTW/71552/05 (C4)	GGTAAATTTGTTATAACCGGAATAGC
82. GQ231926.1/Taiwan/TW/1956/05/C4a/2005 (C4)	GGTAAATTTGTTATAACCGGAATAGC
83. GQ231940.1/Taiwan/TW/72232/04/C4 (C4)	GGTAAATTTGTTATAACCGGAATAGC
84. GQ231937.1/Taiwan/TW/71428/05/C4 (C4)	GGTAAATTTGTTATAACCGGAATAGC
85. GQ231939.1/Taiwan/TW/71595/04/C4 (C4)	GGTAAATTTGTTATAACCGGAATAGC
86. GQ231929.1/Taiwan/TW/2728/04/C4a/2004 (C4)	GGTAAATTTGTTATAACCAGAATAGC
87. FJ357374.1/Taiwan/N2121-TW05/C4/2005 (C4)	GGTAAATTTGTTATAACCAGAATAGC
88. GQ231931.1/Taiwan/TW/2824/04/C4 (C4)	GGTAAATTTGTTATAACCGGAATAGC
89. GQ231928.1/Taiwan/TW/2639/04/C4 (C4)	GGTAAATTTGTTATAACCGGAATAGC
90. GQ231927.1/Taiwan/TW/2429/04/C4 (C4)	GGTAAATTTGTTATAACCGGAATAGC
91. GQ231930.1/Taiwan/TW/2815/04/C4 (C4)	GGTAAATTTGTTATAACCGGAATAGC
92. JQ965759.1/Vietnam/540V/VNM/05/C4/2005 (C4)	GGTAAATTTGTTATAACCAGAATAGC
93. GQ231932.1/Taiwan/TW/2871/04/C4 (C4)	GGTAAATTTGTTATAACCGGAATAGC
94. GQ994992.1/China/Henan/Henan2-09-China/2009/C4a/2009 (C4)	GGTAAATTTGTTATACCCGAAATAG
95. GQ279369.1/China/SZ/HK08-5/2008 (C4)	GGTAAATTTGTTATAACCAGAATAGC
96. JX244184.1/China/SDLY48/C/2009 (C4)	GGTAAATTTGTTATAACCAGAATAGC
97. FJ606447.1/China/Beijing/BJ08-Z004-3/2008/C4a/2008 (C4)	GGTAAATTTGTTATAACCAGAATAGC
98. JQ681218.1/China/Henan/H8-1/C4a/2008 (C4)	GGTAAATTTGTTATAACCAGAATAGC
99. GU196833.1/China/Henan1-09-China/2009 (C4)	GGTAAATTTGTTATACACAAAAT
100. HQ129932.1/China/BJ06-SJS06/06/C4/2006 (C4)	
101. FJ607336.1/China/28/SHENZHEN/08/China/HFMD/2008/2008 (C4)	GGTAAATTTGTTATAACCAGAATAGC
102. FJ828519.1/China/BJ08/C4/2008 (C4)	GGTAAATTTGTTATAACCAGAATAGC
103. FJ360545.1/China/GZ-08-02/C4/2008 (C4)	GGTAAATTTGTTATAACCAGAATAGC
104. JX244182.1/China/SDLY1/2009 (C4)	GGTAAATTTGTTATAGCCAGAATAGC
105. JF820312.1/China/LCH01/C4/2010 (C4)	
106. HM002488.1/China/BJ366/2009 (C4)	GGTAAATTTGTTATAACCAGAATAGC
107. GQ231933.1/Taiwan/TW/70516/08/C4a/2008 (C4)	GGTAAATTTGTTATAACCGGAATAGC
108. KC109780.1/China/202/Jingdezhen/China/HFMDSevere/2011/C4/2011 (C4)	GGTAAATTTGTTATAACCAGAATAGC
109. HM002485.1/China/BJ972008 (C4)	GGTAAATTTGTTATAACCAGAATAGC
110. GU366191.1/China/Henan/Henan10-08-China/2008/C4a/2008 (C4)	GGTAAATTTGTTATAACCAGAATAGC
111. FJ713137.1/Shanghai/Shanghai-036-2009/C4a/2009 (C4)	GGTGAATTTGTTATAACCAGAATAGG
112. EU753384.2/China/Shandong/522-04T/SD/CHN/07/C4a/2007 (C4)	GGTAAATTTGTTATAACCAGAATAAC
113. HM003207.1/China/87-2008-Xi'an-Shaanxi/2008 (C4)	GGTAAATTTGTTATAACCAGAATAGC
114. EU753365.2/China/Shandong/518-03F/SD/CHN/07/C4a/2007 (C4)	GGTAAATTTGTTATAACCAGAATAGC
115. EU753407.2/China/Shandong/TC03F/SD/CHN/07/C4a/2007 (C4)	GGTAAATTTGTTATAACCAGAATAGC
116. GQ994988.1/China/Anhui/Anhui1-09-China/C4a/2009 (C4)	GGTAAATTTGTTATCC
117. JQ708210.1/China/isolateTS/C4/2009 (C4)	GGTAAATTTGTTATAACCAGAATAGC
118. HQ611148.1/China/AH08/06/C4/2008 (C4)	GGTAAATTTGTTATAACCAGAATAGC
119. EU753398.2/China/Shandong/523-07T/SD/CHN/07/2007 (C4)	GGTAAATTTGTTATAACCAGAATAGC
120. EU753397.2/China/Shandong/523-05T/SD/CHN/07/C4a/2007 (C4)	GGTAAATTTGTTATAACCAGAATAGC
121. JX678882.1/China/Shandong/SD07-1/SD/CHN/2007/C4a/2007 (C4)	GGTAAATTTGTTATAACCAGAATAGC
122. EU753375.2/China/Shandong/521-18S/SD/CHN/07/C4a/2007 (C4)	GGTAAATTTGTTATAACCAGAATAGC
123. HM002487.1/China/BJ303/2008 (C4)	GGTAAATTTGTTATAACCAGAATAGC
124. JX678878.1/China/Anhui/FY17.08-7/AH/CHN/2008/C4a/2008 (C4)	GGTAAATTTGTTATAACCAGAATAGC
125. JX025561.1/China/FY7VP5/AH/CHN/2008 (C4)	GGTAAATTTGTTATAACCAGAATAGC
126. JQ316638.1/China/HQ09231463/2011 (C4)	GGTAAATTTGTTATAACCAGAATAGC
127. HQ426649.1/China/Anhui/2010FJLY008/2010/C4a/2010 (C4)	GGTAAATTTGTTATAACCAGAATAGC
128. GQ994991.1/China/Chongqing3-09-China/2009 (C4)	GGTAAATTTGTTAT-ACCCGAATAAC
129. HM053670.1/China/strain-BJ393/2009 (C4)	GGTAAATTTGTTATAACCAGAATAGC
130. FJ360544.1/China/GZ-08-01/C4/2008 (C4)	GGTAAATTTGTTATAACCAGAATAGC
131. HQ998852.1/China/Henan/EV71/Henan/106/2009/C4a/2009 (C4)	GGTAAATTTGTTATAACA-GAATAGC
132. JX244187.1/China/SDLY153/2010 (C4)	GGTAAATTTGTTATAACCAGAATAGC
133. GQ994990.1China/Chongqing2-09-China/C4/2009 (C4)	GGTAAATTTGTTATACCCAAAATAGC
134. EU864507.1/China/Zhejiang/EV71/Zhejiang08/2008/C4a/2008 (C4)	GGTAAATTTGTTATAACCAGAATAGC
135. FJ194965.1/China/EV71/GDSG/17/2008/C/2008 (C4)	GGTAAATTTGTTATAACCAGAATAGC
136. FJ607334.1/China/SHENZHEN/08/China/HFMD/2008/2008 (C4)	GGTAAATTTGTTATAACCAGAATAGC
137. GQ279370.1/China/SZ/HK08-6/2008 (C4)	GGTAAATTTGTTATAACCAGAATAGC
138. FJ607335.1/China/4/SHENZHEN/08/China/HFMD/2008/2008 (C4)	GGTAAATTTGTTATAACCAGAATAGC
139. JQ736684.2/China/SHAPHC695F/SH/CHN/10/C4a/2010 (C4)	GGTAAATTTGTTATAACCAGAATAGC
140. JQ074187.1/China/Jinan1002/2010 (C4)	GGTAAATTTGTTATAACCAGAATAGC
141. JQ074189.1/China/Jinan1005/2010 (C4)	GGTAAATTTGTTATAACCAGAATAGC
142. JQ074188.1/China/EV71/Jinan1004/2010 (C4)	GGTAAATTTGTTATAACCAGAATAGC
143. JF830007.1/China/Zhejiang/EV71/Ningbo.CHN/065/2010/C4a/2010 (C4)	GGTAAATTTGTTATAACCAGAGTAGC

	7,420 · 7,430 · 7,440
Consensus	GGTAAATTTGTTATAACCAGAATAGC
	FL3R2
142. [illegible]	[illegible]
143. JF830007.1/China/Zhejiang/EV71/Ningbo.CHN/065/2010/C4a/2010 (C4)	GGTAAATTTGTTATAACCAGAGTAGC
144. JX111893.1/China/GD-DG2011-9/C4/2011 (C4)	GGTAAATTTGTTATAACCAGAATAGC
145. JX111891.1/China/GD-DG2011-5/C4/2011 (C4)	GGTAAATTTGTTATAACCAGAATAGC
146. JX111889.1/China/GD-DG2011-2/C4/2011 (C4)	GGTAAATTTGTTATAACCAGAATAGC
147. JX111888.1/China/GD-DG2011-1/C4/2011 (C4)	GGTAAATTTGTTATAACCAGAATAGC
148. JX111892.1/China/GD-DG2011-8/C4/2011 (C4)	GGTAAATTTGTTATAACCAGAATAGC
149. JX111890.1/China/GD-DG2011-3/C4/2011 (C4)	GGTAAATTTGTTATAACCAGAATAGC
150. JQ806378.1/China/Jiangxi/35/Jingdezhen/China/HFMDSevere/2011/C4a/2011 (C4)	GGTAAATTTGTTATAACCAGAATAGC
151. JX017384.1/China/01011Y/2011 (C4)	GGTAAATTTGTTATAACCAGAATAGC
152. HQ891925.1/China/Shanghai-36-2009/C4/2009 (C4)	GGTAAATTTGTTATAACCAGAATAGC
153. JX244186.1/China/SDLY107/2010 (C4)	GGTAAATTTGTTATAACCAGAATAGC
154. HQ850973.1/China/003/XM/2010/2010 (C4)	
155. FJ360546.1/China/GZ-08-03/C4/2008 (C4)	GGTAAATTTGTTATAACCAGAATAGC
156. HQ891926.1/China/Shanghai-51-2009/C4/2009 (C4)	GGTAAATTTGTTATAACCAGAATAGC
157. GQ892830.1/China/GX/LZ (C4)	GGTAAATTTGTTATAACCAGAATAGC
158. JN020147.1/China/Henan/Luoyang/2011/C4a/2011 (C4)	GGTAAATTTGTTATAACCAGAATAGC
159. JQ517316.1/China/Henan/Kaifeng/2010/C4a/2010 (C4)	GGTAAATTTGTTATAACCAGAATAGC
160. JN052925.1/China/Henan/Nanyang/2011-China/2011/C4a/2011 (C4)	GGTAAATTTGTTATAACCAGAATAG
161. HQ828086.1/China/Zhejiang/NBChina01/2010/C4a/2010 (C4)	GGTAAATTTGTTATAACCAGAATAGC
162. JX986739.1/China/Wuhan1143/HuB/CHN/2011/C4/2011 (C4)	GGTAAATTTGTTATAACCAGAATAGC
163. HQ407557.1/China/Liangning/LN009/2010/C4a/2010 (C4)	GGTAAATTTGTTATAACCAGAATAGC
164. JQ074190.1/China/Shandong/Jinan1006/2010/C4a/2010 (C4)	GGTAAATTTGTTATAACCAGAATAGC
165. JN001860.1/China/Zhejiang/NB/2010/01/C4a/2010 (C4)	GGTAAATTTGTTATAACCAGAATAGC
166. JQ804832.1/China/EV71/Hubei-XF/CHN/2010/C4/2010 (C4)	GGTAAATTTGTTATAACCAGAATAGC
167. JQ514785.1/China/BC08/C4a/2008 (C4)	GGTAAATTTGTTATAACCAGAATAGC
168. HM053669.1/China/BJ293/2008 (C4)	GGTAAATTTGTTATAACCAGAATAGC
169. HM002484.1/China/BJ67/2008 (C4)	GGTAAATTTGTTATAACCAGAATAGC
170. GU396280.1/China/Gansu/EV71/Lanzhou01/GS/CHN/09/C4a/2009 (C4)	GGTAAATTTGTTATAACCAGAATAGC
171. HQ400942.1/China/HZ08/Hangzhou/2008/C4/2008 (C4)	GGTAAATTTGTTATAACCAGAATAGC
172. HQ891929.1/China/Shanghai-118-2009/C4/2009 (C4)	GGTAAATTTGTTATAACCAGAATAGG
173. FJ607338.1/China/Shenzhen/605/SHENZHEN/08/China/HFMD/C4a/2008 (C4)	GGTAAATTTGTTATAACCAGAATAGC
174. JX986737.1/China/Wuhan1042/HuB/CHN/2011/C4/2011 (C4)	GGTAAATTTGTTATAACCAGAATAGC
175. HQ891927.1/China/Shanghai-64-2009/C4/2009 (C4)	GGTAAATTTGTTATAACCAGGGTAGC
176. HQ891928.1/China/Shanghai-117-2009/C4/2009 (C4)	GGTAAATTTGTTATAACCAGAGTAG
177. HQ891924.1/China/Shanghai-28-2009/C4/2009 (C4)	GGTAAATTTGTTATAACCAGAGTAGC
178. HQ891923.1/China/Shanghai/Shanghai-27-2009/C4a/2009 (C4)	GGTAAATTTGTTATAACCAGAGTAG
179. HM002489.1/China/Beijing/Beijing398/2009/C4a/2009 (C4)	GGTAAATTTGTTATAACCAGAATAGC
180. JN256063.1/China/RDcells/M186-1179F/HeN/CHN/2009/C4a (C4)	GGTAAATTTGTTATAACCAGAATAGC
181. JN256062.1/China/RDcells/M183-1176F/HeN/CHN/2009/C4a (C4)	GGTAAATTTGTTATAACCAGAATAGC
182. JN256064.1/China/Henan/RDcells/M188-1181F/HeN/CHN/2009/C4a/2009 (C4)	GGTAAATTTGTTATAACCAGAATAGC
183. JX678881.1/China/Henan/HeN09-17/HeN/CHN/2009/C4a/2009 (C4)	GGAAAATTGGTTATAACCAGAATAGC
184. JX678880.1/China/Henan/HeN09-1/HeN/CHN/2009/C4a/2009 (C4)	GGTAAATTGGTTATAACCAGAATAGC
185. EU812515.1/China/Anhui/FY23/2008/C4a/2008 (C4)	GGTAAATTTGTTATAACCAGAATAGC
186. GU459071.1/China/FY23-K14/C4/2008 (C4)	GGTAAATTTGTTATAACCAGAATAGC
187. GU459070.1/China/FY23-K12/C4/2008 (C4)	GGTAAATTTGTTATAACCAGAATAGC
188. JX244185.1/China/SDLY96/2009 (C4)	GGTAAATTTGTTATAACCAGAATAGC
189. JX244183.1/China/SDLY11/2009 (C4)	GGTAAATTTGTTATAACCAGAATAGC
190. JN256061.1/China/RDcells/G398-1037F/HeN/CHN/2009/C4a (C4)	GGTAAATTTGTTATAACCAGAATAGC
191. HQ825317.1/China/Shandong/EV71/JN200804/2008/C4a/2008 (C4)	GGTAAATTTGTTATAACCAGAATAGC
192. JF913464.1/China/Shandong/EV71/JN200803/2008/C4a/2008 (C4)	GGTAAATTTGTTATAACCAGAATAGC
193. JN256060.1/China/RDcellsG333-972F/HeN/CHN/2009/C4a (C4)	GGTAAATTTGTTATAACCAGAATAGC
194. JN256059.1/China/Henan/RDcells/G288-927F/HeN/CHN/2009/C4a/2009 (C4)	GGTAAATTTGTTATAACCAGAATAGC
195. JX678884.1/China/Shandong/SD09-21/SD/CHN/2009/C4a/2009 (C4)	GGTAAATTTGTTATAACCAGAATAGC
196. JX678883.1/China/Shandong/SD09-14/SD/CHN/2009/C4a/2009 (C4)	GGTAAATTGGTTATAACCAGAATAGC
197. JQ319054.1/China/Beijing/BJ09/07/C4a/2009 (C4)	GGTAAATTTGTTATAACCAGAATAGC
198. JF820315.1/China/AH01/C4/2010 (C4)	-----------------CACAAACGC
199. JF820314.1/China/BJ02/C4/2010 (C4)	
200. JQ708209.1/China/isolateBJ/C4/2010 (C4)	GGTAAATTTGTTATAACCAGAATAGC
201. JF820316.1/China/BJ01/C4/2010 (C4)	
202. JF820313.1/China/LCH02/C4/2010 (C4)	
203. FJ606449.1/China/BJ08-Z020-1/2008 (C4)	GGTAAATTTGTTATAACCAGAATAGC
204. GU198371.1/China/FY08-C30-P14/2008 (C4)	GG-AAATT----------GA
205. GU198370.1/China/C2/FY08-C30/2008 (C4)	GG-AAATT----------GA
206. GU198367.1/China/Anhui/FY08-C30-P2/2008/C4a/2008 (C4)	GGTAAATTTGTTATACCAGA
207. GU198369.1/China/C1/FY08-C30-P11/2008 (C4)	GGTAAATTTGTTATA
208. GU198368.1/China/C1/FY08-C30-P9/2008 (C4)	GGTAAATTTGTTATA-CCAGA
209. FJ439769.1/China/Anhui/Fuyang-0805/2008/C4a/2008 (C4)	GGTAAATTTGTTATAACCAGAATAGC
210. EU703814.1/China/Anhui/2008/EV71/Fuyang.Anhui.P.R.C/17.08/3/2008/C4a/2008 (C4)	GGTAAATTTGTTATAACCAGAATAGC
211. EU703812.1/China/Anhui/EV71/Fuyang.Anhui.P.R.C/17.08/1/2008/C4a/2008 (C4)	GGTAAATTTGTTATAACCAGAATAGC
212. JX678875.1/China/Anhui/FY17.08-4/AH/CHN/2008/C4a/2008 (C4)	GGTAAATTTGTTATAACCAGAATAGC
213. FJ158601.1/China/DTID/ZJU-74/2008 (C4)	GGTAAATTTGTTATAACCAGAATAGC
214. FJ158600.1/China/Zhejiang/DTID/ZJU-62/2008/C4a/2008 (C4)	GGTAAATTTGTTATAACCAGAATAGC

	7,420 · 7,430 · 7,440
Consensus	GGTAAATTTGTTATAACCAGAATAGC
	FL3R2
214. FJ158600.1/China/Zhejiang/DTID/ZJU-62/2008/C4a/2008 (C4)	GGTAAATTTGTTATAACCAGAATAGC
215. FJ606448.1/China/BJ08-Z011-4/C4/2008 (C4)	GGTAAATTTGTTATAACCAGAATAGC
216. EU703813.1/China/Anhui/EV71/Fuyang.Anhui.P.R.C/17.08/2/2008/C4a/2008 (C4)	GGTAAATTTGTTATAACCAGAATAGC
217. JX678879.1/China/Anhui/FY17.08-8/AH/CHN/2008/C4a/2008 (C4)	GGTAAATTTGTTATAACCAGAATAGC
218. JX678877.1/China/Anhui/FY17.08-6/AH/CHN/2008/C4a/2008 (C4)	GGTAAATTTGTTATAACCAGAATAGC
219. JX678876.1/China/Anhui/FY17.08-5/AH/CHN/2008/C4a/2008 (C4)	GGTAAATTTGTTATAACCAGAATAGC
220. JF799986.1/China/LabstrainGuangdong/2009 (C4)	GGTAAATTTGTTATAACCAGAATAGC
221. JF738002.1/Thailand/THA-EV71-044/C4/2009 (C4)	GGTAAATTTGTTATAACCAGAATAGC
222. FJ607337.1/China/Shenzhen/121/SHENZHEN/08/China/HFMD-Fatal/2008/C4a/2008 (C4)	GGTAAATTTGTTATAACCAGAATAGC
223. FJ606450.1/China/BJ08-Z025-5/2008 (C4)	GGTAAATTTGTTATAACCAGAATGGC
224. HM622391.1/Taiwan/2008-00643/C2-like/2008 (C2)	
225. JQ280307.1/Taiwan/3149/C2/2008 (C2)	GGTAAATTTGTTATA-CCAGAATAGC
226. HM622392.1/Taiwan/2008-07776/C2-like/2008 (C2)	
227. EU527983.1/Taiwan/2007-07364/2007/C5/2007 (C5)	
228. EF063152.1/Taiwan/E2005125-TW (C5)	GGTAAATTTGTTATAACCAGAATAGC
229. DQ341355.1/SouthKorea/06-KOR-00/C3/2000 (C3)	GGTAAATTTGTTATAACCAGAATAGC
230. DQ341356.1/SouthKorea/03-KOR-00/C3/2000 (C3)	GGTAAATTTGTTATAACCAGAATAGC
231. AF119795.2/Taiwan/TW/2272/98/C/1998 (UNKNOWN)	GGTAAATTTGTTATAACCAGAATAGC
232. AF119796.3/Taiwan/TW/2086/98/C2/1998 (C2)	GGTAAATTTGTTATAACCAGAATAGC
233. DQ060149.1/Taiwan/pinf7-54A/C2/2005 (C2)	GGTAAATTTGTTATAACCAGAATAGC
234. AF304457.1/Taiwan/5746/98/C2/1998 (C2)	GGTAAATTTGTTATAACCAGAATAGC
235. AF176044.1/Taiwan/1245a/98/tw (C2)	GGTAAATTTGTTAT-ACCAGAATAGC
236. JN544418.1/China/4643-TW98/C2/1998 (C2)	GGTAAATTTGTTATAACCAGAATAGC
237. AF304458.1/Taiwan/Tainan/4643/98/C2 (C2)	GGTAAATTTGTTATAACCAGAATAGC
238. AF304459.1/Taiwan/Tainan/6092/98/C2 (C2)	GGTAAATTTGTTATAACCAGAATAGC
239. N544419.1/China/mousestrain/MP4/C2/2004 (C2)	GGTAAATTTGTTATAACCAGAATAGC
240. AF136379.1/NCKU9822 (C2)	GGTAAATTTGTTATAACCAGAATAGC
241. AB550333.1/Japan/Labstrain/1095-LPS1/C2/1997 (C2)	GGTAAATTTGTTATAACCAGAATAGC
242. AB550332.1/Japan/1095-org/C2/1997 (C2)	GGTAAATTTGTTATAACCAGAATAGC
243. JN992283.1/Australia/0964/SYD/98 (C2)	GGTAAATTTGTTATAACCAGAATAGC
244. JX025559.1/Australia/Labstrain/LAZ60-TR.C2/2010 (C2)	GGTAAATTTGTTATAACCAGAATAGC
245. DQ381846.1/Australia/6F/AUS/6/99/C2/1999 (C2)	GGTAAATTTGTTATAACCAGAATAGC
246. DQ341357.1/Australia/7F-AUS-6-99 (C2)	GGTAAATTTGTTATAACCAGAATAGC
247. AB575939.1/Netherlands/365/C2/2000 (C2)	
248. HQ647173.1/Canada/EV003-07/2006 (C2)	GGTAAATTTGTTATAACCAGAATAGC
249. HQ647168.1/Canada/EV92-07/2007 (C2)	GGTAAATTTGTTATAACCAGAATAGC
250. HQ647174.1/Cana/EV123-07/2007 (C2)	GGTAAATTTGTTATAACCAGAATAGC
251. HQ647170.1/Canada/EV124-07/2007 (C2)	GGTAAATTTGTTATAACCAGAATAGC
252. HQ647176.1/Canada/EV049-07/2007 (C2)	GGTAAATTTGTTATAACCAGAATAGC
253. HQ647169.1/Canada/EV090-07/2007 (C2)	
254. HQ647178.1/Canada/EV082-07/2007 (C2)	GGTAAATTTGTTATAACCAGAATAGC
255. HQ647177.1/Canada/EV073-07/2007 (C2)	GGTAAATTTGTTATAACCAGAATAGC
256. HQ647175.1/Canada/EV053-07/2007 (C2)	GGTAAATTTGTTATAACCAGAATAGC
257. HQ647167.1/Canda/EV054-07/2007 (C2)	GGTAAATTTGTTATAACCAGAATAGC
258. FJ172159.1/Singapore/NUH0075/SIN/08/2008 (C2)	
259. AB575942.1/Netherlands/3692/C2/2007 (C2)	
260. AB575941.1/Netherlands/2485/C2/2007 (C2)	
261. JN835312.1/France//MRS/09/3663/C3/2009 (C3)	----AATTTGTTATAACCAGAATAGC
262. AB575948.1/Netherlands/10118/C2/2010 (C2)	
263. AB575938.1/Netherlands/10098/C1/2010 (C1)	
264. JF738000.1/Thailand/THA-EV71-002/C1/2009 (C1)	GGTAAATTTGTTATAACCAGAATAGC
265. EU414335.2/Switzerland/PLday1-CH-06/C1 (C1)	
266. EU414334.2/Switzerland/PLday4-CH-06/C1 (C1)	
267. EU414333.2/Switzerland/CSF-CH-06/C1 (C1)	GGTAAATTTGTTATAACCAGAATAGC
268. EU414331.2/Switzerland/LR-CH-06/C1 (C1)	
269. DQ341360.1/Malaysia/J115-MAL-01 (C1)	GGTAAATTTGTTATAACCAGAATAGC
270. DQ341358.1/Malaysia/S40221-SAR-00/C1/2000 (C1)	GGTAAATTTGTTATAACCAGAATAGC
271. AB550341.1/Malaysia/Labstrain/KED005-LPS2/C1/1997 (C1)	GGTAAATTTGTTATAACCAGAATAGC
272. AB550340.1/Malaysia/KED005-org/C1/1997 (C1)	GGTAAATTTGTTATAACCAGAATAGC
273. DQ452074.1/Norway/804/NO/03/C1/2003 (C1)	GGTAAATTTGTTATAACCAGAATAGC
274. DQ341361.1/Australia/1M-AUS-12-00/C1 (C1)	GGTAAATTTGTTATAACCAGAATAGC
275. AB575937.1/Netherlands/1416/C1/2001 (C1)	
276. DQ341359.1/Malaysia/S10862-SAR-98/C1/1998 (C1)	GGTAAATTTGTTATAACCAGAATAGC
277. HQ647172.1/Canada/EV063-04/1994 (C1)	GGTAAATTTGTTATAACCAGAATAGC
278. AB575936.1/Netherlands/9612/C1/1991 (C1)	
279. AB575935.1/Netherlands/480/C1/1991 (C1)	

Supplementary Appendix Figure 1: Alignment profiles of primer binding sites
